# Elucidation of the genetic architecture of self‐incompatibility in olive: Evolutionary consequences and perspectives for orchard management

**DOI:** 10.1111/eva.12457

**Published:** 2017-05-20

**Authors:** Pierre Saumitou‐Laprade, Philippe Vernet, Xavier Vekemans, Sylvain Billiard, Sophie Gallina, Laila Essalouh, Ali Mhaïs, Abdelmajid Moukhli, Ahmed El Bakkali, Gianni Barcaccia, Fiammetta Alagna, Roberto Mariotti, Nicolò G. M. Cultrera, Saverio Pandolfi, Martina Rossi, Bouchaïb Khadari, Luciana Baldoni

**Affiliations:** ^1^ CNRS UMR 8198 Evo‐Eco‐Paleo Université de Lille ‐ Sciences et Technologies Villeneuve d'Ascq France; ^2^ Montpellier SupAgro UMR 1334 AGAP Montpellier France; ^3^ INRA UR Amélioration des Plantes Marrakech Morocco; ^4^ Laboratoire AgroBiotechL02B005 Faculté des Sciences et Techniques Guéliz University Cadi Ayyad Marrakech Morocco; ^5^ INRA UR Amélioration des Plantes et Conservation des Ressources Phytogénétiques Meknès Morocco; ^6^ Laboratory of Genomics and Plant Breeding DAFNAE ‐ University of Padova Legnaro PD Italy; ^7^ Research Unit for Table Grapes and Wine Growing in Mediterranean Environment CREA Turi BA Italy; ^8^ CNR Institute of Biosciences and Bioresources Perugia Italy; ^9^ INRA/CBNMed UMR 1334 Amélioration Génétique et Adaptation des Plantes (AGAP) Montpellier France

**Keywords:** diallelic self‐incompatibility system, homomorphic system, *Olea europaea* L., Oleaceae, olive diversity, plant mating systems, sporophytic genetic control, trans‐generic conservation of SI functionality

## Abstract

The olive (*Olea europaea* L.) is a typical important perennial crop species for which the genetic determination and even functionality of self‐incompatibility (SI) are still largely unresolved. It is still not known whether SI is under gametophytic or sporophytic genetic control, yet fruit production in orchards depends critically on successful ovule fertilization. We studied the genetic determination of SI in olive in light of recent discoveries in other genera of the Oleaceae family. Using intra‐ and interspecific stigma tests on 89 genotypes representative of species‐wide olive diversity and the compatibility/incompatibility reactions of progeny plants from controlled crosses, we confirmed that *O. europaea* shares the same homomorphic diallelic self‐incompatibility (DSI) system as the one recently identified in *Phillyrea angustifolia* and *Fraxinus ornus*. SI is sporophytic in olive. The incompatibility response differs between the two SI groups in terms of how far pollen tubes grow before growth is arrested within stigma tissues. As a consequence of this DSI system, the chance of cross‐incompatibility between pairs of varieties in an orchard is high (50%) and fruit production may be limited by the availability of compatible pollen. The discovery of the DSI system in *O. europaea* will undoubtedly offer opportunities to optimize fruit production.

## 1 Introduction

Self‐incompatibility (SI), a postpollination prezygotic mechanism preventing self‐fertilization in simultaneous hermaphroditic individuals, is a common feature in flowering plants, occurring in around 40% of angiosperm species (Igic, Lande, & Kohn, 2008). Genetic determination of SI is highly variable, with a single locus or several loci (diallelic or multi‐allelic) and gametophytic or sporophytic control of the pollen SI phenotype (Castric & Vekemans, 2004; De Nettancourt, 1977). Because distinct individuals can share identical SI genotypes, incompatible crosses are not limited to self‐pollination (Bateman, 1952). By limiting compatible matings, SI can cause a direct decrease in seed production and can be an important demographic factor, a phenomenon known as the S‐Allee effect (Leducq et al., 2010; Wagenius, Lonsdorf, & Neuhauser, 2007). This effect is especially important in populations with low genetic diversity (Byers & Meagher, 1992; Vekemans, Schierup, & Christiansen, 1998). Despite its widespread occurrence in angiosperms, the genetic basis of SI has been identified in a limited number of cases, and the underlying molecular mechanism has been shown in only a handful of plant families. These include the Brassicaceae (Kitashiba & Nasrallah, 2014; Tantikanjana, Rizvi, Nasrallah, & Nasrallah, 2009), Papaveraceae (Eaves et al., 2014), Solanaceae, Plantaginaceae, and Rosaceae (Iwano & Takayama, 2012; Sijacic et al., 2004; Williams, Wu, Li, Sun, & Kao, 2015).

Among plant species possessing a functional SI system, crop species are of particular importance because the SI system can interfere with plant production and breeding, representing a major obstacle for constant high yield (Sassa, 2016). A reduction in genetic diversity in commercial varieties may also potentially limit seed and fruit production in field conditions, with adverse economic consequences (Matsumoto, 2014). This issue has stimulated active crossing programs to assess allelic diversity at the SI locus in crop species showing functional SI, such as apple (Broothaerts, 2003), Japanese pear, sweet cherry, apricot (Sassa, 2016; Wünsch & Hormaza, 2004), cabbage (Ockendon, 1974), chicory (Gonthier et al., 2013), and sugarbeet (Larsen 1977). However, despite the obvious interest for breeders to use the SI system to their advantage as part of their breeding programs, proper understanding of the genetic factors and molecular mechanisms involved in SI is lacking for most species and generally technically difficult for breeding companies.

The mechanisms controlling SI are often conserved and shared among species belonging to a given plant family (Allen & Hiscock, 2008; Charlesworth, 1985; Weller, Donoghue, & Charlesworth, 1995). Hence, evolutionary approaches can help to uncover SI mechanisms in crop species based on knowledge of related species. Although SI has evolved independently many times within angiosperms, the rate of evolution of new SI systems is thought to be low, and the occurrence of distinct mechanisms of SI genetic determination within a given family should be rare (Igic et al., 2008).

The olive (*Olea europaea* subsp. *europaea*) is the iconic tree of the Mediterranean area, present in cultivated (var. *europaea*) and wild (var. *sylvestris*) forms (Green, 2002). Despite the economical, ecological, cultural, and social importance of this species, its mating system is still largely controversial and no consensus model has been accepted. The genetic determination and even functionality of SI are still largely controversial. In this species, even the most basic biological details of SI are unresolved and we do not know whether SI is under gametophytic (Ateyyeh, Stosser, & Qrunfleh, 2000) or sporophytic (Breton & Bervillé, 2012; Collani et al., 2012) genetic control. The number of genes involved in the olive SI system and their pattern of linkage and chromosomal location are also unknown.

Cultivars able to produce seed by selfing are thought to exist (Farinelli, Breton, Famiani, & Bervillé, 2015; Wu, Collins, & Sedgley, 2002), but this contention is rarely supported by molecular paternity tests (De la Rosa, James, & Tobutt, 2004; Díaz, Martın, Rallo, Barranco, & De la Rosa, 2006; Díaz, Martín, Rallo, & De la Rosa, 2007; Mookerjee, Guerin, Collins, Ford, & Sedgley, 2005; Seifi, Guerin, Kaiser, & Sedgley, 2012). In a recent study involving paternity assessment of seed progenies from the Koroneiki cultivar (Marchese, Marra, Costa, et al. 2016), it was shown that seeds produced on twigs protected from outcross pollen were derived from self‐fertilization. In addition, open pollination resulted in 11% of the seeds being produced via selfing. Together, these results suggest that SI is indeed functional in this variety, although leaky. Pollination experiments have been performed in several studies to characterize SI at the phenotypic level and identify groups of compatibility among varieties. These studies scored compatibility either at the prezygotic stage, by cytological observations of pollen tube elongation in stigmas, or at the postzygotic stage, by measuring seed production (supported or not by paternity assessment) after application of pollen from donor plants (Breton et al., 2014; Cuevas & Polito, 1997). Contradictory conclusions were drawn in terms of classifying cultivars into SI groups, as well as in terms of the quantitative strength of the incompatibility reaction. Some of these discrepancies may be caused by pollen contamination, likely because *O. europaea* produces a large quantity of pollen typical of most wind‐pollinated species (Ferrara, Camposeo, Palasciano, & Godini, 2007).

Here, we studied the genetic determination of SI in olive in light of recent discoveries in other genera in the same family, Oleaceae. SI has been investigated in *Phillyrea angustifolia* L. and *Fraxinus ornus* L, two androdioecious species in which males and hermaphrodites co‐occur in the same population. Both species share a homomorphic sporophytic diallelic SI (DSI) system (Saumitou‐Laprade et al., 2010; Vernet et al., 2016). Self‐incompatible hermaphroditic individuals belong to one of two homomorphic SI groups: Individuals of a given SI group can only sire seeds on hermaphrodites from the other group, and cross‐pollination between individuals of the same group elicits an incompatibility response (Saumitou‐Laprade et al., 2010). The DSI system has been conserved in both species, and cross‐species pollination tests have demonstrated that the recognition specificities currently segregating in the two species are identical. *P. angustifolia* and *F. ornus* belong to two different subtribes within the Oleaceae (subtribe Oleinae for *P. angustifolia* and subtribe Fraxininae for *F. ornus*). Hence, it has been suggested that this DSI system originated ancestrally within Oleaceae (Vernet et al., 2016) and thus was present in the most recent common ancestor of these two subtribes about 40 million years ago (Mya) (Besnard, de Casas, Christin, & Vargas, 2009). Because *O. europaea* and *P. angustifolia* belong to the same subtribe (Oleinae), we hypothesize that they share the same SI system.

We applied experimental approaches developed for *P. angustifolia* and *F. ornus* (Saumitou‐Laprade et al., 2010; Vernet et al., 2016) to characterize the SI system in *O. europaea*, both phenotypically and genetically. First, we performed controlled pollinations in a full diallel crossing scheme between hermaphroditic individuals used as pollen recipients and pollen donors (including self‐pollination). The objective was to compare the pattern of the incompatibility reactions with those described in *P. angustifolia* and *F. ornus*. Second, we analyzed the pattern of segregation of incompatibility phenotypes among 91 offspring from one single intervarietal cross (De la Rosa et al., 2003). The results were in agreement with a genetic model consisting of a DSI system with two mutually incompatible groups of hermaphrodites. The validity of this genetic model was assessed by performing controlled pollinations with 89 genotypes representative of a significant portion of the olive diversity present in a worldwide collection against two pairs of tester genotypes, each pair being composed of two reciprocally compatible hermaphrodites phenotyped in the first diallel crossing experiment and each assigned to one of the two SI groups. Using pollen from hermaphroditic individuals of *P. angustifolia* and *F. ornus*, we also demonstrated that the same two allelic specificities are shared among the three genera, thereby confirming our hypothesis that they share the same DSI system. The results are presented in light of previous attempts to characterize the SI system in olive. It is worth mentioning that this study focused on the cultivated form of olive (var. *europaea*), analyzing varieties representative of domesticated Mediterranean olive diversity (El Bakkali et al., 2013; Haouane et al., 2011). Our results suggest new avenues for the development of olive orchard management practices to optimize fruit production.

## 2 Materials and methods

### 2.1 Plant material

To avoid any misclassification of varietal clones and to allow their authentication by means of voucher samples, DNA was extracted from each individual tree phenotyped for SI and was genotyped by assaying 15 different polymorphic microsatellite (SSR) marker loci. Hence, we identified each individual tree with a reference DNA sample code, a physical position in the orchard, a genotype reference number corresponding to a specific marker allele combination for the 15 SSR marker loci (Table S1), and an SI phenotype.

In 2013, six genotypes were chosen in Italian orchards and tested for cross‐compatibility using stigma tests in a reciprocal diallel design (Table 1). Because testers had to be used as pollen recipients in future tests, the six genotypes were chosen among those represented by several trees in the experimental stations, at different sites under different agroecological conditions favoring different flowering times for a single genotype, and located as close as possible to laboratory facilities to ensure quick transfer of receptive flowers to the laboratory over the whole study period. From these six genotypes, four were selected to constitute the two pairs of testers used for screening varieties from the olive collections. Receptive flowers sampled from the four chosen tester genotypes were used to phenotype SI in 2013 and 2014. For phenotyping, 118 trees, corresponding to 89 genotypes, were selected from different *ex situ* collections. In particular, 64 trees were kept from the worldwide Olive World Germplasm Bank (OWGB) of INRA Marrakech, at the experimental orchard (Tessaout, Morocco), 45 from the Perugia collection ((43°04′54.4″N; 12°22′56.8E), Italy), and three from the CNR—Institute of Biosciences and Bioresources (CNR‐IBBR) experimental garden (Perugia, Italy), and six were derived from the olive germplasm collection of the Conservatoire Botanique National Méditerranéen (CBNMed) (Porquerolles Island, France) (Table S1). These were used as pollen donors, to define the genetic architecture of SI and to maximize the genetic diversity of sampled *O. europaea* (Belaj et al. 2012; El Bakkali et al., 2013).

**Table 1:** Results from self‐pollination and reciprocal stigma tests performed in a diallel crossing design among six *Olea europaea* genotypes

			Pollen donor					
	SI group		G1			G2		
		DNA database reference	Oit27	Oit26	Oit24	Oit15	Oit30	Oit28
Pollen recipient	G1	Oit27	**SI**	0	0	1	1	1
		Oit26	0	**SI**	0	1	1	1
		Oit24	0	0	**SI**	1	1	1
	G2	Oit15	1	1	1	**SI**	0	0
		Oit30	1	1	1	0	**SI**	0
		Oit28	1	1	1	0	0	**SI**

**Figure 1:**
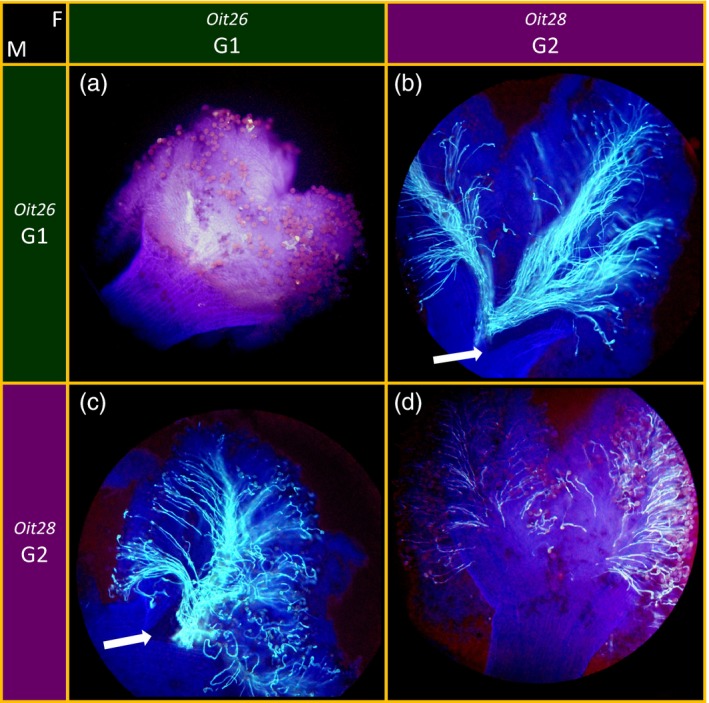
Stigma tests performed to assess self‐incompatibility in *O. europaea*: examples of hermaphrodites Oit26 and Oit28. (a) The pollen of the hermaphrodite *Oit26* does not germinate on its own stigma demonstrating the self‐sterility of this individual; (b) *Oit26* pollen germinates on hermaphrodite *Oit28* attesting to its viability; (c) the stigma from *Oit26* allows germination of *Oit28* pollen attesting to the stigma's functional receptivity when pollinated by compatible pollen; (d) the *Oit26* pollen does not germinate on its own stigma demonstrating the self‐sterility of this individual. Arrows pinpoint the region corresponding to the base of the stigma and entrance of the style. M: genotype used as male pollen donor; F: genotype used as female recipient

SI, self‐incompatibility reaction detected, no or only short pollen tubes observed in stigmatic tissue after self‐pollination; 0, incompatibility reaction, no or only short pollen tubes observed in stigmatic tissue (Figure 1, panel a and d); 1, compatibility reaction, pollen tubes were observed converging through the stigmatic tissue toward the style (see Figure 1 panel b and c). Two incompatible genotypes were assigned to the same incompatibility group (either G1 or G2); two compatible genotypes were assigned to different incompatibility groups. DNA database reference corresponds to voucher specimen accessible in referenced collections (see Table S1).

To verify segregation of the SI phenotype in progeny from an F1 cross, pollen was collected from 91 trees (hereafter called LEDA) growing at the CNR‐IBBR Institute (Table S2) that are the progeny of a controlled cross between Leccino and Dolce Agogia varieties (referenced as Oit27 and Oit15). Their paternity was previously confirmed using RAPD, AFLP, SSR, and RFLP markers (De la Rosa et al., 2003).

### 2.2 Genotyping of the sampled trees with microsatellite markers

To genotype sampled trees, total DNA was extracted from 100 mg of fresh leaf tissue by GeneElute Plant Genomic DNA Miniprep Kit (Sigma‐Aldrich), following manufacturer's instructions, and then quantified by a Nanodrop spectrophotometer. Genotype identification was performed by analyzing 15 informative nuclear SSR markers (Baldoni et al., 2009; El Bakkali et al., 2013). PCR products were separated using an automatic capillary sequencer (ABI 3130 Genetic Analyzer, Applied Biosystems), and electropherograms were then investigated for allele composition across marker loci using GenMapper 3.7 software (Applied Biosystems).

To verify the genetic representation of the selected sample set, SSR data obtained on the 118 trees (Table S1) were compared to a collection of 342 genotypes: the 309 olive genotypes present in the OWGB collection (El Bakkali et al., 2013) together with the 33 genotypes sampled in Italian (27) and French (6) collections not present in the OWGB.

### 2.3 Assessments of the compatibility/incompatibility reactions

#### 2.3.1 Incompatibility tests

To ensure that receptive stigmas were free of contaminant pollen, branches about 40–50 cm long and bearing several flower buds were bagged on tester recipients at least one week before flowers opened and stigmas became receptive (using two PBS3d/50 bags, an outer bag enclosing an inner bag, each of size 16 × 50 × 16 cm; PBS International, Scarborough, UK). A 10 × 25 cm PVC window allowed us to monitor flowers or treat them without opening the bags. For prezygotic stigma tests, twigs were collected when flowers were mature (i.e., when 5%–10% of flowers present on a twig were open). When performing postzygotic tests by controlled crosses and scoring of produced seeds, to prevent pollen contamination during pollination, the outer bag was removed and the inner bag was pierced with a needle to inject pollen with a spray gun, the needle hole was carefully taped immediately after spraying, and the outer bag was put back in place. To ensure continuous pollen availability, freshly collected pollen was stored at −80°C (Vernet et al., 2016) until it was applied to recipient stigmas; this procedure also allowed us to collect pollen on the latest flowering tree in 2013 for use in phenotyping on stigmas in 2014.

#### 2.3.2 Stigma test

We scored cross‐compatibility following the protocol in Vernet et al. (2016). Under these conditions, stigmas treated with pollen were fixed 16 hr after pollination, then stained with aniline blue, and observed under a UV fluorescent microscope, which allowed us to distinguish pollen grains and pollen tubes from maternal tissues (Figure 1). When the pollen recipient and the pollen donor are compatible, several pollen tubes converge through the stigmatic tissue toward the style until the base of the stigma and entrance of the style (Figure 1 panels b and c). The absence of pollen tubes or the presence of only short pollen tubes growing within the stigma but never reaching the style was used as the criteria to score incompatibility (Figure 1 panels a and d). Given the risk of contamination, a single pollen tube growing in the stigmatic tissue was never considered a reliable criterion to determine compatibility. Three replicate flowers were pollinated for each cross.

#### 2.3.3 Interspecific stigma tests between *O. europaea*,* P. angustifolia*, and *F. ornus*

To (i) substantiate our conclusions on the occurrence of DSI in *O. europaea*, (ii) determine whether SI recognition specificities have remained stable in the hermaphrodite lineage of the *Olea* genus as its divergence from the lineage containing the androdioecious species *P. angustifolia*, and (iii) take advantage of recent knowledge about the genetic architecture of SI in *P. angustifolia* (Billiard et al., 2015), we performed interspecific stigma tests using *P. angustifolia* and *F. ornus* hermaphrodites assigned to the two SI groups in previous studies (called G1 and G2) (Saumitou‐Laprade et al., 2010; Vernet et al., 2016). Stigma tests were performed using the two pairs of *O. europaea* testers (Oit27/Oit15 and Oit30/Oit26) as recipients, with frozen pollen from *P. angustifolia* (Pa‐01C.02 [G1] and Pa‐06G.15 [G2]) and *F. ornus* (Fo‐A17 [G1] and Fo‐G_1999_‐48 [G2]) belonging to the G1 and G2 SI groups.

We performed stigma tests by depositing pollen from one test sample on stigmas of a pair of cross‐compatible testers (i.e., belonging to two different SI groups). Under the hypothesis that *O. europaea* exhibits DSI, we expected pollen from every sample to be compatible with one of the two tester lines (thereby confirming pollen viability) and incompatible with the other; indeed, cases in which pollen would be compatible with both tester lines would indicate either the presence of a third SI group (different from these represented by the tester recipients) or a nonfunctional SI genotype. Cases in which tested pollen was negative with both reference recipients were likely caused by either low pollen viability or low stigma receptivity. Hence, pollinations were repeated until compatibility was observed on at least one pollen recipient.

### 2.4 Postzygotic validation of SI group assignment

To validate the compatibility versus incompatibility status assessed between pairs of genotypes, according to pollen tube behavior in the stigma tests, we carried out additional phenotypic assessments based on seed production after controlled pollination. We followed the protocol established and validated for *P. angustifolia* (Saumitou‐Laprade et al., 2010). Each tested genotype was used as a pollen recipient and treated as follows: Two 40–50 cm long branches per tree carrying numerous inflorescences were selected a week before the opening of the first flowers and carefully protected in two bags each. One inflorescence was pollinated with pollen collected from pollen donors belonging respectively to the G1 and G2 incompatibility groups (Oit27 and Oit15, respectively, see Results and Table 1). For each cross, pollination was repeated three times over a period of 8 days, beginning when the first flowers opened (between late May and mid‐June 2014, depending on the flowering stage of the recipient). Isolation bags were removed several days after the end of flowering (on July 10) and replaced by net bags to prevent loss of fruit during ripening. Finally, fruits were collected and counted in mid‐October, and to confirm paternity, genomic DNA was extracted from fruit embryos (Díaz et al., 2007) and from leaves of the parents. Parents and offspring were genotyped using 10 highly polymorphic microsatellite markers (Table S3) having high exclusion probability (El Bakkali et al., 2013). Paternity assignments were calculated with Cervus 3.0.3 (Table S4).

### 2.5 Genetic assignment of the trees phenotyped for SI group and assessment of genetic diversity

To determine how our sampling represented the genetic diversity and the geographic structure of the Mediterranean olive tree, SSR alleles were scored in a single analysis (Tables S1 and S5) and combined with previous data obtained from the complete OWGB collection (El Bakkali et al., 2013).

The number of alleles per locus (Na), the observed (Ho) heterozygosity and expected (He) heterozygosity (Nei, 1987) were estimated using the Excel Microsatellite Toolkit v3.1 (Park, 2001). Principal coordinate analysis (PCoA), implemented in the DARWIN v.5.0.137 program (Perrier, Flori, & Bonnot, 2003), was carried out using a simple matching coefficient. To identify the genetic structure within the studied samples, in comparison with the Mediterranean olive germplasm, a model‐based Bayesian clustering implemented in the program Structure ver. 2.2 (Pritchard, Stephens, & Donnelly, 2000) was used. Bayesian analysis was run under the admixture model for 1,000,000 generations after a burn‐in period of 200,000, assuming correlation among allele frequencies. Analyses were run for values of *K* between one and six clusters with 10 iterations for each value. Validation of the most likely number of *K* clusters was performed using the Δ*K* statistics developed by Evanno, Regnaut, and Goudet (2005) with the R program, and the similarity index between 10 replicates for the same *K* clusters (H’) was calculated using CLUMPP 1.1 (Greedy algorithm; (Jakobsson & Rosenberg, 2007)). For each selected *K* value, each accession was assigned to its respective cluster with a posterior membership coefficient (*Q* > 0.8).

We tested whether the allelic diversity observed in the 89 genotypes representing a subsample of the core collection was significantly lower than that of the overall OWGB collection using a Mann–Whitney *U*‐test (*p‐*value > .01 one‐tailed test) after standardization of the dataset using the rarefaction method according to ADZE (Szpiech, Jakobsson, & Rosenberg, 2008).

### 2.6 Statistical analysis of pollen tube length scored in incompatible crosses

**Figure 2:**
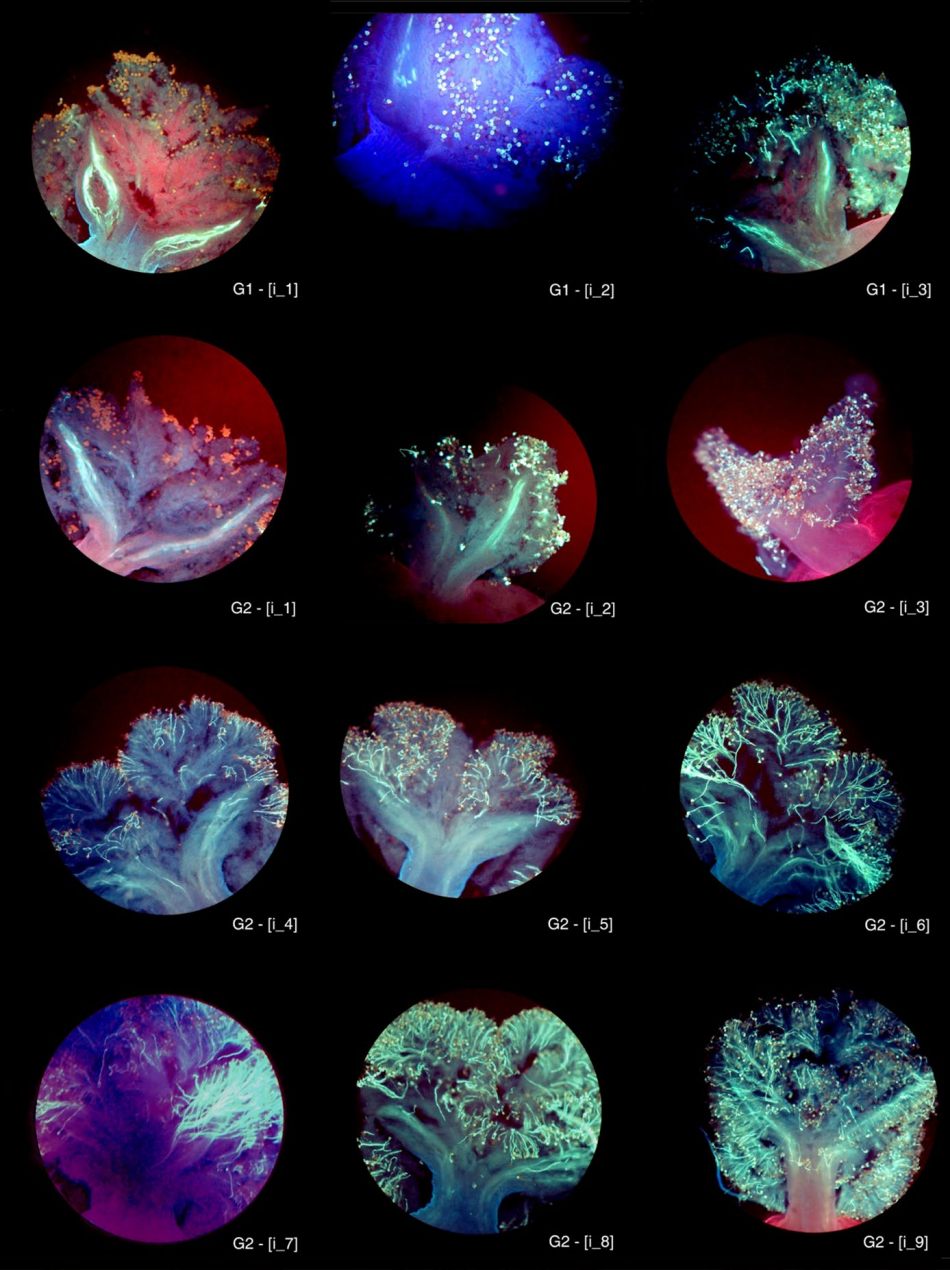
Classes of incompatibility phenotypes observed within self‐incompatibility groups according to pollen (donor × recipient) interactions. On stigmas belonging to the G1 group, pollen tube length after growth was arrested was homogenous: from null to low (see the cases G1: [i_1] to [i_3]). On stigmas of G2 groups, pollen tube length after the arrest of growth varied widely among (donor/recipient) pairs: from null to high (see the cases G2: [i_1] to [i_9])

The specific length of pollen tubes within stigmatic tissue was measured for a given set of incompatible reactions (i.e., the growth that occurred prior to the arrest of further growth). Based on this growth, we defined nine discrete phenotypic classes, from i_1 to i_9 (Figure 2). Because an incompatible response scored in the highest phenotypic classes (i.e., longer pollen tubes, see [i_7], [i_8], and [i_9]) could be mistaken for a compatible response, we applied generalized linear model (GLM) analyses to the phenotypic data. A subset of 86 pollen donors was crossed with the four *O. europaea* testers involved in the stigma test described above (Table S6). For each pollen donor, we observed four crosses (two compatible and two incompatible), and for each cross, we photographed pollen tubes growing down stigmatic tissue and styles in three different flowers. The images were randomly labeled and observed four times independently, providing four reads for assignment to a phenotypic class (i.e., 12 independent scores for each cross).

First, we tested the effect of the SI group, replicate scoring, pollinator genotype, recipient genotype, and individual flowers on the SI phenotypic response (i.e., the length of pollen tube growth before growth arrest within stigmatic tissue in incompatible crosses, scored among nine phenotypic classes by the experimenter). We then used generalized linear mixed models (GLMM) on the categorized phenotype of the SI response to test the following: (i) whether the phenotype scoring based on digital images was repeatable, (ii) whether the phenotype was consistent among replicates of the same pollination test, (iii) whether the SI groups showed a different SI response, and (iv) whether the genotype of the recipient had an effect on the SI response. We considered the factors “flower read,” “pollinator genotype,” and “flower” as random effects, and “recipient genotype” and “SI group” as fixed effects. The SI response was the dependent variable and followed a Poisson distribution. To test whether a random or fixed factor had a significant effect, we performed a likelihood ratio test of nested models, using the package lme4 in R (Bates et al., 2014).

## 3 Results

### 3.1 Phenotypic characterization of self‐incompatibility in *O. europaea*

In 2013, six accessions (Oit27, Oit26, Oit24, Oit15, Oit30, and Oit28) corresponding to six different genotypes (Table S1) were used as both pollen recipients and pollen donors, in a reciprocal diallel design, for the stigma tests (Table 1). Self‐fertilization was tested on the six genotypes, and no pollen tube successfully reached the style in any of the observed pistils, confirming strong SI reactions (Table 1). However, the length of pollen tubes within the stigmatic tissues varied between genotypes. Pollen tubes did not grow at all, or their growth stopped very early in the first layer of the stigma cells in the Oit26 genotype (Figure 1, panel a). In comparison, arrest of pollen tube growth did not occur until the pollen tubes had reached the deeper layers of the stigma cells, in the Oit28 genotype (Figure 1, panel d). However, even in this case, the pollen tubes stopped before reaching the transmitting tissue of the style.

For the intergenotype pollination tests, pollen tube/stigma interactions suggested the existence of SI reactions for each of the six different individual trees when crossed with specific partners (Table 1). An incompatibility phenotype similar to the Oit26 self‐fertilization reaction (Figure 1, panel a: no or very short pollen tubes detected) was observed in the stigmatic tissues from Oit27, Oit26, and Oit24 when their pistils were pollinated by one another. The viability of their pollen and receptivity of their stigmas were verified in compatible crosses with Oit15, Oit30, and Oit28. A phenotype similar to the SI reaction observed with Oit28 (Figure 1, panel d: pollen tubes of variable length never reaching the style) was observed in the stigmas from Oit15, Oit30, and Oit28 when pollinated by one another. Here again, pollen viability and stigmatic receptivity of the same three individuals were checked in compatible crosses with Oit27, Oit26, and Oit24. We concluded that trees Oit27, Oit26, and Oit24 are incompatible with each other and belong to a single SI group, whereas trees Oit15, Oit30, and Oit28 belong to a different SI group. These results suggest that *O. europaea* individuals can be classified into at least two groups of SI, with incompatibility reactions between individuals belonging to the same group and compatible reactions between individuals belonging to different groups.

### 3.2 The two *O. europaea* SI groups are functionally homologous to those of *P. angustifolia* and *F. ornus*

**Figure 3:**
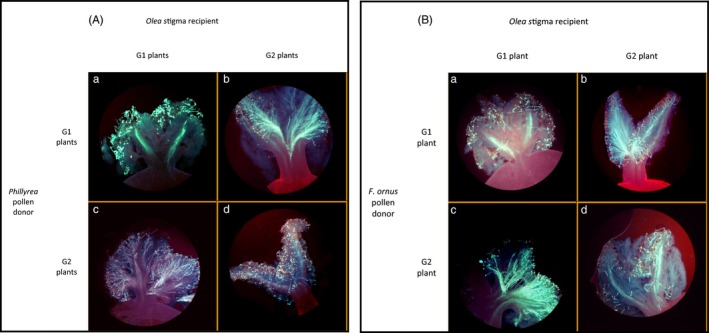
Trans‐generic conservation of the self‐incompatibility reaction between *Olea europaea* and two other Oleaceae species: (A) *Phillyrea angustifolia* and (B) *Fraxinus ornus*. In the photographs presented, stigma from *O. europaea* is pollinated by *P. angustifolia* and *F. ornus* pollen. (a) Incompatibility reaction between stigma of *Oit26* and G1 pollen; (b) compatibility reaction between stigma of *Oit15* and G1 pollen; (c) compatibility reaction between stigma of *Oit26* and G2 pollen; (d) incompatibility reaction between stigma of *Oit15* and G2 pollen

Nonambiguous and repeatable incompatibility phenotypes were observed when *P. angustifolia* and *F. ornus* G1 pollen was deposited on stigmas from Oit26 and Oit27 (Figure 3A,B, panel a), whereas compatibility phenotypes were scored on stigmas from Oit15 and Oit30 (Figure 3A,B, panel b). This demonstrated the capacity of trans‐generic pollen to germinate and elicit both incompatible and compatible responses on *O. europaea* stigmas. Similarly, incompatibility phenotypes were scored on stigmas from Oit15 and Oit30 (Figure 3A,B, panel d), and compatibility phenotypes were observed with *P. angustifolia* and *F. ornus* G2 pollen on stigmas from Oit26 and Oit27 (Figure 3A,B, panel c). Therefore, we concluded that the SI system of *O. europaea* is functionally homologous to the DSI system previously reported for *P. angustifolia* and *F. ornus* (Saumitou‐Laprade et al., 2010; Vernet et al., 2016). We assigned the Oit26 and Oit27 genotypes, and all their incompatible mates, to the G1 SI group, and the Oit15 and Oit30 genotypes, and all their incompatible mates, to the G2 SI group.

### 3.3 Segregation of the self‐incompatibility phenotypes in a controlled cross

Among the 91 LEDA full‐sib trees that flowered in 2013 and/or in 2014, 41 were incompatible with G1 recipients and compatible with G2, indicating that they belong to the G1 compatibility group, and 50 were incompatible with G2 recipients and compatible with G1, indicating that they belong to G2. No offspring appeared compatible or incompatible with both groups of recipients in any of the tests (Table 2). The observed data agree with a genetic model assuming a 1:1 segregation of the two phenotypic groups (Chi² test statistic = 0.345, *df* = 1, ns).

**Table 2:** Self‐incompatibility phenotyping of the 91 LEDA F1 trees from the (Oit64 × Oit27) controlled cross

SI group	[G1] *S1S2* a	[G2] *S1S1* a	[Other] *SxSy*
	Incompatible with G1 and compatible with G2	Incompatible with G2 and compatible with G1	Compatible with G1 and G2
Total	41	50	0

Three types of behavior were scored. [G1], individual incompatible with G1 testers and compatible with G2 testers; [G2], individual incompatible with G2 testers and compatible with G1 testers; [Other], individual compatible with G1 and G2 testers and therefore belonging to a SI group different from G1 and G2 The S‐locus segregates as a single locus with two alleles *S1* and *S2* (with *S2* dominant over *S1*) (Chi² test = 0.345, *df* = 1).

a Expected genotype deduced from genetic analyses in *P. angustifolia* (Billiard et al., 2015).

### 3.4 Two and only two self‐incompatibility groups detected in *O. europaea*

The 118 sampled trees from the four germplasm collections (OWGB, Perugia collection, CNR‐IBRR, and CBNMed) represented 89 distinct genotypes (20 genotypes were represented by more than one clonal replicate, Table S1). We performed a total of 1,500 pollination tests, which allowed us to determine the SI phenotype of each individual tree with a mean of 2.6 replicates per tested genotype. All replicates were fully concordant (Fig. S1), demonstrating the robustness and reliability of the stigma tests performed. Among the 89 genotypes, 42 genotypes were incompatible with G1 recipients and compatible with G2, indicating that they belong to G1, and 47 genotypes were incompatible with G2 recipients and compatible with G1, revealing that they belong to G2. None of the genotypes were either compatible or incompatible with both groups of recipients, proving that two and only two SI groups were present in our extended sample (Table 3).

**Table 3:** Result of stigma tests performed with 89 *O. europaea* genotypes tested for compatibility and incompatibility with two pairs of pollen recipients used as testers

SI group	[G1]	[G2]	[Other]
	Incompatible with G1 and compatible with G2	Incompatible with G2 and compatible with G1	Compatible with G1 and G2
Total	42	47	0

Three types of behavior were scored (see Table 2 caption). The cultivars tested belong either to G1 or to G2, and none belong to a hypothetical third incompatibility group. In the sample tested, we detected only two incompatibility groups.

### 3.5 Population genetic assessment of the sampling and representativeness of olive diversity

The samples we phenotyped for SI represented 89 distinct genotypes. We were highly conservative in our genotype identification: We grouped genotypes defined by a specific allele combination at 15 loci that differed by only one allele or two alleles into a single genotype considering the possibility that their differences derived from somatic mutations that occurred within old clones (Haouane et al., 2011). Within the 89 genotypes, we detected 179 alleles over the 245 scored on the collection of 342 genotypes (Table S5‐A). Hence, our sample captured 73% of the total allelic diversity observed in the collection of 342 genotypes. To check for the representativeness of olive diversity in our subsample of 89 genotypes, we compared allelic richness in the subsample with that of the collection of 342 genotypes after correction for difference in sample sizes based on the rarefaction method (see Table S5‐B). Allelic richness of the two sample sets was not significantly different (Mann–Whitney *U*‐test at *p *≤* *.01 using one‐tailed test; U = 89, *P*‐value = .171; Table S5‐B). Furthermore, we noted similar expected heterozygosity values (He = 0.745 in the 89 genotypes and He = 0.749 in the 342 genotypes collection; Table S5‐A).

Most of the 89 genotypes phenotyped for SI were classified into one of the three western, central, and eastern Mediterranean clusters detected in previous studies (El Bakkali et al., 2013; Haouane et al., 2011), with a slight underrepresentation of the eastern gene pool (Table S5‐C). This conclusion was further confirmed by their position on the first two axes of the principal coordinate analysis (PCoA, Fig. S3). Overall, despite the limited number of eastern olive trees, the 89 genotypes were distributed among the three Mediterranean gene pools, indicating they were a fair representation of domesticated olive diversity.

### 3.6 The incompatibility response differs between the two self‐incompatibility groups

When we analyzed variation in pollen tube lengths, we found no significant variation among replicate observations of the same flower and among flowers of a single individual when pollinated with incompatible pollen, indicating consistent incompatibility reactions. However, the SI group of the pollen recipient had a significant effect (*p‐*value <.0001) on the distance that incompatible pollen tubes were able to grown within the stigma: Plants belonging to G1 showed an SI phenotype that fell in classes of low value (short pollen tubes), whereas plants belonging to G2 showed phenotypes that can fell in a wider panel of values (from short to long pollen tubes). The incompatibility reaction between G1 individuals seemed to occur almost immediately after pollen landed on the stigma as either pollen grains did not germinate or pollen tube growth stopped shortly after germination. In contrast, the incompatibility reaction between G2 individuals seems to occur later: Pollen grains germinated and pollen tubes grew into the stigmatic tissue before their growth was arrested.

Our analyses also revealed a significant effect of the recipient genotype on the score value within each SI group (*p‐*value <.001 for both G1 and G2). Within G2, Oit30 showed a higher score than Oit15, and within G1, Oit27 showed a higher score than Oit26. This suggests consistent differences among genotypes in the timing of the SI response (early or late), whose functional significance remains to be determined.

### 3.7 The SI assignment based on prezygotic stigma test validated by postzygotic genotyping

We verified at the postzygotic stage whether cases of incompatibility in which pollen tubes were able to germinate and grow substantial distances in the stigma (therefore the most ambiguous cases because of their relative similarity to compatible phenotypes) were cases in which fertilization was not achieved. The functional incompatibility of 10 different genotypes, belonging to SI group G2 and which scored in the highest phenotypic classes [i_7], [i_8], and [i_9] (Figure 2), was assessed at the postzygotic stage through progeny analysis (Table S4), as well as counts of the number of seeds produced (Table 4). All 99 progeny from crosses between putatively compatible mates (assigned based on the prezygotic stigma test), had genotypes compatible with both parents (Table S3). This confirms that the stigma test is reliable and suggests that our experimental design prevents pollen contamination. In contrast, the number of seeds collected following the 10 pollinations between parents belonging to the same SI group was extremely low (no seed produced in seven crosses and 2, 2, and 10 seeds in the three remaining crosses, respectively). In addition, none of the seeds harvested in these three crosses had a genotype that was consistent with its putative father. Again, these results confirm that the stigma test is a reliable procedure to predict which incompatibility group a plant belongs to, even in those cases in which some pollen tube growth occurs within the stigma. Interestingly, the few seeds obtained were all attributed to selfing.

**Table 4:** Number of seeds collected on G2 genotypes after controlled compatible and incompatible crosses performed in June 2014 and verified by paternity testing

[G2] Genotypes used as recipient	Pollen donors			
	[G1]: Oit27		[G2]: Oit15	
	Seeds produced	Paternity confirmed/tested	Seeds produced	Paternity confirmed/tested
LEDA_222	27	NA	0	–
LEDA_262	24	NA	0	–
LEDA_282	102	20/20	0	–
LEDA_301	98	20/20	0	–
Oit28	15	NA	2	0/2^a^
Oit03	30	10/10	0	–
Oit55	16	12/12	10	0/10^a^
Oit57	17	17/17	2	0/2^a^
Oit36	25	10/10	0	–
Oit22	40	10/10	0	–

a Selfing cannot be excluded with the 10 microsatellite markers used (see Tables S3 for genotyping results and S4 for estimation of exclusion probability based on markers and calculated using Cervus ver. 3.0.3.); NA, fruits not collected.

## 4 Discussion

### 4.1 Confirmation that the three genera *Olea*,* Phillyrea*, and *Fraxinus* share the same self‐incompatibility system

The evolution of new SI systems in plants is thought to be a rare phenomenon, which is in agreement with the general observation that SI mechanisms are generally shared among species that exhibit SI within a given plant family (Allen & Hiscock, 2008; Charlesworth, 1985; Igic et al., 2008; Weller et al., 1995) and that losses of SI within a clade are much more common than gains (Igic, Bohs, & Kohn, 2006). As expected based on these arguments, we confirmed in *O. europaea* the occurrence of the same DSI discovered in *P. angustifolia* and *F. ornus* (Vernet et al., 2016)*,* two androdioecious species that belong to two phylogenetic branches of the same family that diverged from each other more than 40 Mya (Besnard et al., 2009). While SI systems are often trans‐generic, long‐term stability of homomorphic DSI—that is the presence of only two alleles over a long time—is unexpected for two reasons. First, SI systems are susceptible to the rapid invasion of new incompatibility alleles, as a consequence of the strong frequency‐dependent advantage of rare mating phenotypes (Wright, 1939). Gervais et al. (2011) showed that in a model where new alleles arise through self‐compatible intermediates, selection for allelic diversification is inversely related to the number of segregating S‐alleles*,* that is, more active diversification with a low number of alleles. Second, in hermaphroditic species, self‐compatible mutants are expected to invade a homomorphic DSI population regardless of the extent of inbreeding depression (Charlesworth & Charlesworth, 1979). The stability of DSI was recently explained in the case of androdioecy with a theoretical model (Van de Paer, Saumitou‐Laprade, Vernet, & Billiard, 2015), showing that androdioecy and DSI help maintain each other. DSI facilitates the maintenance of males (Billiard et al., 2015; Husse, Billiard, Lepart, Vernet, & Saumitou‐Laprade, 2013; Pannell & Korbecka, 2010), and the full compatibility of males hinders the invasion of self‐compatible mutants (Van de Paer et al., 2015).

The situation is quite different for *O. europaea*. The species belongs to the subgenus *Olea* which contains only hermaphrodite species and has diverged more than 30 Mya from the lineage containing androdioecious taxa such as *Osmanthus* and *Phillyrea* (Besnard et al., 2009). The evolutionary causes of the maintenance of DSI over 30 My remain to be identified. Molecular characterization of the SI locus is a promising avenue of research to resolve issues related to the origin and maintenance of homomorphic DSI, because the simplest explanation is that the genetic architecture of the system does not allow the generation of additional SI phenotypes (e.g., a third SI allele). Molecular characterization will be facilitated by the trans‐generic functionality of DSI that we observed among the *P. angustifolia, O. europaea*, and *F. ornus* species.

### 4.2 Self‐incompatibility in *O. europaea* is sporophytic

Our results are consistent with determination of SI on *O. europaea* by a single S‐locus with only two alleles present in all cultivated forms of the species and demonstrate the sporophytic nature of this SI. First, the 1:1 proportion of the two parental SI groups in the controlled cross progeny excludes the possibility of gametophytic genetic control of self‐incompatibility (GSI) (Bateman, 1952) in *O. europaea*. Second, with GSI, the incompatibility gene at the S‐locus is expressed in the haploid pollen grains and interacts with the diploid tissue of the stigma. To be functional, a GSI system requires strict codominance between S‐alleles in the pistil to avoid compatibility of heterozygous individuals with half of their own (self) pollen and a minimum of three alleles that define a minimum of three incompatibility groups (Hiscock & McInnis, 2003). In contrast, in the case of sporophytic genetic control of self‐incompatibility (SSI), the incompatibility gene is expressed before meiosis in the diploid sporophytic tissue, and incompatibility arises with only two alleles, with a complete dominance of one allele over the other (see reviews by (Hiscock & McInnis, 2003; Billiard, Castric, & Vekemans, 2007). In our recent genetic study performed with *P. angustifolia* (Billiard et al., 2015), we showed a SI system governed by an S‐locus with two alleles, *S2* and *S1* (with *S2* dominant over *S1*), which produced the two incompatibility groups G1 and G2 (Saumitou‐Laprade et al., 2010), and with *S1S2* corresponding to G1 and *S1S1* to G2 (Billiard et al., 2015).

The gametophytic versus sporophytic nature of the SI system in *O. europaea* has been questioned for a long time in the literature, using indirect arguments, and several studies on the SI of olive cultivars have resulted in variable and conflicting results (for a review see Seifi, Guerin, Kaiser, & Sedgley, 2015). Features revealed by histological investigations, such as binucleate pollen and wet papillae stigma or a solid style and a large number of pollen grains germinating on the stigma surface, were reminiscent of species with GSI (De Nettancourt, 1997), whereas a dry papillae stigma was also reported in Oleaceae (Heslop‐Harrison & Shivanna, 1977). Additional arguments based on the observation of pollen tube growth in incompatible crosses were in favor of GSI: The way pollen tubes halted in the proximal area of the style was interpreted as the intervention of programmed cell death, a frequent feature of GSI. Other arguments based on histochemical location of key enzyme activities involved in GSI were also reported in olive tree (Serrano & Olmedilla, 2012). Moreover, transcriptome analyses have been performed to screen for conserved transcripts typical of GSI in other plant species (e.g., S‐ribonuclease transcripts such as in Solanaceae; (McClure, 2006)) or SSI (e.g., S‐receptor kinase transcripts, such as in Brassicaceae; (Takasaki et al., 2000)). Transcripts similar to male and female SSI determinants of Brassicaceae were identified in olive (Alagna et al., 2016; Collani et al., 2010, 2012); however, there is no evidence of their functionality in SI reaction.

Here, we demonstrated that one of these numerous indirect arguments for assessing the gametophytic/sporophytic status of SI was wrong: For one SI group, the incompatibility reaction takes place at the stigma, whereas for the other SI group, the incompatibility reaction occurs later, sometimes at the entrance of the style. This feature may explain some of the past difficulty in identifying the gametophytic or sporophytic nature of the incompatibility system in *O. europaea*.

### 4.3 Within‐group incompatibility is stricter than within‐individual self‐incompatibility in *O. europaea*

One surprising observation from our experiments is the production of a small number of selfed seeds by G2 individuals following pollination with incompatible outcross pollen. This is the only indication of a partial breakdown of SI in the face of an otherwise very strong SI reaction. Why self‐pollination seems to be promoted in the presence of incompatible outcross pollen remains to be determined. This feature is unexpected because in SI systems, the incompatibility phenotype of a pollen grain should only depend on the pollen parent genotype at the S‐locus, which is shared among individuals from the same SI group. This result may indicate that, in olive or, at least, in some olive genotypes, the incompatibility reaction may be stronger with outcross pollen from the same group than with self‐pollen. It is also possible that this observation results from the larger amount of self‐pollen deposited on stigmas through autonomous self‐pollination, compared with the outcross pollen transferred experimentally.

### 4.4 *Olea europaea* is a true self‐incompatible species in which some genotypes can produce seeds by selfing

All genotypes tested for SI in the present study were classified as self‐incompatible according to the criteria of our stigma test, and all belong to one of the two SI groups identified in the species. These statements confirm that *O. europaea* is a true self‐incompatible species. They are in agreement with conclusions of studies that tested SI in *O. europaea* at the postzygotic level, by measuring seed production after controlled crosses or open pollination, together with paternity analysis of the progeny (De la Rosa et al., 2004; Díaz et al., 2006; Marchese, Marra, Caruso, et al. 2016; Marchese, Marra, Costa, et al., 2016; Mookerjee et al., 2005). Just as in our study, many studies observed seeds produced by selfing either from controlled crosses with pollen from incompatible genotypes (see Oit28, Oit55, and Oit57 in Tables S3 and S4) or in controlled selfing (Farinelli et al., 2015) or open pollination (Marchese, Marra, Costa, et al., 2016). The self‐incompatible status of a species does not exclude the possibility that the incompatibility reaction may be broken for self‐pollen in some genotypes. The underlying mechanism allowing this remains to be studied. The occurrence of a low rate of selfing in individual plants with an active SI system is commonly reported and is referred to as pseudo‐self‐compatibility or leaky self‐incompatibility. Leaky SI is generally thought to be a consequence of environmental factors interfering with the SI reaction or to the action of modifier genes (Busch & Schoen, 2008; Levin, 1996).

The leaky SI observed in olive has provided material for genetic mapping and sequencing (Marchese, Marra, Caruso, et al. 2016) and allows an opportunity to measure inbreeding depression. For example, in the wild relative *P. angustifolia*, 2% of 2,000 surveyed seedlings produced from controlled crosses were found to have been selfed (Billiard et al., 2015). Notably, none of these selfed seedlings ever flowered (unpublished results). In addition, leaky SI in olive might explain the gradient of results that have until now masked the real self‐incompatibility system.

## 5 Conclusion: additional evolutionary applications

The level of interindividual incompatibility that we observed in our stigma test was very high: On average, half of the pairs of genetically distinct trees from the sampled collections were mutually incompatible. Similarly, most of the studies checking for compatibility within and among olive varieties, when using seed production and paternity analyses, detected numerous cases of cross‐incompatibility (De la Rosa et al., 2003; Díaz et al., 2006; Mookerjee et al., 2005; Wu et al., 2002). In contrast, studies in orchards or crops of other domesticated species with SI, under either GSI (e.g.*, Prunus*,* Malus*,* Pyrus*,* Amygdalus*) or SSI (e.g., *Brassica, Cichorium*), show high numbers of S‐alleles and therefore high levels of cross‐compatibility within or between cultivars (Dreesen et al., 2010; Ockendon, 1982; Wünsch & Hormaza, 2004). In the olive, the low number of elite varieties that co‐occur in an orchard, together with the 50% chance of cross‐incompatibility between pairs of varieties according to its DSI system, may limit fruit production. Limitation of the availability of compatible pollen, a phenomenon described as the S‐Allee effect, occurs in wild populations of SI species with low S‐allele diversity (Leducq et al., 2010; Wagenius et al., 2007). Small isolated populations or populations that have experienced a recent genetic bottleneck may have limited allelic diversity at the S‐locus, leading to an increase in the probability of interindividual incompatibility, which in turn causes a reduction in seed production (Byers & Meagher, 1992; Vekemans et al., 1998).

The discovery of the DSI system in *O. europaea* will undoubtedly offer opportunities to optimize fruit production. First, it helps to understand the heretofore unexplained beneficial effect of ancestral practices that encourage the planting of a minimum number of varieties to ensure satisfactory olive production. Second, easy‐to‐use methods should be developed to determine the SI phenotype of each cultivated variety of olive to help guide the choice of varieties to be assembled in a given orchard, especially in nontraditional olive growing areas. Finally, ecological models can be developed to address the question of the optimal number of varieties to be introduced to ensure, effective pollination in an orchard, regardless of climate. Clearly, mono‐varietal orchards must be avoided. In addition to the SI phenotype, the choice of varieties should take into account other important parameters such as flowering phenology, the direction of wind during the flowering period, and the relative positions of the different varieties within the orchard.

In the present study, we chose to present varieties through their reference genotype and not through their variety name, to assess the strict association between genotype and SI phenotype. Previous studies suggested possible discrepancies between varietal names and genotypes (El Bakkali et al., 2013; Haouane et al., 2011; Trujillo et al., 2014), and during our study, we observed different names associated with a single genotype (Table S1) as well as different genotypes associated with a single variety name; indeed, in more than 20% of cases, the genotypes associated with the same name were different in the Italian and OWGB collections (data not presented). Therefore, there is no strict association expected between variety name and SI phenotype. Therefore, each genotype of interest for olive producers needs to be assigned to one of the two SI groups. This will require characterizing these genotypes for their SI phenotype using the stigma test in rigorous conditions. Lastly, an effort should be devoted to identifying molecular markers with strong linkage with the S‐locus to provide an easy‐to‐use diagnostic molecular assay for genotyping trees at the S‐locus. We are confident that the evolutionary conservation of the functionality of the DSI among the *Olea*,* Phillyrea*, and *Fraxinus* genera will be an asset for accomplishing this task, through genomic and transcriptomic comparative analyses of the two groups within and among these three genera.

## Data and material sharing

All relevant data are within the paper and its Supporting Information files.

## Conflict of interest

The authors declare no conflict of interest.

## Authors’ contributions

All authors contributed significantly to the work presented in this manuscript. P.S.‐L. and P.V. jointly designed and carried the sampling design and phenotyping strategies with B.K. and L.B.. P.S.‐L. and P.V. performed phenotyping and crosses together with F.A., R.M., S.P., and M.R.. B.K. organized the collect of pollen in the OWGB collection in Marrakech (Morocco) and at the CBNMed in Porquerolles (France) together with A. Mh and A. Mo. L.B. organized the collect of pollen in the Perugia collection (Italy) and in the CNR‐IBBR collections (including the LEDA F1 progeny). L. E. and N.G.M.C. performed DNA extraction and genotyping. B.K. and A.E.B. performed population genetic structure analyses. R.M. performed paternity analyses. S.B. performed statistical analyses of the variation in pollen tube lengths. S.G. created bioinformatics tools allowing management, comparison, and sharing among partners of the thousands of pictures produced for the SI phenotyping. G.B. provided expertise on olive SI and strongly contributed to initiate the project. X.V. provided expertise on the SI and population genetics analysis. P.S.‐L., P.V., and X.V. wrote the paper.
