# Supplementary material for: Elucidation of the genetic architecture of self‐incompatibility in olive: Evolutionary consequences and perspectives for orchard management
Source: Evol Appl. 2017 May 20;10(9):867–80. doi: 10.1111/eva.12457 (PMC5680433; doi:10.1111/eva.12457)

Fig. S1. Number of genotypes (haplotype absolute frequencies) in classes corresponding to the number of independent stigma-test performed in blind to determine their SI group (see data in Table S1).

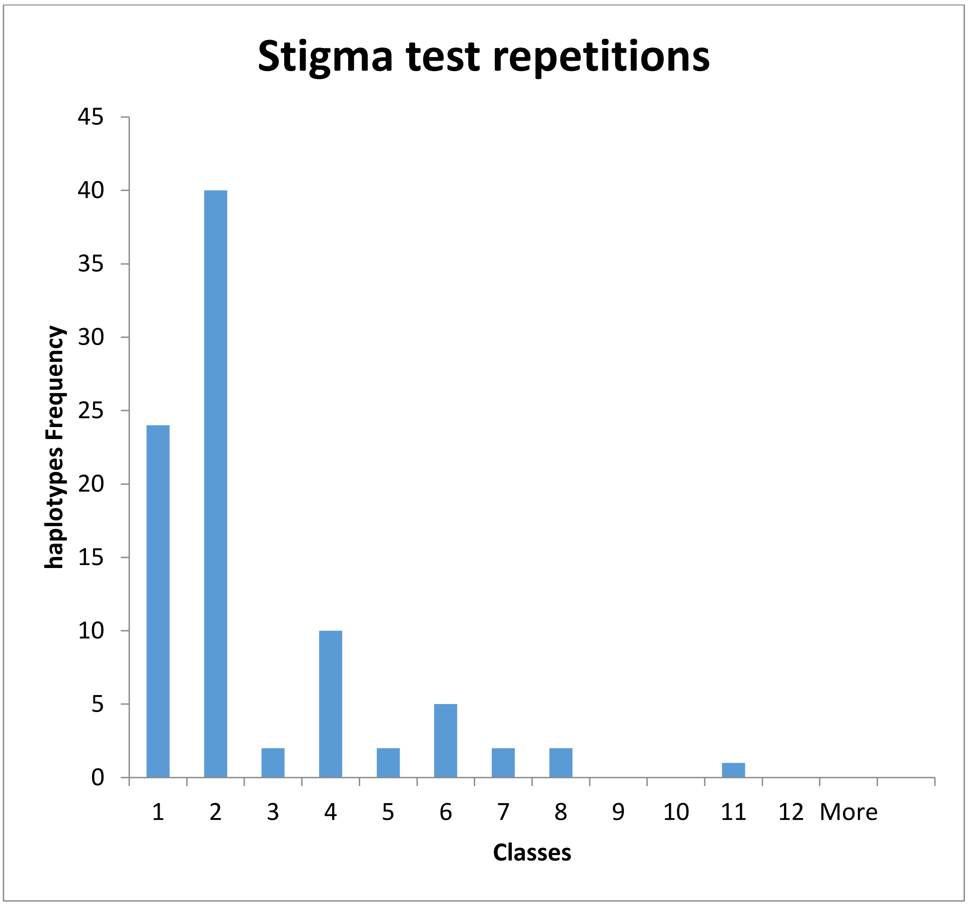

Supplement: Supplementary file 1 [file EVA-10-867-s001.pdf]
