# Supplementary material for: Elucidation of the genetic architecture of self‐incompatibility in olive: Evolutionary consequences and perspectives for orchard management
Source: Evol Appl. 2017 May 20;10(9):867–80. doi: 10.1111/eva.12457 (PMC5680433; doi:10.1111/eva.12457)
Supplement: Supplementary file 2 [file EVA-10-867-s002.pdf]

Fig. S2. Inferred population structure for the domesticated Mediterranean olive tree.

342 genotypes including 309 from the worldwide collection in Marrakech (Morocco) and 33 from Italian and French collections) based on 15 nuclear SSR using model-based Bayesian approach implemented in Structure (Pritchard et al. 2000). According to geographic and genetic criteria, three gene pools were revealed within Mediterranean germplasm (western, central, and eastern groups). Clumpp H' (Jakobsson and Rosenberg 2007) represents the similarity coefficient between 10 runs, and ΔK represents the *ad hoc* measure developed by (Evanno et al. 2005). The membership coefficient of assignment (p) of each individual to different gene pools is shown for K = 3 clusters. We identified 119 admixed genotypes (34.8%; see Table S2-C). The arrows represent the 89 SSR profiles that were phenotyped for self-incompatibility in the present study indicating their distribution into the three Mediterranean gene pools.

References

Evanno, G., S. Regnaut, and J. Goudet. 2005. Detecting the number of clusters of individuals using the software STRUCTURE: a simulation study. *Molecular Ecology* 14 (8):2611-2620.

Jakobsson, M. and N.A. Rosenberg. 2007. CLUMPP: a cluster matching and permutation program for dealing with label switching and multimodality in analysis of population structure. *Bioinformatics* 23 (14):1801-1806.

Pritchard, J.K., M. Stephens, and P. Donnelly. 2000. Inference of Population Structure Using Multilocus Genotype Data. *Genetics* 155 (2):945-959.

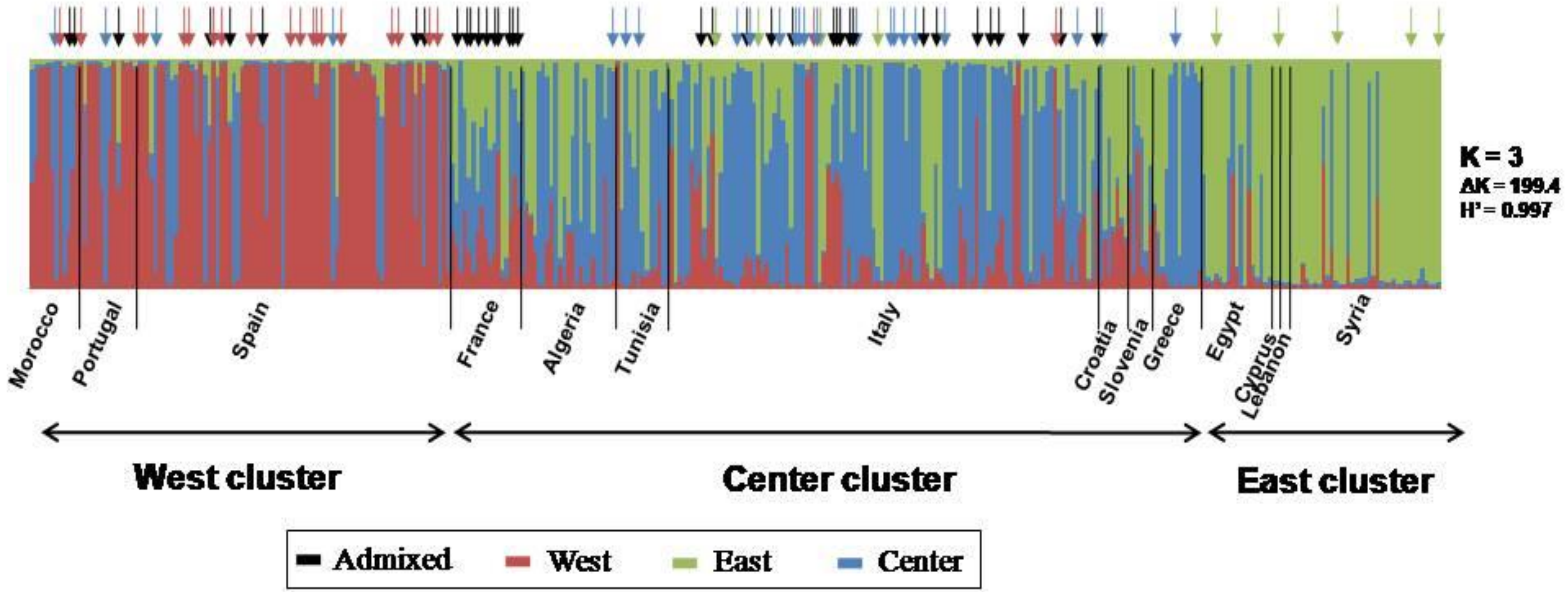
K = 3
ΔK = 199.4
H' = 0.997
Morocco
Portugal
Spain
France
Algeria
Tunisia
Italy
Croatia
Slovenia
Greece
Egypt
Cyprus
Lebanon
Syria
West cluster
Center cluster
East cluster
Admixed
West
East
Center
