# Supplementary material for: Elucidation of the genetic architecture of self‐incompatibility in olive: Evolutionary consequences and perspectives for orchard management
Source: Evol Appl. 2017 May 20;10(9):867–80. doi: 10.1111/eva.12457 (PMC5680433; doi:10.1111/eva.12457)
Supplement: Supplementary file 3 [file EVA-10-867-s003.pdf]

Fig. S3. Two-dimensional distribution of the principal coordinate analysis (PCoA) for the Mediterranean germplasm (filled circles in color) and the 89 genotypes which were phenotyped for self-incompatibly (open squares in color).

The three Mediterranean gene pools represented by red (western gene pool), blue (central) and green (eastern) were defined on the basis of Bayesian model clustering using Structure software (Pritchard et al. 2000; see Fig. S2). The first two principal axes, accounting for 11.42% of the total genetic variation (first axis = 6.56% and the second = 4.86% of the total genetic variation), were sufficient to describe the genetic structure previously defined. Note that the 89 SI genotypes span the range of all genotypes among the three gene pools indicating their representativeness of the domesticated Mediterranean olive.

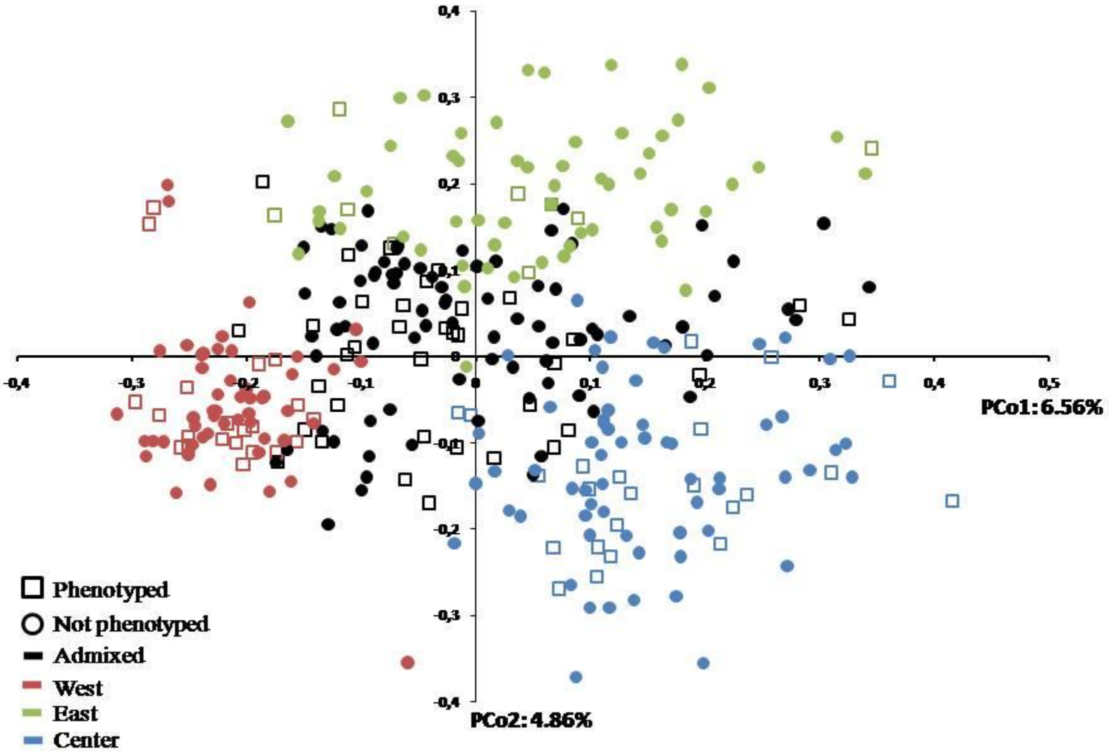

Reference

Pritchard, J.K., M. Stephens, and P. Donnelly. 2000. Inference of Population Structure Using Multilocus Genotype Data. *Genetics* 155 (2):945-959.
