# Supplementary material for: Elucidation of the genetic architecture of self‐incompatibility in olive: Evolutionary consequences and perspectives for orchard management
Source: Evol Appl. 2017 May 20;10(9):867–80. doi: 10.1111/eva.12457 (PMC5680433; doi:10.1111/eva.12457)
Supplement: Supplementary file 4 [file EVA-10-867-s004.pdf]

Table S1. List of the 118 referenced olive trees phenotyped for self-incompatibility (SI) with information on the collection/orchard from which they originate, their position in the orchard, a reference DNA sample code, number of their corresponding genotypes and their SSR profiles. Among these trees, 64 were from the Olive Worldwide Germplasm Bank (OWGB) (Marrakech, Morocco; 45 from the Olive Cultivars of the Perugia collection (PER) (Italy); 3 from the experimental garden in Perugia of CNR – Institute of Biosciences and Bioresources (CNR-IBBR) (Perugia, Italy); and 6 trees from the Conservatoire Botanique National Méditerranéen (CBNMed) (Porquerolles Island, France). Using 15 polymorphic SSR markers, these 118 olive trees were classified into 89 distinct genotypes, some (20 genotypes) being common to 2-5 olive trees per genotype. Each tree was tested for compatibility with G1 and G2 testers: 0 indicates incompatibility was observed, 1 indicates compatibility was observed. Each tree was assigned to G1 or G2 SI group. Assignment was repeated with different pairs of testers: the number indicates the number of conclusive repetitions. Population genetic structure enabled to assign each genotype to a gene pool (see Table S5-C for details).

Collection / Orchard	Position in collection / orchard	Code accession OWGB	Reference DNA sample code	compatibility with G1	compatibility with G2	compatibility with G1 + G2	SI Phenotype	repetitions of stigma test	Self-Incompatibility	Self Compatibility	Gene pool	Genotype	DCA01	DCA01	DCA03	DCA03	DCA04	DCA04	DCA05	DCA05	DCA08	DCA08	DCA09	DCA09	DCA11	DCA11	DCA14	DCA14	DCA15	DCA15	DCA18	DCA18	EMO03	EMO03	EMO90	EMO90	GAPU59	GAPU59	GAPU71B	GAPU71B	UDO36	UDO36
OWGB	I-2-23	Oct-101	Oc0999	1	0	0	G2	2			Admixed	1	203	267	236	240	128	161	194	202	135	139	179	191	129	169	178	186	242	242	172	176	209	212	183	189	210	210	120	139	144	144
PER	B-22		Oit07	1	0	0	G2	1	1	0	Admixed	1	203	267	236	240	128	161	194	202	135	139	179	191	129	169	178	186	242	242	172	176	209	212	183	189	210	210	120	139	144	144
PER	C-27		Oit39	1	0	0	G2	1	1	0	Admixed	1	203	267	236	240	128	161	194	202	135	139	179	191	129	169	178	186	242	242	172	176	209	212	183	189	210	210	120	139	144	144
OWGB	I-2-19	Oct-088	Oc1211	0	1	0	G1	2			Center	2	211	271	240	240	128	128	202	202	135	139	159	179	129	177	176	186	242	262	172	174	207	212	183	189	210	210	120	120	140	144
OWGB	I-7-13	Oct-022	Oc1632	0	1	0	G1	2			Center	2	211	271	240	240	128	128	202	202	135	139	159	179	129	177	176	186	242	262	172	174	207	212	183	189	210	210	120	120	140	144
OWGB	I-6-5	Oct-103	Oc1207	0	1	0	G1	2			Center	3	203	267	234	240	128	130	194	202	133	139	179	203	129	177	178	186	242	262	172	174	212	212	183	189	206	210	120	139	144	164
OWGB	I-6-7	Oct-039	Oc1311	0	1	0	G1	1			Center	3	203	267	234	240	128	130	194	202	133	139	179	203	129	177	178	186	242	262	172	174	212	212	183	189	206	210	120	139	144	164
OWGB	I-7-7	Oct-024	Oc1664	0	1	0	G1	2			Center	3	203	267	234	240	128	130	194	202	133	139	179	203	129	177	178	186	242	262	172	174	212	212	183	189	206	210	120	139	144	164
OWGB	I-14-21	Oct-065	Oc1738	0	1	0	G1	2			Center	3	203	251	234	240	128	130	194	202	133	139	179	203	129	177	178	186	242	262	172	174	212	212	183	189	206	210	120	139	144	164
PER	D-07		Oit25	0	1	0	G1	4	1	0	Center	3	203	267	234	240	128	130	194	202	133	139	179	203	129	129	178	186	242	262	172	174	212	212	183	189	206	210	120	139	140	144
OWGB	I-7-46	Oct-348	Oc1519	1	0	0	G2	2			Western	4	203	213	236	250	130	161	202	202	135	137	159	203	139	173	186	186	242	253	168	172	207	211	183	185	210	210	117	126	142	144
OWGB	I-7-44	Oct-347	Oc1630	1	0	0	G2	2			Western	4	203	213	236	250	130	161	202	202	135	137	159	203	139	173	186	186	242	253	168	172	207	211	183	185	210	210	117	126	142	144
OWGB	I-15-1	Oct-147	Oc1656	0	1	0	G1	2			Western	5	203	203	246	250	161	161	202	202	135	135	159	191	159	177	176	186	242	262	172	186	207	209	183	183	210	210	117	139	140	144
PER	R-12		Oit04	0	1	0	G1	1	1	0	Western	5	203	203	246	250	161	161	202	202	135	135	159	191	159	177	176	186	242	262	172	180	207	209	183	183	210	210	117	139	140	144
PER	O-37		Oit12	0	1	0	G1	3			Western	5	203	203	246	250	161	161	202	202	135	135	159	191	159	177	176	186	242	262	172	180	207	209	183	183	210	210	117	139	140	144
PER	I-17		Oit08	1	0	0	G2	1			Center	6	211	267	234	250	128	130	202	202	139	145	203	203	129	139	169	178	253	262	172	172	205	212	183	183	206	210	139	139	144	144
PER	A-07		Oit17	1	0	0	G2	4	1	0	Center	6	211	267	234	250	128	130	202	202	139	145	203	203	129	139	169	178	253	262	172	172	205	212	183	183	206	210	139	139	144	144
PER	H-37		Oit42	0	1	0	G1	1			Admixed	7	203	213	240	240	130	130	196	202	131	135	173	191	145	145	174	186	242	259	170	178	210	212	183	183	206	210	120	122	140	142
PER	D-28		Oit51	0	1	0	G1	1	1	0	Admixed	7	203	213	240	240	130	130	196	202	131	135	173	191	145	145	174	186	242	259	170	178	210	212	183	183	206	210	120	122	140	142
OWGB	I-3-32	Oct-226	Oc1560	0	1	0	G1	2			Western	8	203	211	242	246	130	165	202	202	135	137	181	191	139	177	186	186	242	253	168	176	207	215	183	185	210	210	120	139	140	142
OWGB	I-3-21	Oct-002	Oc1610	1	0	0	G2	2			Center	9	203	231	229	229	128	132	196	202	127	133	199	199	133	145	174	174	242	265	168	170	211	211	183	189	221	221	120	120	142	162
PER	A-32		Oit34	0	1	0	G1	1	1	0	Center	10	211	211	236	236	148	186	190	204	129	133	169	191	139	177	174	182	253	262	168	172	209	212	181	185	206	210	120	126	142	144
OWGB	I-3-19	Oct-005	Oc1594	1	0	0	G2	2			Eastern	11	203	203	229	250	128	161	190	202	133	135	159	195	145	177	174	186	242	242	174	176	209	211	183	193	210	216	117	126	140	146
PER	F-18		Oit30	1	0	0	G2	4	1	0	Eastern	11	203	211	229	250	128	161	190	202	133	135	159	195	145	177	174	186	242	242	174	176	209	211	183	193	210	216	117	126	144	144
OWGB	I-11-15	Oct-008	Oc1566	0	1	0	G1	1			Center	12	203	213	236	250	128	148	194	204	133	133	163	201	139	159	176	178	262	265	164	168	205	207	183	189	210	221	120	122	144	146
OWGB	I-11-5	Oct-013	Oc1618	1	0	0	G2	2			Admixed	13	203	211	240	246	182	182	202	202	135	137	159	169	145	177	174	186	242	242	168	172	207	212	183	189	210	210	139	139	144	144
PER	D-25		Oit03	1	0	0	G2	2	1	0	Center	14	211	264	229	240	128	186	190	210	125	133	169	169	129	181	178	186	262	262	172	174	210	212	181	185	206	206	120	126	144	144
CNR-IBBR	L14-P2		Oit27	0	1	0	G1	4	1	0	Admixed	15	203	257	240	250	128	130	194	202	133	135	159	203	129	177	176	178	253	262	172	172	207	212	183	189	206	210	120	139	144	144
PER	C-4		Oit65	0	1	0	G1	4	1	0	Admixed	15	203	257	240	250	128	130	194	202	133	135	159	203	129	177	176	178	253	262	172	172	207	212	183	189	206	210	120	139	144	144
PER	O-35		Oit19	1	0	0	G2	1			Admixed	16	203	213	236	246	128	161	194	202	135	139	179	191	145	177	174	186	242	242	168	176	209	211	183	183	210	221	122	139	142	144
OWGB	I-7-9	Oct-023	Oc1659	0	1	0	G1	2			Admixed	17	203	211	242	246	128	186	202	206	135	139	191	203	139	177	169	186	242	253	168	170	212	215	183	183	212	221	117	120	140	140
OWGB	I-7-17	Oct-025	Oc1607	1	0	0	G2	1			Admixed	18	203	213	234	246	128	128	202	202	135	141	181	191	139	159	169	176	242	253	170	172	207	215	183	183	210	212	117	120	140	148
OWGB	I-7-15	Oct-027	Oc1642	0	1	0	G1	2			Center	19	211	267	234	250	128	128	202	202	133	135	179	203	129	139	169	178	242	253	172	174	207	212	183	189	206	210	120	139	140	164
OWGB	I-6-19	Oct-043	Oc1308	0	1	0	G1	2			Eastern	20	203	211	229	242	128	159	202	204	129	129	183	201	139	145	174	186	242	242	166	172	209	211	181	193	206	216	120	122	142	144
OWGB	I-10-9	Oct-045	Oc1872	1	0	0	G2	2			Center	21	213	217	236	240	159	186	202	202	139	143	179	181	129	177	186	186	242	262	170	174	212	212	183	189	221	223	120	122	140	162
PER	C-33		Oit36	1	0	0	G2	4	1	0	Center	22	211	213	240	252	130	132	194	202	129	145	169	181	139	139	178	186	242	262	158	174	211	211	183	193	216	221	120	126	144	144
OWGB	I-10-15	Oct-052	Oc1856	0	1	0	G1	2			Center	23	211	213	229	240	128	186	202	202	137	139	181	181	139	177	169	186	242	253	174	180	212	215	183	189	210	221	120	126	140	144
PER	E-51		Oit24	0	1	0	G1	2	1	0	Center	23	211	213	229	240	128	186	202	202	137	139	181	203	139	177	169	186	242	253	174	180	212	215	183	189	210	221	120	126	140	144
OWGB	I-14-3	Oct-063	Oc1795	1	0	0	G2	2			Admixed	24	203	203	236	236	128	130	202	204	123	133	201	207	139	139	186	186	242	262	168	172	202	211	181	185	206	206	120	126	144	144
OWGB	I-24-5	Oct-084	Oc1408	1	0	0	G2	2			Admixed	25	203	203	236	250	138	138	190	208	135	137	163	191	139	159	169	186	262	262	172	174	211	214	183	183	210	221	117	120	140	164
OWGB	I-3-15	Oct-091	Oc1586	1	0	0	G2	1			Admixed	26	203	203	242	250	138	138	198	202	129	135	159	169	139	139	186	186	253	262	172	174	207	211	183	183	210	210	126	139	140	142
PER	O-30		Oit11	0	1	0	G1	1			Western	27	203	203	246	250	161	161	202	202	135	135	159	191	159	181	176	186	242	262	172	176	207	209	183	183	210	210	117	139	140	144
OWGB	I-10-19	Oct-120	Oc1845	0	1	0	G1	2			Eastern	28	203	203	240	250	128	161	202	204	133	135	159	169	175	175	176	186	242	262	174	178	207	212	183	189	210	210	122	139	144	144
OWGB	I-11-13	Oct-124	Oc1561	0	1	0	G1	2			Eastern	28	203	203	240	250	128	165	202	204	133	135	159	169	173	177	176	186	242	262	174	178	207	212	183	189	210	210	122	139	144	144
OWGB	I-22-13	Oct-149	Oc1572	0	1	0	G1	2			Eastern	28	203	203	240	250	128	157	202	204	133	135	159	169	175	177	176	186	242	262	174	178	207	212	183	189	210	210	122	139	144	144
PER	C-24		Oit20	0	1	0	G1	2			Admixed	29	203	211	242	246	186	188	202	202	129	135	183	191	175	177	176	186	242	262	168	176	209	211	181	183	206	210	117	139	144	144
OWGB	I-15-5	Oct-145	Oc1451	1	0	0	G2	2			Admixed	30	203	213	238	246	142	161	198	202	135	135	159	183	125	159	169	176	262	262	166	172	205	207	183	193	206	210	120	142	140	140
OWGB	I-15-7	Oct-150	Oc1628	0	1	0	G1	2			Center	31	203	213	229	250	128	132	194	204	123	135	159	177	129	177	178	186	253	262	172	178	205	207	183	183	210	221	122	122	144	162
PER	C-27		Oit37	1	0	0	G2	1	1	0	Admixed	32	203	211	240	250	161	192	202	202	129	135	183	191	159	177	176	178	262	262	166	172	209	212	181	183	206	210	117	122	142	144
PER	J-40		Oit38	1	0	0	G2	2	1	0	Admixed	32	203	211	240	250	161	192	202	202	129	135	183	191	159	177	176	178	262	262	166	172	209	212	181	183	206	210	117	122	142	144
OWGB	I-22-30	Oct-189	Oc1531	1	0	0	G2	2			Admixed	33	203	211	229	246	130	161	190	202	133	135	159	169	147	159	174	176	242	262	166	176	207	212	183	189	210	216	139	139	140	144
PER	R-17		Oit43	0	1	0	G1	1			Admixed	34	203	211	240	246	184	186	202	202	133	135	159	169	145	159	174	176	242	262	168	172	209	211	183	185	210	210	117	139	142	144
PER	C-12		Oit18	0	1	0	G1	1			Admixed	35	203	264	229	250	159	159	198	202	135	137	191	191	129	177	186	186	242	262	166	176	209	211	183	189	210	210	120	139	144	150
PER	M-40		Oit46	1	0	0	G2	4	1	0	Admixed	36	203	211	236	250	130	161	190	202	135	137	159	169	145	177	176	186	242	242	174	176	209	212	183	189	210	210	126	139	140	142
OWGB	I-19-26	Oct-207	Oc1001	1	0	0	G2	2			Admixed	37	203	211	236	250	174	174	202	202	135	137	159	203	145	159	174	186	242	262	168	172	209	211	183	189	210	210	126	139	144	144
OWGB	I-2-26	Oct-215	Oc1220	1	0	0	G2	2			Western	38	203	213	236	250	138	138	202	202	137	139	159	203	159	177	176	186	242	262	166	176	207	211	183	185	210	210	126	139	140	142
OWGB	I-2-28	Oct-216	Oc1205	1	0	0	G2	2			Western	39	203	213	236	246	130	161	202	202	135	139	191	203	177	177	186	186	242	262	166	176	207	211	183	183	210	221	117	120	140	140
OWGB	I-2-36	Oct-220	Oc1873	0	1	0	G1	2			Center	40	203	213	229	240	130	159	198	202	137	137	181	203	139	177	186	186	242	262	164	174	211	211	183	189	221	221	120	139	142	150
CNR-IBBR	L9-P49		Oit26	0	1	0	G1	4	1	0	Center	40	203	213	229	240	130	159	198	202	137	137	181	203	139	177	186	186	242	262	164	174	211	211	183	189	221	221	120	139	140	142
OWGB	I-3-30	Oct-225	Oc1587	0	1	0	G1	2			Western	41	203	213	236	246	130	161	202	202	135	137	181	191	139	159	176	186	253	262	166	176	209	215	183	183	210	221	117	120	140	144
OWGB	I-6-26	Oct-231	Oc1227	0	1	0	G1	2			Admixed	42	203	211	236	250	130	130	202	206	135	137	191	201	165	177	176	186	262	262	172	176	207	211	183	185	206	210	122	139	140	142
PER	L-25		Oit41	0	1	0	G1	1			Western	43	203	213	236	246	130	161	202	202	135	139	181	191	159	177	186	188	242	262	168	176	207	211	183	183	210	210	120	139	142	148
OWGB	I-6-30	Oct-233	Oc1303	1	0	0	G2	2			Western	44	203	211	242	250	130	130	202	202	135	137	159	199	139	159	176	186	253	262	168	176	207	211	183	185	210	210	120	139	140	144
OWGB	I-10-32	Oct-251	Oc1847	1	0	0	G2	2			Western	44	203	211	242	250	130	130	202	202	135	137	159	203	139	159	176	186	253	262	168	176	207	211	183	185	210	210	120	139	140	144
PER	M-19		Oit01	1	0	0	G2	1	1	0	Western	44	203	211	242	250	130	130	202	202	135	137	159	203	139	159	176	186	253	262	168	176	207	211	183	185	210	210	120	139	140	144
PER	D-13		Oit57	1	0	0	G2	2	1	0	Western	44	203	211	242	250	130	130	202	202	135	137	159	203	139	159	176	186	253	262	168	176	207	211	183	185	210	210	120	139	140	144
OWGB	I-6-36	Oct-236	Oc1258	0	1	0	G1	2			Admixed	45	203	203	242	246	130	130	202	202	135	139	181	191	145	159	176	180	242	242	168	172	209	211	183	185	210	210	117	122	140	144
OWGB	I-7-28	Oct-241	Oc1562	0	1	0	G1	2			Admixed	45	203	203	242	246	130	130	202	202	135	139	181	191	145	163	176	180	242	242	168	172	209	211	183	185	210	210	117	122	140	144
OWGB	I-7-26	Oct-240	Oc1624	0	1	0	G1	2			Western	46	203	213	236	246	138	161	202	202	135	137	191	203	139	139	186	186	242	253	168	176	207	211	183	185	210	221	120	139	140	140
PER	L-22		Oit05	0	1	0	G1	1	1	0	Western	46	203	213	236	246	138	161	202	202	135	137	191	203	139	139	186	186	242	242	168	176	207	211	183	185	210	221	120	139	140	140
OWGB	I-7-32	Oct-243	Oc1507	0	1	0	G1	2			Admixed	47	203	213	242	246	138	138	198	202	135	139	159	203	139	177	169	186	242	253	164	172	207	211	183	183	210	210	120	139	140	144
PER	K-22		Oit14	0	1	0	G1	2			Admixed	47	203	213	242	246	138	138	198	202	135	139	159	203	139	177	169	186	242	253	164	172	207	211	183	183	210	210	120	139	140	144
OWGB	I-7-40	Oct-247	Oc1621	1	0	0	G2	2			Western	48	203	211	242	246	138	161	202	202	135	137	191	205	139	159	186	186	242	253	166	176	207	211	183	183	210	221	117	126	142	144
OWGB	I-11-26	Oct-256	Oc1666	1	0	0	G2	2			Western	49	203	213	236	250	138	161	202	202	135	137	181	191	159	177	186	186	242	262	166	172	207	215	183	185	210	210	117	126	142	144
PER	L-19		Oit10	1	0	0	G2	4	1	0	Western	50	203	211	242	250	130	130	202	202	135	139	159	181	159	177	176	186	262	262	168	176	209	211	183	183	210	221	117	120	140	144
OWGB	I-14-32	Oct-267	Oc1275	1	0	0	G2	2			Western	51	203	213	236	246	130	130	202	202	135	139	181	189	139	175	186	186	242	253	166	172	207	215	183	183	210	221	117	126	140	140
PER	K-26		Oit02	1	0	0	G2	4	1	0	Western	51	203	213	236	246	130	130	202	202	135	139	181	189	139	175	186	186	242	253	166	172	207	215	183	183	210	221	117	126	140	140
PER	K-25		Oit60	1	0	0	G2	2			Western	51	203	213	236	246	130	130	202	202	135	139	181	189	139	175	186	186	242	253	166	172	207	215	183	183	210	221	117	126	140	140
PER	M-19		Oit56	1	0	0	G2	1	1	0	Western	52	203	213	236	246	130	130	202	202	135	139	181	189	139	175	186	186	242	253	166	172	207	215	181	183	210	221	117	126	142	148
OWGB	I-14-34	Oct-268	Oc1792	1	0	0	G2	2			Western	53	203	213	242	250	130	130	202	202	135	137	159	181	159	177	176	186	253	262	166	172	207	211	183	185	210	210	117	120	140	142
OWGB	I-14-40	Oct-271	Oc1870	1	0	0	G2	2			Admixed	54	203	213	229	246	128	186	190	202	129	135	169	191	159	179	182	186	242	262	168	176	207	211	183	189	210	226	120	120	140	164
OWGB	I-15-28	Oct-273	Oc1605	0	1	0	G1	2			Western	55	203	211	242	246	130	165	202	202	135	139	191	203	139	159	176	186	253	262	168	172	209	215	183	183	210	221	120	139	140	144
OWGB	I-15-32	Oct-275	Oc1601	1	0	0	G2	2			Western	56	203	211	236	246	130	130	202	202	135	139	159	181	159	177	176	186	242	262	168	176	207	211	183	183	210	221	117	120	142	144
OWGB	I-6-48	Oct-345	Oc1318	1	0	0	G2	2			Western	57	203	211	236	246	130	161	202	202	135	139	191	203	159	177	176	186	262	262	168	176	209	211	183	183	210	210	117	126	140	140
OWGB	I-10-42	Oct-350	Oc1848	0	1	0	G1	2			Center	58	211	211	240	242	184	192	194	202	137	150	183	205	145	145	169	186	242	242	168	180	209	211	185	185	210	221	122	126	144	144
OWGB	I-11-48	Oct-358	Oc1611	0	1	0	G1	2			Admixed	59	203	215	236	250	142	142	202	204	135	150	159	179	129	159	169	186	242	262	170	172	207	211	183	183	210	226	122	139	144	144
PER	G-41		Oit47	1	0	0	G2	1	1	0	Center	60	211	213	236	236	130	148	198	202	133	152	169	181	139	145	174	178	242	253	162	174	211	211	185	185	210	221	122	139	142	150
OWGB	II-14-17	Oct-413	Oc1676	0	1	0	G1	2			Center	61	203	221	234	236	139	186	202	212	133	145	169	192	145	149	169	174	242	253	168	176	206	212	183	185	206	221	120	126	144	162
OWGB	II-8-16	Oct-416	Oc0960	0	1	0	G1	2			Center	62	211	225	229	240	130	186	190	202	129	137	169	192	145	151	169	174	242	265	172	174	211	214	185	193	216	216	120	126	142	144
OWGB	II-10-16	Oct-421	Oc0891	0	1	0	G1	2			Center	63	203	213	229	231	130	159	194	202	133	137	197	203	139	177	169	176	253	262	166	168	209	214	183	189	210	226	120	122	144	144
OWGB	II-18-22	Oct-480	Oc1719	0	1	0	G1	2			Eastern	64	203	213	229	250	159	159	202	202	135	152	159	179	145	151	176	186	242	259	166	180	209	211	183	183	210	210	117	120	142	144
OWGB	II-6-19	Oct-508	Oc1842	0	1	0	G1	2			Center	65	203	213	236	240	130	186	202	202	133	139	181	201	139	139	169	186	253	262	172	180	212	212	181	183	206	210	120	120	144	144
OWGB	II-8-19	Oct-538	Oc1802	1	0	0	G2	2			Center	66	203	213	227	229	128	130	202	202	123	129	181	203	129	177	178	178	262	262	172	176	210	212	183	183	221	221	120	126	150	155
OWGB	II-13-23	Oct-541	Oc1013	1	0	0	G2	2			Western	67	203	211	236	250	130	130	202	202	135	137	191	203	139	159	176	186	242	253	168	176	209	211	183	185	210	221	117	126	140	144
PER	E-16		Oit22	1	0	0	G2	5	1	0	Western	67	203	211	236	250	130	130	202	202	135	137	191	203	139	159	176	186	242	253	168	176	209	211	183	185	210	221	117	126	140	144
OWGB	II-16-13	Oct-546	Oc1734	0	1	0	G1	2			Admixed	68	203	215	229	236	130	144	198	202	125	135	187	203	139	159	169	186	253	253	168	176	211	211	183	183	221	221	122	126	144	162
OWGB	II-10-13	Oct-547	Oc1852	0	1	0	G1	2			Admixed	69	211	227	236	250	130	138	198	202	123	135	183	203	125	159	176	176	242	253	162	176	202	209	183	183	210	221	122	126	140	140
PER	H-20		Oit29	1	0	0	G2	2	1	0	Eastern	70	203	211	242	246	159	159	202	202	129	133	169	191	145	159	182	186	242	262	172	172	211	211	181	183	206	210	120	122	144	144
OWGB	II-16-13	Oct-603	Oc1731	1	0	0	G2	2			Eastern	71	203	203	246	250	159	159	202	202	125	137	185	191	145	159	174	176	242	261	170	178	210	210	181	197	210	210	117	139	140	140
OWGB	II-6-11	Oct-630	Oc0989	0	1	0	G1	2			Eastern	72	203	203	229	246	159	161	200	202	133	133	195	201	149	155	171	182	242	262	166	172	209	211	183	183	216	239	120	144	142	142
OWGB	II-17-11	Oct-641	Oc1697	0	1	0	G1	2			Eastern	73	203	203	234	246	159	164	196	202	135	137	173	185	159	177	176	186	242	261	168	170	211	211	183	197	210	210	117	144	138	140
PER	A-27		Oit35	1	0	0	G2	1	1	0	Admixed	74	203	267	240	250	128	128	202	202	133	135	159	203	139	173	169	186	253	262	172	174	207	212	183	189	210	210	120	139	140	142
PER	N-21		Oit28	1	0	0	G2	2	1	0	Western	75	203	213	236	250	130	161	202	202	135	137	159	181	139	159	176	186	253	262	168	172	207	211	183	185	210	210	117	126	140	142
PER	N-32		Oit09	0	1	0	G1	1			Eastern	76	203	203	246	250	128	159	202	204	135	135	159	179	171	181	176	176	242	262	166	172	207	209	183	183	210	210	116	120	140	144
PER	L-42		Oit45	0	1	0	G1	1			Admixed	77	203	203	229	240	128	130	190	202	129	133	183	203	139	145	174	186	242	242	168	180	211	211	181	189	206	210	120	126	140	140
PER	L-33		Oit55	1	0	0	G2	1	1	0	Center	78	203	203	229	240	186	190	190	202	137	145	179	203	139	145	174	178	259	262	158	166	212	212	183	185	206	206	126	139	144	164
PER	OF-1*		Oit15	1	0	0	G2	4	1	0	Admixed	79	203	211	229	242	159	188	190	204	129	133	169	183	139	145	174	178	242	262	166	168	209	211	181	181	206	221	120	126	142	148
CNR-IBBR	L14-P1		Oit49	1	0	0	G2	1			Admixed	79	203	211	229	242	159	186	190	204	129	133	169	183	129	145	174	178	242	262	166	168	209	211	181	181	206	221	120	120	142	148
PER	J-27		Oit52	0	1	0	G1	1			Center	80	203	239	236	236	130	152	190	190	137	145	179	203	139	145	174	174	259	262	168	170	209	212	183	189	206	210	120	126	140	140
PER	H-2		Oit58	1	0	0	G2	5	1	0	Admixed	81	203	211	240	250	130	130	190	202	133	135	159	169	175	177	186	186	262	262	168	180	209	212	183	189	210	210	117	126	140	144
PER	F-28		Oit59	0	1	0	G1	1			Admixed	82	203	213	240	250	167	167	190	202	135	139	159	181	139	159	176	186	242	253	168	180	209	211	183	183	210	221	120	139	140	144
PER	E-10		Oit62	0	1	0	G1	1	1	0	Center	83	211	271	240	240	128	128	202	202	133	145	179	203	129	177	176	186	242	253	174	174	207	212	183	189	210	210	120	139	144	144
CBNMed	33-25		33-25	1	0	0	G2	1			Admixed	84	203	203	240	246	161	186	194	202	133	135	159	169	139	139	186	186	242	262	168	172	207	209	183	185	210	210	117	122	142	148
CBNMed	35-07		35-07	1	0	0	G2	1			Admixed	85	203	211	240	246	161	186	202	206	137	139	183	203	139	177	169	186	242	242	166	174	209	215	183	189	210	221	120	139	140	144
CBNMed	33-10		Oc1911	1	0	0	G2	1			Admixed	86	211	211	229	246	159	161	202	206	133	137	159	203	139	139	169	186	242	262	166	176	207	215	185	189	206	210	120	139	140	144
CBNMed	33-13		Oc1912	1	0	0	G2	1			Admixed	87	203	211	236	250	161	161	194	202	127	135	179	191	129	159	176	186	242	262	168	172	207	211	183	183	206	210	126	139	144	144
CBNMed	33-22		Oc1919	1	0	0	G2	1			Admixed	88	203	213	236	246	159	159	194	202	131	135	159	191	129	177	178	186	242	262	166	174	207	209	183	185	210	210	117	122	140	142
CBNMed	34-25		Oc1924	1	0	0	G2	1			Admixed	89	203	203	234	242	130	159	202	202	139	139	169	179	155	177	176	186	242	262	172	174	212	215	183	185	206	206	117	120	142	164

* Out of field (big tree close to the A line)
