# Supplementary material for: Elucidation of the genetic architecture of self‐incompatibility in olive: Evolutionary consequences and perspectives for orchard management
Source: Evol Appl. 2017 May 20;10(9):867–80. doi: 10.1111/eva.12457 (PMC5680433; doi:10.1111/eva.12457)
Supplement: Supplementary file 5 [file EVA-10-867-s005.pdf]

Table S2. List of the 91 LEDA trees from the (Oit64 x Oit27) controlled cross phenotyped for self-incompatibility (SI) with information on their position in the experimental garden at the CNR in Perugia, (CNR-IBBR) Italy. Each tree was tested for compatibility with G1 and G2 testers: 0 means incompatibility observed, 1 means compatibility observed. Each tree was assigned to G1 or G2 SI group.

Collection / Orchard	Position in collection / orchard Lane 1-12/1-20/row	Sample name	compatibility with G1	compatibility with G2	compatibility with G1 + G2	Phenotype	Collection / Orchard	Position in collection / orchard Lane 1-12/1-20/row	Sample name	compatibility with G1	compatibility with G2	compatibility with G1 + G2	Phenotype
CNR-IBBR		LEDA/FR56	1	0	0	G2	CNR-IBBR	04/02	LEDA_242	1	0	0	G2
CNR-IBBR		LEDA/FR89	0	1	0	G1	CNR-IBBR	04/04	LEDA_244	1	0	0	G2
CNR-IBBR	07/18	LEDA_008	1	0	0	G2	CNR-IBBR	04/05	LEDA_245	0	1	0	G1
CNR-IBBR	07/19	LEDA_009	1	0	0	G2	CNR-IBBR	04/06	LEDA_246	0	1	0	G1
CNR-IBBR	08/04	LEDA_016	0	1	0	G1	CNR-IBBR	04/07	LEDA_247	0	1	0	G1
CNR-IBBR	08/05	LEDA_017	0	1	0	G1	CNR-IBBR	04/09	LEDA_249	0	1	0	G1
CNR-IBBR	8/11	LEDA_026	1	0	0	G2	CNR-IBBR	04/11	LEDA_251	1	0	0	G2
CNR-IBBR	8/12	LEDA_028	1	0	0	G2	CNR-IBBR	04/12	LEDA_252	0	1	0	G1
CNR-IBBR	8/13	LEDA_029	1	0	0	G2	CNR-IBBR	04/15	LEDA_255	1	0	0	G2
CNR-IBBR	8/14	LEDA_030	1	0	0	G2	CNR-IBBR	04/17	LEDA_257	0	1	0	G1
CNR-IBBR	8/20	LEDA_039	1	0	0	G2	CNR-IBBR	04/19	LEDA_259	1	0	0	G2
CNR-IBBR	9/02	LEDA_041	1	0	0	G2	CNR-IBBR	04/20	LEDA_260	1	0	0	G2
CNR-IBBR	9/03	LEDA_045	1	0	0	G2	CNR-IBBR	05/01	LEDA_261	1	0	0	G2
CNR-IBBR	9/07	LEDA_051	1	0	0	G2	CNR-IBBR	05/02	LEDA_262	1	0	0	G2
CNR-IBBR	9/08	LEDA_052	1	0	0	G2	CNR-IBBR	05/03	LEDA_263	1	0	0	G2
CNR-IBBR	9/09	LEDA_053	1	0	0	G2	CNR-IBBR	05/04	LEDA_264	1	0	0	G2
CNR-IBBR	9/10	LEDA_054	0	1	0	G1	CNR-IBBR	05/06	LEDA_266	1	0	0	G2
CNR-IBBR	9/11	LEDA_055	1	0	0	G2	CNR-IBBR	05/07	LEDA_267	0	1	0	G1
CNR-IBBR	9/13	LEDA_057	1	0	0	G2	CNR-IBBR	05/08	LEDA_268	0	1	0	G1
CNR-IBBR	9/17	LEDA_062	0	1	0	G1	CNR-IBBR	05/10	LEDA_270	0	1	0	G1
CNR-IBBR	10/01	LEDA_070	0	1	0	G1	CNR-IBBR	05/12	LEDA_272	0	1	0	G1
CNR-IBBR	10/05	LEDA_075	0	1	0	G1	CNR-IBBR	05/13	LEDA_273	0	1	0	G1
CNR-IBBR	10/06	LEDA_077	0	1	0	G1	CNR-IBBR	05/15	LEDA_275	0	1	0	G1
CNR-IBBR	10/07	LEDA_078	1	0	0	G2	CNR-IBBR	05/19	LEDA_279	0	1	0	G1
CNR-IBBR	19/09	LEDA_081	0	1	0	G1	CNR-IBBR	06/02	LEDA_282	1	0	0	G2
CNR-IBBR	10/10	LEDA_083	0	1	0	G1	CNR-IBBR	06/05	LEDA_285	0	1	0	G1
CNR-IBBR	10/16	LEDA_092	1	0	0	G2	CNR-IBBR	06/06	LEDA_286	0	1	0	G1
CNR-IBBR	10/19	LEDA_095	1	0	0	G2	CNR-IBBR	06/07	LEDA_287	1	0	0	G2
CNR-IBBR	02/01	LEDA_201	0	1	0	G1	CNR-IBBR	06/09	LEDA_289	0	1	0	G1
CNR-IBBR	02/04	LEDA_204	1	0	0	G2	CNR-IBBR	06/10	LEDA_290	1	0	0	G2
CNR-IBBR	02/06	LEDA_206	0	1	0	G1	CNR-IBBR	06/11	LEDA_291	0	1	0	G1
CNR-IBBR	02/09	LEDA_209	1	0	0	G2	CNR-IBBR	06/13	LEDA_293	1	0	0	G2
CNR-IBBR	02/10	LEDA_210	0	1	0	G1	CNR-IBBR	06/14	LEDA_294	0	1	0	G1
CNR-IBBR	02/11	LEDA_211	0	1	0	G1	CNR-IBBR	06/16	LEDA_296	1	0	0	G2
CNR-IBBR	02/12	LEDA_212	1	0	0	G2	CNR-IBBR	06/17	LEDA_297	0	1	0	G1
CNR-IBBR	02/16	LEDA_216	0	1	0	G1	CNR-IBBR	06/19	LEDA_299	1	0	0	G2
CNR-IBBR	02/17	LEDA_217	1	0	0	G2	CNR-IBBR	07/01	LEDA_301	1	0	0	G2
CNR-IBBR	02/18	LEDA_218	1	0	0	G2	CNR-IBBR	07/02	LEDA_302	1	0	0	G2
CNR-IBBR	03/02	LEDA_222	1	0	0	G2	CNR-IBBR	07/03	LEDA_303	0	1	0	G1
CNR-IBBR	03/08	LEDA_228	1	0	0	G2	CNR-IBBR	07/06	LEDA_306	0	1	0	G1
CNR-IBBR	03/09	LEDA_229	1	0	0	G2	CNR-IBBR	07/07	LEDA_307	0	1	0	G1
CNR-IBBR	03/10	LEDA_230	0	1	0	G1	CNR-IBBR	07/08	LEDA_308	0	1	0	G1
CNR-IBBR	03/12	LEDA_232	1	0	0	G2	CNR-IBBR	07/11	LEDA_311	1	0	0	G2
CNR-IBBR	03/16	LEDA_236	1	0	0	G2	CNR-IBBR	07/12	LEDA_312	0	1	0	G1
CNR-IBBR	03/18	LEDA_238	0	1	0	G1	CNR-IBBR	07/15	LEDA_315	1	0	0	G2
CNR-IBBR	03/19	LEDA_239	1	0	0	G2							
