# Supplementary material for: Elucidation of the genetic architecture of self‐incompatibility in olive: Evolutionary consequences and perspectives for orchard management
Source: Evol Appl. 2017 May 20;10(9):867–80. doi: 10.1111/eva.12457 (PMC5680433; doi:10.1111/eva.12457)
Supplement: Supplementary file 6 [file EVA-10-867-s006.pdf]

Table S3. Paternity analysis of seeds harvested in controlled crosses (listed in Table 4) using polymorphic microsatellites. Parents and their progeny were genotyped with 3 to 10 markers. We tested a sample of the seeds collected in each of the seven crosses performed among parents predicted compatible by stigma test (G1xG2) and all of the seeds collected in three of the crosses performed among parents predicted incompatible by the stigma test (G2xG2).

		Microsatellite markers																				
**ID parents and progenies**	**SI GROUP**	**D3**	**D3**	**D9**	**D9**	**D16**	**D16**	**D18**	**D18**	**G101**	**G101**	**G71B**	**G71B**	**U43**	**U43**	**E90**	**E90**	**G103**	**G103**	**D5**	**D5**	**paternity conclusion**
**Oit55 x**	**G2**	231	243	182	206	124	146	163	171	193	207	130	144	176	186	188	190	150	160	194	206	
**Oit15**	**G2**	231	245	172	186	124	146	171	173	191	199	124	124	172	176	186	186	174	174	194	208	
(Oit55 X Oit15)1		243	243	182	206	124	146	171	171	193	207	130	144	186	186	188	190	150	160	194	206	self
(Oit55 X Oit15)2		243	243	182	182	124	124	163	171	193	207	130	130	176	186	188	190	150	160	194	206	self
(Oit55 X Oit15)3		243	243	182	206	124	124	163	171	193	207	130	144	176	176	188	190	150	160	194	206	self
(Oit55 X Oit15)4		231	231	182	182	124	146	163	163	193	207	130	144	176	186	188	190	150	160	194	194	self
(Oit55 X Oit15)5		243	243	182	206	124	124	163	171	193	207	130	144	176	176	190	190	150	160	206	206	self
(Oit55 X Oit15)6		231	231	182	206	146	146	171	171	193	193	130	144	176	186	188	188	150	160	194	206	self
(Oit55 X Oit15)7		243	243	182	182	124	146	163	171	193	193	130	144	176	186	188	188	160	160	194	206	self
(Oit55 X Oit15)8		243	243	206	206	124	146	163	171	207	207	130	144	186	186	190	190	150	150	206	206	self
(Oit55 X Oit15)9		231	231	182	182	124	124	171	171	193	193	130	144	176	176	188	188	150	160	206	206	self
(Oit55 X Oit15)10		231	231	182	206	124	124	163	171	193	207	130	130	176	186	188	190	150	160	194	206	self
**Oit55 x**	**G2**	231	243	182	206	124	146	nd	nd	nd	nd	nd	nd	176	186	188	190	150	160	194	206	
**Oit27**	**G1**	243	253	162	206	150	174	177	177	199	201	124	144	210	214	188	194	174	186	198	206	
(Oit55 X Oit27)1		243	253	182	206	146	174	nd	nd	nd	nd	nd	nd	176	210	188	188	150	174	206	206	confirmed
(Oit55 X Oit27)2		231	253	162	182	146	174	nd	nd	nd	nd	nd	nd	176	214	188	188	150	174	206	206	confirmed
(Oit55 X Oit27)3		243	253	162	206	146	150	nd	nd	nd	nd	nd	nd	186	214	188	194	160	174	194	206	confirmed
(Oit55 X Oit27)4		231	253	182	206	146	174	nd	nd	nd	nd	nd	nd	176	210	188	188	150	174	206	206	confirmed
(Oit55 X Oit27)5		231	253	182	206	146	174	nd	nd	nd	nd	nd	nd	176	210	188	188	150	174	206	206	confirmed
(Oit55 X Oit27)6		243	253	206	206	124	174	nd	nd	nd	nd	nd	nd	176	210	188	188	150	174	194	198	confirmed
(Oit55 X Oit27)7		243	253	182	206	124	174	nd	nd	nd	nd	nd	nd	176	214	188	188	150	186	194	198	confirmed
(Oit55 X Oit27)8		243	243	182	206	124	150	nd	nd	nd	nd	nd	nd	186	214	188	194	160	174	194	206	confirmed
(Oit55 X Oit27)9		243	253	162	182	146	174	nd	nd	nd	nd	nd	nd	176	210	188	190	150	174	206	206	confirmed
(Oit55 X Oit27)10		231	253	162	182	146	174	nd	nd	nd	nd	nd	nd	186	210	188	188	150	174	206	206	confirmed
(Oit55 X Oit27)11		231	253	162	206	124	150	nd	nd	nd	nd	nd	nd	186	210	188	188	150	174	206	206	confirmed
(Oit55 X Oit27)12		243	243	162	182	124	150	nd	nd	nd	nd	nd	nd	176	210	188	190	150	186	194	198	confirmed
**Oit28 x**	**G2**	239	253	162	184	124	126	173	177	199	219	122	130	172	212	188	190	134	134	206	206	
**Oit15**	**G2**	231	245	172	186	124	146	171	173	191	199	124	124	172	176	186	186	174	174	194	208	
(Oit28 x Oit15)1		239	253	162	184	126	126	173	177	199	199	130	130	172	172	190	190	134	134	206	206	self
(Oit28 x Oit15)2		253	253	162	184	124	124	177	177	199	219	122	130	172	212	188	190	134	134	206	206	self
**Oit57 x**	**G2**	245	253	162	206	154	174	173	181	199	219	124	144	210	214	188	190	136	150	204	206	
**Oit15**	**G2**	231	245	172	186	124	146	171	173	191	199	124	124	172	176	186	186	174	174	194	208	
(Oit57 x Oit15)1		245	245	162	162	154	174	173	181	219	219	144	144	210	210	188	188	136	136	206	206	self
(Oit57 x Oit15)2		245	245	162	162	154	154	173	181	219	219	124	144	210	210	188	190	136	136	206	206	self
**Oit57 x**	**G2**	245	253	162	206	154	174	173	181	199	219	124	144	210	214	188	190	136	150	204	206	
**Oit27**	**G1**	243	253	162	206	150	174	177	177	199	201	124	144	210	214	188,15	194,38	174	186	198	206	
(Oit57 x Oit27)1		253	253	206	206	150,11	154,28	173	177	199	199	124	144	210	210	188	194	150	174	204	206	confirmed
(Oit57 x Oit27)2		243	245	206	206	150,11	154,34	173	177	199	219	124	124	210	214	188	188	136	174	206	206	confirmed
(Oit57 x Oit27)3		0	0	206	206	150	173,64	177	181	201	219	124	124	214	214	188	194	150	186	204	206	confirmed
(Oit57 x Oit27)4		245	253	162	162	150,11	173,63	173	177	199	201	124	144	210	214	188	188	150	186	198	206	confirmed
(Oit57 x Oit27)5		245	253	162	206	150,11	154,34	173	177	201	219	124	144	214	214	188	194	150	186	206	206	confirmed
(Oit57 x Oit27)6		243	253	0	0	150	173,56	173	177	199	201	144	144	210	210	188	190	150	186	204	206	confirmed
(Oit57 x Oit27)7		243	253	162	206	150,11	173,63	173	177	201	219	124	144	210	214	188	190	136	186	206	206	confirmed
(Oit57 x Oit27)8		243	253	0	0	154	173,55	173	177	199	219	124	144	210	214	188	194	136	174	204	206	confirmed
(Oit57 x Oit27)9		245	253	162	206	154	173,66	177	181	199	201	124	144	210	210	188	188	150	186	198	206	confirmed
(Oit57 x Oit27)10		243	253	206	206	150	173,58	177	181	201	219	124	144	214	214	188	190	136	186	206	206	confirmed
(Oit57 x Oit27)11		253	253	206	206	150	154,23	177	181	0	0	124	144	214	214	188	194	136	174	198	204	confirmed
(Oit57 x Oit27)12		245	253	162	206	150	154,29	173	177	201	219	124	144	214	214	188	188	136	186	206	206	confirmed
(Oit57 x Oit27)13		253	253	162	206	154	173,63	173	177	199	201	124	124	210	214	190	194	150	186	206	206	confirmed
(Oit57 x Oit27)14		245	253	162	206	150	173,62	173	177	199	219	124	144	210	214	188	188	136	174	198	204	confirmed
(Oit57 x Oit27)15		245	253	162	162	150,11	154,29	177	181	199	219	144	144	210	214	190	194	136	174	204	206	confirmed
(Oit57 x Oit27)16		243	253	162	206	154	173,65	173	177	201	219	124	144	210	214	188	190	136	186	206	206	confirmed
(Oit57 x Oit27)17		245	253	162	206	150	154,23	177	181	199	219	124	124	210	214	188	194	136	174	204	206	confirmed
**Oit03 x**	**G2**	231	243	172	172	150	154	175	177	191	199	124	130	176	214	186	190	158	174	194	212	
**Oit27**	**G1**	243	253	162	206	150	174	177	177	199	201	124	144	210	214	188,15	194,38	174	186	198	206	
(Oit03 x Oit27)1		231	243	172	162	150	174	177	177	199	201	124	130	176	210	188	190	174	186	194	206	confirmed
(Oit03 x Oit27)2		243	243	0	0	150	150	177	177	199	199	0	0	176	214	186	194	158	186	198	212	confirmed
(Oit03 x Oit27)3		231	243	172	206	150	150	177	177	191	199	124	124	176	214	186	188	174	174	194	198	confirmed
(Oit03 x Oit27)4		231	243	172	206	150	154	177	177	191	201	124	144	210	214	190	194	0	0	194	206	confirmed
(Oit03 x Oit27)5		231	253	172	162	150	154	175	177	199	201	130	144	210	214	190	194	158	174	198	212	confirmed
(Oit03 x Oit27)6		243	243	172	162	150	150	175	177	199	201	124	144	176	214	188	190	174	186	206	212	confirmed
(Oit03 x Oit27)7		243	243	172	162	154	174	177	177	199	199	130	144	176	210	186	188	174	174	198	212	confirmed
(Oit03 x Oit27)8		231	243	172	162	150	154	175	177	0	0	124	130	214	214	190	194	158	174	206	212	confirmed
(Oit03 x Oit27)9		243	253	172	206	154	174	175	177	199	199	124	130	176	214	188	190	158	174	194	206	confirmed
(Oit03 x Oit27)10		231	243	172	206	150	174	177	177	199	199	124	130	214	214	186	194	0	0	194	198	confirmed
**Oit22 x**	**G2**	239	253	194	206	154	174	173	181	199	219	122	130	174	212	188	190	136	150	206	206	
**Oit27**	**G1**	243	253	162	206	150	174	177	177	199	201	124	144	210	214	188,15	194,38	174	186	198	206	
(Oit22 x Oit27)1		239	243	194	206	150	154	177	181	199	219	122	124	174	214	188	190	136	174	206	206	confirmed
(Oit22 x Oit27)2		0	0	162	206	154	174	177	181	199	199	124	130	210	212	188	194	136	174	0	0	confirmed
(Oit22 x Oit27)3		239	253	162	194	150	174	173	177	201	219	130	144	0	0	190	194	136	186	206	206	confirmed
(Oit22 x Oit27)4		253	253	206	206	174	174	173	177	199	201	124	130	212	214	188	188	150	174	206	206	confirmed
(Oit22 x Oit27)5		243	253	194	206	150	154	173	177	199	199	122	144	174	210	188	190	150	186	206	206	confirmed
(Oit22 x Oit27)6		253	253	0	0	174	174	177	181	199	219	122	144	174	210	188	188	150	186	206	206	confirmed
(Oit22 x Oit27)7		239	253	194	206	150	174	177	181	201	219	124	130	174	210	190	194	136	186	198	206	confirmed
(Oit22 x Oit27)8		243	253	206	206	154	174	0	0	199	199	124	130	212	214	188	188	0	0	206	206	confirmed
(Oit22 x Oit27)9		239	253	162	194	174	174	173	177	199	201	122	144	210	212	188	190	136	174	198	206	confirmed
(Oit22 x Oit27)10		243	253	194	206	150	174	177	181	199	201	130	144	174	214	188	194	150	186	206	206	confirmed
**Oit36 x**	**G2**	243	253	172	172	146	150	159	173	183	199	124	144	176	176	194	194	160	176	198	206	
**Oit27**	**G1**	243	253	162	206	150	174	177	177	199	201	124	144	210	214	188,15	194,38	174	186	198	206	
(Oit36 x Oit27)1		243	253	172	206	150	150	159	177	183	199	124	124	176	214	188	194	0	0	198	206	confirmed
(Oit36 x Oit27)2		243	243	172	206	146	150	173	177	199	199	124	144	176	214	194	194	0	0	198	206	confirmed
(Oit36 x Oit27)3		243	253	162	172	146	150	173	177	0	0	124	144	176	210	194	194	160	174	198	198	confirmed
(Oit36 x Oit27)4		253	253	162	172	150	174	159	177	183	199	124	144	176	214	188	194	160	174	0	0	confirmed
(Oit36 x Oit27)5		0	0	172	206	146	150	159	177	199	201	124	124	176	214	188	194	176	186	198	206	confirmed
(Oit36 x Oit27)6		0	0	162	172	150	150	173	177	199	201	124	144	176	210	194	194	176	186	198	206	confirmed
(Oit36 x Oit27)7		243	253	162	172	150	174	173	177	199	199	144	144	176	210	194	194	160	174	198	206	confirmed
(Oit36 x Oit27)8		243	243	172	206	146	150	0	0	199	199	144	144	176	210	194	194	176	186	206	206	confirmed
(Oit36 x Oit27)9		243	243	162	172	150	174	173	177	183	201	124	144	176	214	188	194	176	186	198	198	confirmed
(Oit36 x Oit27)10		243	253	162	172	146	174	159	177	183	201	124	144	176	210	188	194	0	0	198	206	confirmed
**LEDA-282 x**	**G2**	nd	nd	186	206	124	150	nd	nd	199	199	nd	nd	nd	nd	nd	nd	nd	nd	nd	nd	
**Oit27**	**G1**	243	253	162	206	150	174	177	177	199	201	124	144	210	214	188,15	194,38	174	186	198	206	
[(Oit27 x Oit15)282 x Oit27]1		nd	nd	186	206	123,7	150	nd	nd	199	199	nd	nd	nd	nd	nd	nd	nd	nd	nd	nd	confirmed
[(Oit27 x Oit15)282 x Oit27]2		nd	nd	162	206	123,74	150	nd	nd	199	199	nd	nd	nd	nd	nd	nd	nd	nd	nd	nd	confirmed
[(Oit27 x Oit15)282 x Oit27]3		nd	nd	162	162	123,77	174	nd	nd	199	201	nd	nd	nd	nd	nd	nd	nd	nd	nd	nd	confirmed
[(Oit27 x Oit15)282 x Oit27]4		nd	nd	162	186	123,65	174	nd	nd	199	199	nd	nd	nd	nd	nd	nd	nd	nd	nd	nd	confirmed
[(Oit27 x Oit15)282 x Oit27]5		nd	nd	186	206	123,77	150	nd	nd	0	0	nd	nd	nd	nd	nd	nd	nd	nd	nd	nd	confirmed
[(Oit27 x Oit15)282 x Oit27]6		nd	nd	206	206	150	174	nd	nd	199	201	nd	nd	nd	nd	nd	nd	nd	nd	nd	nd	confirmed
[(Oit27 x Oit15)282 x Oit27]7		nd	nd	206	206	123,77	150	nd	nd	199	199	nd	nd	nd	nd	nd	nd	nd	nd	nd	nd	confirmed
[(Oit27 x Oit15)282 x Oit27]8		nd	nd	206	206	150	150	nd	nd	199	201	nd	nd	nd	nd	nd	nd	nd	nd	nd	nd	confirmed
[(Oit27 x Oit15)282 x Oit27]9		nd	nd	206	206	123,75	150	nd	nd	199	201	nd	nd	nd	nd	nd	nd	nd	nd	nd	nd	confirmed
[(Oit27 x Oit15)282 x Oit27]10		nd	nd	206	206	123,67	174	nd	nd	199	201	nd	nd	nd	nd	nd	nd	nd	nd	nd	nd	confirmed
[(Oit27 x Oit15)282 x Oit27]11		nd	nd	162	186	150	174	nd	nd	199	199	nd	nd	nd	nd	nd	nd	nd	nd	nd	nd	confirmed
[(Oit27 x Oit15)282 x Oit27]12		nd	nd	162	186	150	150	nd	nd	199	199	nd	nd	nd	nd	nd	nd	nd	nd	nd	nd	confirmed
[(Oit27 x Oit15)282 x Oit27]13		nd	nd	162	186	123,69	150	nd	nd	199	201	nd	nd	nd	nd	nd	nd	nd	nd	nd	nd	confirmed
[(Oit27 x Oit15)282 x Oit27]14		nd	nd	186	206	123,66	150	nd	nd	199	201	nd	nd	nd	nd	nd	nd	nd	nd	nd	nd	confirmed
[(Oit27 x Oit15)282 x Oit27]15		nd	nd	162	206	123,73	150	nd	nd	199	199	nd	nd	nd	nd	nd	nd	nd	nd	nd	nd	confirmed
[(Oit27 x Oit15)282 x Oit27]16		nd	nd	186	206	123,85	174	nd	nd	199	201	nd	nd	nd	nd	nd	nd	nd	nd	nd	nd	confirmed
[(Oit27 x Oit15)282 x Oit27]17		nd	nd	162	186	123,62	150	nd	nd	199	199	nd	nd	nd	nd	nd	nd	nd	nd	nd	nd	confirmed
[(Oit27 x Oit15)282 x Oit27]18		nd	nd	186	206	123,62	150	nd	nd	199	199	nd	nd	nd	nd	nd	nd	nd	nd	nd	nd	confirmed
[(Oit27 x Oit15)282 x Oit27]19		nd	nd	162	206	123,66	174	nd	nd	199	201	nd	nd	nd	nd	nd	nd	nd	nd	nd	nd	confirmed
[(Oit27 x Oit15)282 x Oit27]20		nd	nd	186	206	123,73	174	nd	nd	199	201	nd	nd	nd	nd	nd	nd	nd	nd	nd	nd	confirmed
**LEDA-301 x**	**G2**	nd	nd	172	162	146	150	nd	nd	191	199	nd	nd	nd	nd	nd	nd	nd	nd	nd	nd	
**Oit27**	**G1**	243	253	162	206	150	174	177	177	199	201	124	144	210	214	188,15	194,38	174	186	198	206	
[(Oit27 x Oit15)301 x Oit27]1		nd	nd	162	172	150	174	nd	nd	199	201	nd	nd	nd	nd	nd	nd	nd	nd	nd	nd	confirmed
[(Oit27 x Oit15)301 x Oit27]2		nd	nd	162	172	150	150	nd	nd	191	201	nd	nd	nd	nd	nd	nd	nd	nd	nd	nd	confirmed
[(Oit27 x Oit15)301 x Oit27]3		nd	nd	162	172	146	150	nd	nd	191	201	nd	nd	nd	nd	nd	nd	nd	nd	nd	nd	confirmed
[(Oit27 x Oit15)301 x Oit27]4		nd	nd	162	172	150	150	nd	nd	199	201	nd	nd	nd	nd	nd	nd	nd	nd	nd	nd	confirmed
[(Oit27 x Oit15)301 x Oit27]5		nd	nd	162	162	150	174	nd	nd	199	199	nd	nd	nd	nd	nd	nd	nd	nd	nd	nd	confirmed
[(Oit27 x Oit15)301 x Oit27]6		nd	nd	162	172	146	150	nd	nd	199	201	nd	nd	nd	nd	nd	nd	nd	nd	nd	nd	confirmed
[(Oit27 x Oit15)301 x Oit27]7		nd	nd	162	172	150	150	nd	nd	199	199	nd	nd	nd	nd	nd	nd	nd	nd	nd	nd	confirmed
[(Oit27 x Oit15)301 x Oit27]8		nd	nd	162	206	150	150	nd	nd	191	199	nd	nd	nd	nd	nd	nd	nd	nd	nd	nd	confirmed
[(Oit27 x Oit15)301 x Oit27]9		nd	nd	162	162	146	150	nd	nd	199	199	nd	nd	nd	nd	nd	nd	nd	nd	nd	nd	confirmed
[(Oit27 x Oit15)301 x Oit27]10		nd	nd	172	206	146	174	nd	nd	199	199	nd	nd	nd	nd	nd	nd	nd	nd	nd	nd	confirmed
[(Oit27 x Oit15)301 x Oit27]11		nd	nd	172	206	146	174	nd	nd	191	199	nd	nd	nd	nd	nd	nd	nd	nd	nd	nd	confirmed
[(Oit27 x Oit15)301 x Oit27]12		nd	nd	162	162	146	174	nd	nd	199	199	nd	nd	nd	nd	nd	nd	nd	nd	nd	nd	confirmed
[(Oit27 x Oit15)301 x Oit27]13		nd	nd	162	162	150	174	nd	nd	191	201	nd	nd	nd	nd	nd	nd	nd	nd	nd	nd	confirmed
[(Oit27 x Oit15)301 x Oit27]14		nd	nd	162	172	146	174	nd	nd	199	199	nd	nd	nd	nd	nd	nd	nd	nd	nd	nd	confirmed
[(Oit27 x Oit15)301 x Oit27]15		nd	nd	172	206	146	174	nd	nd	199	201	nd	nd	nd	nd	nd	nd	nd	nd	nd	nd	confirmed
[(Oit27 x Oit15)301 x Oit27]16		nd	nd	172	206	150	174	nd	nd	191	199	nd	nd	nd	nd	nd	nd	nd	nd	nd	nd	confirmed
[(Oit27 x Oit15)301 x Oit27]17		nd	nd	162	172	146	150,11	nd	nd	199	201	nd	nd	nd	nd	nd	nd	nd	nd	nd	nd	confirmed
[(Oit27 x Oit15)301 x Oit27]18		nd	nd	162	172	146	174	nd	nd	191	199	nd	nd	nd	nd	nd	nd	nd	nd	nd	nd	confirmed
[(Oit27 x Oit15)301 x Oit27]19		nd	nd	172	206	150	174	nd	nd	191	199	nd	nd	nd	nd	nd	nd	nd	nd	nd	nd	confirmed
[(Oit27 x Oit15)301 x Oit27]20		nd	nd	162	172	150	174	nd	nd	199	201	nd	nd	nd	nd	nd	nd	nd	nd	nd	nd	confirmed
