# Supplementary material for: Elucidation of the genetic architecture of self‐incompatibility in olive: Evolutionary consequences and perspectives for orchard management
Source: Evol Appl. 2017 May 20;10(9):867–80. doi: 10.1111/eva.12457 (PMC5680433; doi:10.1111/eva.12457)
Supplement: Supplementary file 7 [file EVA-10-867-s007.pdf]

Table S4. Paternity assignments calculated with Cervus 3.0.3 on the genotypic data listed in Table S3.

Offspring ID	Loci typed	Mother ID	Loci typed	Pair loci compared	Pair loci mismatching	Pair LOD score	Candidate father ID	Loci typed	Pair loci compared	Pair loci mismatching	Pair LOD score	Pair Delta	Pair confidence	Trio loci compared	Trio loci mismatching	Trio LOD score	Trio Delta	Trio confidence
(Oit55 X Oit15)1	10	Oit55	10	10	0	1,18E+01	Oit55	10	10	0	1,18E+01	0,00E+00		10	0	9,87E+00	9,87E+00	*
(Oit55 X Oit15)2	10	Oit55	10	10	0	1,18E+01	Oit55	10	10	0	1,18E+01	0,00E+00		10	0	9,02E+00	9,02E+00	*
(Oit55 X Oit15)3	10	Oit55	10	10	0	9,87E+00	Oit55	10	10	0	9,87E+00	0,00E+00		10	0	8,00E+00	8,00E+00	*
(Oit55 X Oit15)4	10	Oit55	10	10	0	1,27E+01	Oit55	10	10	0	1,27E+01	0,00E+00		10	0	1,10E+01	1,10E+01	*
(Oit55 X Oit15)5	10	Oit55	10	10	0	8,62E+00	Oit55	10	10	0	8,62E+00	0,00E+00		10	0	8,18E+00	8,18E+00	*
(Oit55 X Oit15)6	10	Oit55	10	10	0	1,04E+01	Oit55	10	10	0	1,04E+01	0,00E+00		10	0	7,90E+00	7,90E+00	*
(Oit55 X Oit15)7	10	Oit55	10	10	0	1,11E+01	Oit55	10	10	0	1,11E+01	0,00E+00		10	0	9,01E+00	9,01E+00	*
(Oit55 X Oit15)8	10	Oit55	10	10	0	9,56E+00	Oit55	10	10	0	9,56E+00	0,00E+00		10	0	9,53E+00	9,53E+00	*
(Oit55 X Oit15)9	10	Oit55	10	10	0	7,87E+00	Oit55	10	10	0	7,87E+00	0,00E+00		10	0	7,60E+00	7,60E+00	*
(Oit55 X Oit15)10	10	Oit55	10	10	0	1,18E+01	Oit55	10	10	0	1,18E+01	0,00E+00		10	0	8,92E+00	8,92E+00	*
(Oit28 x Oit15)1	10	Oit28	10	10	0	1,95E+01	Oit28	10	10	0	1,95E+01	0,00E+00		10	0	1,84E+01	1,84E+01	*
(Oit28 x Oit15)2	10	Oit28	10	10	0	1,65E+01	Oit28	10	10	0	1,65E+01	0,00E+00		10	0	1,49E+01	1,49E+01	*
(Oit57 x Oit15)1	10	Oit57	10	10	0	1,41E+01	Oit57	10	10	0	1,41E+01	0,00E+00		10	0	1,40E+01	1,40E+01	*
(Oit57 x Oit15)2	10	Oit57	10	10	0	1,48E+01	Oit57	10	10	0	1,48E+01	0,00E+00		10	0	1,44E+01	1,44E+01	*
(Oit57 x Oit27)1	10	Oit57	10	10	0	7,28E+00	Oit27	10	10	0	3,21E+00	3,21E+00	*	10	0	6,01E+00	6,01E+00	*
(Oit57 x Oit27)2	10	Oit57	10	10	0	8,62E+00	Oit27	10	10	0	1,13E+00	1,13E+00	*	10	0	4,36E+00	1,38E+00	*
(Oit57 x Oit27)3	9	Oit57	10	9	0	6,98E+00	Oit27	10	9	0	4,34E+00	4,34E+00	*	9	0	6,12E+00	6,12E+00	*
(Oit57 x Oit27)4	10	Oit57	10	10	0	5,97E+00	Oit27	10	10	0	8,16E+00	8,16E+00	*	10	0	9,74E+00	9,74E+00	*
(Oit57 x Oit27)5	10	Oit57	10	10	0	7,18E+00	Oit27	10	10	0	4,90E+00	4,90E+00	*	10	0	7,77E+00	7,77E+00	*
(Oit57 x Oit27)6	9	Oit57	10	9	0	7,08E+00	Oit27	10	9	0	4,98E+00	4,98E+00	*	9	0	6,20E+00	6,20E+00	*
(Oit57 x Oit27)7	10	Oit57	10	10	0	8,69E+00	Oit27	10	10	0	6,25E+00	6,25E+00	*	10	0	6,63E+00	6,63E+00	*
(Oit57 x Oit27)8	9	Oit57	10	9	0	9,76E+00	Oit27	10	9	0	3,43E+00	3,43E+00	*	9	0	5,64E+00	5,64E+00	*
(Oit57 x Oit27)9	10	Oit57	10	10	0	7,08E+00	Oit27	10	10	0	7,61E+00	7,61E+00	*	10	0	9,92E+00	9,92E+00	*
(Oit57 x Oit27)10	10	Oit57	10	10	0	8,27E+00	Oit27	10	10	0	5,18E+00	5,18E+00	*	10	0	6,04E+00	6,04E+00	*
(Oit57 x Oit27)11	9	Oit57	10	9	0	8,97E+00	Oit27	10	9	0	3,70E+00	3,70E+00	*	9	0	6,58E+00	6,21E+00	*
(Oit57 x Oit27)12	10	Oit57	10	10	0	9,68E+00	Oit27	10	10	0	4,71E+00	4,71E+00	*	10	0	7,44E+00	7,44E+00	*
(Oit57 x Oit27)13	10	Oit57	10	10	0	6,32E+00	Oit27	10	10	0	6,83E+00	6,83E+00	*	10	0	8,93E+00	8,93E+00	*
(Oit57 x Oit27)14	10	Oit57	10	10	0	1,24E+01	Oit27	10	10	0	5,50E+00	5,50E+00	*	10	0	6,97E+00	6,97E+00	*
(Oit57 x Oit27)15	10	Oit57	10	10	0	1,45E+01	Oit27	10	10	0	2,81E+00	2,81E+00	*	10	0	7,16E+00	7,16E+00	*
(Oit57 x Oit27)16	10	Oit57	10	10	0	9,50E+00	Oit27	10	10	0	6,07E+00	6,07E+00	*	10	0	7,64E+00	7,64E+00	*
(Oit57 x Oit27)17	10	Oit57	10	10	0	1,24E+01	Oit27	10	10	0	2,47E+00	2,47E+00	*	10	0	5,82E+00	5,82E+00	*
(Oit03 x Oit27)1	10	Oit03	10	10	0	4,87E+00	Oit27	10	10	0	4,11E+00	4,11E+00	*	10	0	8,27E+00	8,27E+00	*
(Oit03 x Oit27)2	8	Oit03	10	8	0	1,06E+01	Oit27	10	8	0	3,38E+00	3,38E+00	*	8	0	5,22E+00	5,22E+00	*
(Oit03 x Oit27)3	10	Oit03	10	10	0	1,22E+01	Oit27	10	10	0	1,61E+00	0,00E+00		10	0	4,21E+00	6,60E-03	*
(Oit03 x Oit27)4	9	Oit03	10	9	0	9,13E+00	Oit27	10	9	0	1,09E+00	1,09E+00	*	9	0	4,78E+00	4,78E+00	*
(Oit03 x Oit27)5	10	Oit03	10	10	0	1,24E+01	Oit27	10	10	0	4,09E+00	4,09E+00	*	10	0	9,82E+00	9,82E+00	*
(Oit03 x Oit27)6	10	Oit03	10	10	0	9,03E+00	Oit27	10	10	0	3,68E+00	3,68E+00	*	10	0	6,92E+00	6,92E+00	*
(Oit03 x Oit27)7	10	Oit03	10	10	0	1,09E+01	Oit27	10	10	0	5,21E+00	5,21E+00	*	10	0	9,32E+00	9,32E+00	*
(Oit03 x Oit27)8	9	Oit03	10	9	0	1,43E+01	Oit27	10	9	0	2,16E-01	2,16E-01	*	9	0	4,34E+00	4,34E+00	*
(Oit03 x Oit27)9	10	Oit03	10	10	0	1,22E+01	Oit27	10	10	0	1,10E+00	1,10E+00	*	10	0	5,30E+00	5,30E+00	*
(Oit03 x Oit27)10	9	Oit03	10	9	0	8,40E+00	Oit27	10	9	0	2,65E+00	2,45E+00	*	9	0	5,50E+00	5,50E+00	*
(Oit22 x Oit27)1	10	Oit22	10	10	0	1,53E+01	Oit27	10	10	0	-1,71E+00	0,00E+00		10	0	3,83E+00	6,90E-01	*
(Oit22 x Oit27)2	8	Oit22	10	8	0	7,13E+00	Oit27	10	8	0	3,95E+00	0,00E+00		8	0	7,43E+00	7,43E+00	*
(Oit22 x Oit27)3	9	Oit22	10	9	0	9,66E+00	Oit27	10	9	0	4,64E+00	0,00E+00		9	0	8,23E+00	8,23E+00	*
(Oit22 x Oit27)4	10	Oit22	10	10	0	6,75E+00	Oit27	10	10	0	4,87E+00	1,62E+00	*	10	0	8,02E+00	8,02E+00	*
(Oit22 x Oit27)5	10	Oit22	10	10	0	9,98E+00	Oit27	10	10	0	1,33E+00	0,00E+00		10	0	5,16E+00	5,16E+00	*
(Oit22 x Oit27)6	9	Oit22	10	9	0	1,07E+01	Oit27	10	9	0	4,40E+00	0,00E+00		9	0	7,66E+00	7,66E+00	*
(Oit22 x Oit27)7	10	Oit22	10	10	0	1,25E+01	Oit27	10	10	0	3,99E+00	0,00E+00		10	0	8,54E+00	8,54E+00	*
(Oit22 x Oit27)8	8	Oit22	10	8	0	6,02E+00	Oit27	10	8	0	2,04E+00	0,00E+00		8	0	2,99E+00	2,99E+00	*
(Oit22 x Oit27)9	10	Oit22	10	10	0	1,25E+01	Oit27	10	10	0	5,68E+00	0,00E+00		10	0	1,09E+01	1,09E+01	*
(Oit22 x Oit27)10	10	Oit22	10	10	0	6,72E+00	Oit27	10	10	0	4,45E+00	2,89E+00	*	10	0	6,66E+00	6,66E+00	*
(Oit36 x Oit27)1	9	Oit36	10	9	0	8,35E+00	Oit27	10	9	0	2,17E+00	4,27E-01	*	9	0	3,69E+00	6,88E-01	*
(Oit36 x Oit27)2	9	Oit36	10	9	0	7,13E+00	Oit27	10	9	0	2,00E+00	1,96E-01	*	9	0	3,96E+00	6,88E-01	*
(Oit36 x Oit27)3	9	Oit36	10	9	0	8,36E+00	Oit27	10	9	0	4,48E+00	4,48E+00	*	9	0	7,33E+00	7,33E+00	*
(Oit36 x Oit27)4	9	Oit36	10	9	0	8,47E+00	Oit27	10	9	0	5,07E+00	5,07E+00	*	9	0	8,34E+00	8,34E+00	*
(Oit36 x Oit27)5	9	Oit36	10	9	0	9,94E+00	Oit27	10	9	0	2,99E+00	2,99E+00	*	9	0	5,89E+00	5,89E+00	*
(Oit36 x Oit27)6	9	Oit36	10	9	0	8,60E+00	Oit27	10	9	0	5,68E+00	5,68E+00	*	9	0	8,44E+00	8,44E+00	*
(Oit36 x Oit27)7	10	Oit36	10	10	0	7,46E+00	Oit27	10	10	0	6,50E+00	6,50E+00	*	10	0	9,19E+00	9,19E+00	*
(Oit36 x Oit27)8	9	Oit36	10	9	0	8,20E+00	Oit27	10	9	0	1,82E+00	1,82E+00	*	9	0	4,20E+00	4,20E+00	*
(Oit36 x Oit27)9	10	Oit36	10	10	0	8,50E+00	Oit27	10	10	0	6,83E+00	6,83E+00	*	10	0	1,06E+01	1,06E+01	*
(Oit36 x Oit27)10	9	Oit36	10	9	0	8,22E+00	Oit27	10	9	0	6,05E+00	6,05E+00	*	9	0	8,70E+00	8,70E+00	*
