# Supplementary material for: Elucidation of the genetic architecture of self‐incompatibility in olive: Evolutionary consequences and perspectives for orchard management
Source: Evol Appl. 2017 May 20;10(9):867–80. doi: 10.1111/eva.12457 (PMC5680433; doi:10.1111/eva.12457)
Supplement: Supplementary file 8 [file EVA-10-867-s008.pdf]

Table S5-A. Parameters of genetic diversity of the complete collection of olive trees (total of 342 genotypes) and the sampled set of trees used in stigma tests in the present study (89 genotypes used for phenotyping self-incompatibility).

**Locus**	**Total (342 genotypes)**				**Phenotyped (89 genotypes)**			
	**Na**	**Size alleles***	**Ho**	**He**	**Na**	**Size alleles***	**Ho**	**He**
ssrOeUA-DCA1	21	203-273	0.725	0.633	14	203-271	0.786	0.664
ssrOeUA-DCA3	14	227-263	0.891	0.853	11	227-252	0.910	0.846
ssrOeUA-DCA4	36	116-198	0.649	0.859	22	128-192	0.618	0.858
ssrOeUA-DCA5	12	190-212	0.52	0.518	11	190-212	0.528	0.508
ssrOeUA-DCA8	20	123-164	0.932	0.83	15	123-152	0.898	0.808
ssrOeUA-DCA9	25	159-217	0.953	0.886	20	159-207	0.943	0.885
ssrOeUA-DCA11	25	125-199	0.842	0.828	18	125-181	0.865	0.832
ssrOeUA-DCA14	12	167-190	0.739	0.705	9	169-186	0.775	0.727
ssrOeUA-DCA15	9	242-265	0.698	0.654	6	242-265	0.764	0.673
ssrOeUA-DCA18	16	154-188	0.926	0.847	11	158-180	0.955	0.844
EMO03	14	201-215	0.818	0.807	10	202-215	0.853	0.809
EMO90	9	181-208	0.675	0.659	6	181-197	0.64	0.594
GAPU59	10	206-239	0.59	0.617	8	206-239	0.55	0.602
GAPU 71B	10	116-144	0.903	0.807	8	116-144	0.91	0.791
UDO36	12	138-166	0.683	0.728	10	138-164	0.696	0.727
**Total**	**245**				**179**			
**Mean**	**16.33**		**0.77**	**0.749**	**11.93**		**0.779**	**0.745**

Total: the 309 genotypes present in the OWGB collection of Marrakech and the 33 genotypes sampled in other collections (Italian and French collections) for the present study but absent from the OWGB collection. Phenotyped: the 89 genotypes phenotyped for SI group in the present study (Table S1); * in base pairs (bp); Na: number of allele scored; Ho: the observed heterozygosity; He: the expected heterozygosity. Na, Ho, and He were calculated using the excel microsatellite toolkit v3.1 (Park, 2001).
