# Supplementary material for: Elucidation of the genetic architecture of self‐incompatibility in olive: Evolutionary consequences and perspectives for orchard management
Source: Evol Appl. 2017 May 20;10(9):867–80. doi: 10.1111/eva.12457 (PMC5680433; doi:10.1111/eva.12457)
Supplement: Supplementary file 9 [file EVA-10-867-s009.pdf]

Table S5-B. Comparison of allelic diversity between the total of 342 genotypes and the 89 genotypes used for self-incompatibility phenotyping.

	Na[1]	Na[2]	Ar[3]
ssrOeUA-DCA1	21	**14**	**8.73**
ssrOeUA-DCA3	14	**11**	**9.1**
ssrOeUA-DCA4	36	**22**	**18.01**
ssrOeUA-DCA5	12	**11**	**9.32**
ssrOeUA-DCA8	20	**15**	**12.67**
ssrOeUA-DCA9	25	**20**	**16.24**
ssrOeUA-DCA11	25	**18**	**14.27**
ssrOeUA-DCA14	12	**9**	**8.023**
ssrOeUA-DCA15	9	**6**	**5.91**
ssrOeUA-DCA18	16	**11**	**11.57**
EMO03	14	**10**	**9.38**
EMO90	9	**6**	**6.69**
GAPU59	10	**8**	**6.65**
GAPU71B	10	**8**	**6.97**
UDO36	12	**10**	**8.64**
**Total**	**245**	**179**	**152.25**
**mean**	**16.33**	**11.93**	**10.15**

The pairwise values Na[2] / Ar is not significantly different at p ≤0.01 using one tailed Mann-Whitney's U test (U=89; *P*-value = 0.171).

[1] number of alleles detected in 342 genotypes
[2] number of alleles detected in 89 genotypes used for self-incompatibility phenotyping
[3] allelic richness standardised at value G = 89 individuals computed by ADZE software (Szpiech et al. 2008).

Reference:

Szpiech, Z.A., M. Jakobsson, and N.A. Rosenberg. 2008. ADZE: a rarefaction approach for counting alleles private to combinations of populations. *Bioinformatics* 24 (21):2498-2504.
