# Supplementary material for: Elucidation of the genetic architecture of self‐incompatibility in olive: Evolutionary consequences and perspectives for orchard management
Source: Evol Appl. 2017 May 20;10(9):867–80. doi: 10.1111/eva.12457 (PMC5680433; doi:10.1111/eva.12457)
Supplement: Supplementary file 10 [file EVA-10-867-s010.pdf]

Table S5-C. Distribution of the 342 Mediterranean genotypes representing our collection, and the 89 genotypes phenotyped for self-incompatibility (SI), into the three gene pools (western, central and eastern Mediterranean) and the admixed group.

Except for the eastern gene pool, from which only nine genotypes were phenotyped for SI, we noted roughly similar proportions in western, central and admixed groups, indicating the representativeness of SI sampling of domesticated Mediterranean olive diversity. q: the shared ancestry value.

	West	Centre	East	Admixed*	**Total**
Total of 342 genotypes	72 (21.1%)	85 (24.8%)	66 (19.3%)	119 (34.8%)	**342**
The 89 genotypes**	21 (23.6%)	23 (25.8%)	9 (10.1%)	36 (40.5%)	**89**

* q < 0.8

** used for self-incompatibility phenotyping
