# Supplementary material for: Elucidation of the genetic architecture of self‐incompatibility in olive: Evolutionary consequences and perspectives for orchard management
Source: Evol Appl. 2017 May 20;10(9):867–80. doi: 10.1111/eva.12457 (PMC5680433; doi:10.1111/eva.12457)
Supplement: Supplementary file 11 [file EVA-10-867-s011.pdf]

Table S6. Scoring of the pollen-tube length variations in incompatible crosses.

A subset of 86 pollen donors was crossed with the four *O. europaea* testers involved in the stigma test (Oit15, Oit26, Oit27, and Oit30). For each pollen donor, we observed four crosses (two compatible and two incompatible) and for each cross we photographed three flowers separately. The images of incompatible cross were randomly labeled and observed four times independently, providing for each flower four independent flower reads to assign a phenotypic class (i.e. 12 independent scores for each cross).

Pollen Recepient reference	SI Group of pollen Recipient	Pollen donor reference	SI Group of pollen donor	Pollination date	Photo reference number	photo random number	N° of flower	Repetition of flower read	Class of SI phenotype
Oit30	G2	LEDA_092	G2	30/05/14	584	175	1	A	1
Oit30	G2	LEDA_092	G2	30/05/14	584	175	1	B	1
Oit30	G2	LEDA_092	G2	30/05/14	584	175	1	C	2
Oit30	G2	LEDA_092	G2	30/05/14	584	175	1	D	2
Oit30	G2	LEDA_092	G2	30/05/14	585	194	2	A	2
Oit30	G2	LEDA_092	G2	30/05/14	585	194	2	B	3
Oit30	G2	LEDA_092	G2	30/05/14	585	194	2	C	3
Oit30	G2	LEDA_092	G2	30/05/14	585	194	2	D	3
Oit30	G2	LEDA_092	G2	01/06/14	1362	426	1	A	2
Oit30	G2	LEDA_092	G2	01/06/14	1362	426	1	B	2
Oit30	G2	LEDA_092	G2	01/06/14	1362	426	1	C	2
Oit30	G2	LEDA_092	G2	01/06/14	1362	426	1	D	2
Oit30	G2	LEDA_092	G2	01/06/14	1363	414	2	A	2
Oit30	G2	LEDA_092	G2	01/06/14	1363	414	2	B	3
Oit30	G2	LEDA_092	G2	01/06/14	1363	414	2	C	3
Oit30	G2	LEDA_092	G2	01/06/14	1363	414	2	D	3
Oit30	G2	LEDA_092	G2	01/06/14	1364	430	3	A	2
Oit30	G2	LEDA_092	G2	01/06/14	1364	430	3	B	2
Oit30	G2	LEDA_092	G2	01/06/14	1364	430	3	C	2
Oit30	G2	LEDA_092	G2	01/06/14	1364	430	3	D	2
Oit15	G2	LEDA_092	G2	30/05/14	578	165	1	A	2
Oit15	G2	LEDA_092	G2	30/05/14	578	165	1	B	2
Oit15	G2	LEDA_092	G2	30/05/14	578	165	1	C	2
Oit15	G2	LEDA_092	G2	30/05/14	578	165	1	D	2
Oit15	G2	LEDA_092	G2	30/05/14	579	130	2	A	2
Oit15	G2	LEDA_092	G2	30/05/14	579	130	2	B	2
Oit15	G2	LEDA_092	G2	30/05/14	579	130	2	C	3
Oit15	G2	LEDA_092	G2	30/05/14	579	130	2	D	3
Oit15	G2	LEDA_092	G2	30/05/14	580	133	3	A	2
Oit15	G2	LEDA_092	G2	30/05/14	580	133	3	B	3
Oit15	G2	LEDA_092	G2	30/05/14	580	133	3	C	3
Oit15	G2	LEDA_092	G2	30/05/14	580	133	3	D	3
Oit30	G2	LEDA_222	G2	30/05/14	592	87	1	A	3
Oit30	G2	LEDA_222	G2	30/05/14	592	87	1	B	3
Oit30	G2	LEDA_222	G2	30/05/14	592	87	1	C	4
Oit30	G2	LEDA_222	G2	30/05/14	592	87	1	D	3
Oit30	G2	LEDA_222	G2	30/05/14	593	64	2	A	2
Oit30	G2	LEDA_222	G2	30/05/14	593	64	2	B	2
Oit30	G2	LEDA_222	G2	30/05/14	593	64	2	C	3
Oit30	G2	LEDA_222	G2	30/05/14	593	64	2	D	3
Oit15	G2	LEDA_222	G2	30/05/14	588	59	1	A	3
Oit15	G2	LEDA_222	G2	30/05/14	588	59	1	B	3
Oit15	G2	LEDA_222	G2	30/05/14	588	59	1	C	3
Oit15	G2	LEDA_222	G2	30/05/14	588	59	1	D	3
Oit15	G2	LEDA_222	G2	30/05/14	589	120	2	A	3
Oit15	G2	LEDA_222	G2	30/05/14	589	120	2	B	4
Oit15	G2	LEDA_222	G2	30/05/14	589	120	2	C	4
Oit15	G2	LEDA_222	G2	30/05/14	589	120	2	D	4
Oit30	G2	LEDA_262	G2	30/05/14	601	155	1	A	2
Oit30	G2	LEDA_262	G2	30/05/14	601	155	1	B	3
Oit30	G2	LEDA_262	G2	30/05/14	601	155	1	C	4
Oit30	G2	LEDA_262	G2	30/05/14	601	155	1	D	3

Pollen Recepient reference	SI Group of pollen Recipient	Pollen donor reference	SI Group of pollen donor	Pollination date	Photo reference number	photo random number	N° of flower	Repetition of flower read	Class of SI phenotype
Oit30	G2	LEDA_262	G2	30/05/14	602	32	2	A	2
Oit30	G2	LEDA_262	G2	30/05/14	602	32	2	B	2
Oit30	G2	LEDA_262	G2	30/05/14	602	32	2	C	2
Oit30	G2	LEDA_262	G2	30/05/14	602	32	2	D	2
Oit15	G2	LEDA_262	G2	30/05/14	596	80	1	A	2
Oit15	G2	LEDA_262	G2	30/05/14	596	80	1	B	2
Oit15	G2	LEDA_262	G2	30/05/14	596	80	1	C	2
Oit15	G2	LEDA_262	G2	30/05/14	596	80	1	D	2
Oit15	G2	LEDA_262	G2	30/05/14	597	122	2	A	2
Oit15	G2	LEDA_262	G2	30/05/14	597	122	2	B	2
Oit15	G2	LEDA_262	G2	30/05/14	597	122	2	C	3
Oit15	G2	LEDA_262	G2	30/05/14	597	122	2	D	2
Oit15	G2	LEDA_262	G2	30/05/14	598	111	3	A	2
Oit15	G2	LEDA_262	G2	30/05/14	598	111	3	B	2
Oit15	G2	LEDA_262	G2	30/05/14	598	111	3	C	3
Oit15	G2	LEDA_262	G2	30/05/14	598	111	3	D	3
Oit30	G2	LEDA_282	G2	30/05/14	610	195	1	A	1
Oit30	G2	LEDA_282	G2	30/05/14	610	195	1	B	2
Oit30	G2	LEDA_282	G2	30/05/14	610	195	1	C	2
Oit30	G2	LEDA_282	G2	30/05/14	610	195	1	D	2
Oit30	G2	LEDA_282	G2	30/05/14	611	45	2	A	1
Oit30	G2	LEDA_282	G2	30/05/14	611	45	2	B	1
Oit30	G2	LEDA_282	G2	30/05/14	611	45	2	C	0
Oit30	G2	LEDA_282	G2	30/05/14	611	45	2	D	0
Oit30	G2	LEDA_282	G2	30/05/14	612	126	3	A	2
Oit30	G2	LEDA_282	G2	30/05/14	612	126	3	B	2
Oit30	G2	LEDA_282	G2	30/05/14	612	126	3	C	3
Oit30	G2	LEDA_282	G2	30/05/14	612	126	3	D	2
Oit15	G2	LEDA_282	G2	30/05/14	606	91	1	A	2
Oit15	G2	LEDA_282	G2	30/05/14	606	91	1	B	3
Oit15	G2	LEDA_282	G2	30/05/14	606	91	1	C	3
Oit15	G2	LEDA_282	G2	30/05/14	606	91	1	D	3
Oit15	G2	LEDA_282	G2	30/05/14	607	38	2	A	5
Oit15	G2	LEDA_282	G2	30/05/14	607	38	2	B	5
Oit15	G2	LEDA_282	G2	30/05/14	607	38	2	C	4
Oit15	G2	LEDA_282	G2	30/05/14	607	38	2	D	4
Oit30	G2	LEDA_301	G2	30/05/14	620	37	1	A	2
Oit30	G2	LEDA_301	G2	30/05/14	620	37	1	B	2
Oit30	G2	LEDA_301	G2	30/05/14	620	37	1	C	2
Oit30	G2	LEDA_301	G2	30/05/14	620	37	1	D	3
Oit30	G2	LEDA_301	G2	30/05/14	621	63	2	A	3
Oit30	G2	LEDA_301	G2	30/05/14	621	63	2	B	3
Oit30	G2	LEDA_301	G2	30/05/14	621	63	2	C	4
Oit30	G2	LEDA_301	G2	30/05/14	621	63	2	D	4
Oit15	G2	LEDA_301	G2	30/05/14	616	7	1	A	5
Oit15	G2	LEDA_301	G2	30/05/14	616	7	1	B	3
Oit15	G2	LEDA_301	G2	30/05/14	616	7	1	C	4
Oit15	G2	LEDA_301	G2	30/05/14	616	7	1	D	4
Oit15	G2	LEDA_301	G2	30/05/14	617	167	2	A	3
Oit15	G2	LEDA_301	G2	30/05/14	617	167	2	B	3
Oit15	G2	LEDA_301	G2	30/05/14	617	167	2	C	3
Oit15	G2	LEDA_301	G2	30/05/14	617	167	2	D	4
Oit30	G2	LEDA_311	G2	01/06/14	1412	428	1	A	1
Oit30	G2	LEDA_311	G2	01/06/14	1412	428	1	B	2
Oit30	G2	LEDA_311	G2	01/06/14	1412	428	1	C	2
Oit30	G2	LEDA_311	G2	01/06/14	1412	428	1	D	2
Oit30	G2	LEDA_311	G2	01/06/14	1413	420	2	A	2
Oit30	G2	LEDA_311	G2	01/06/14	1413	420	2	B	2

Pollen Recepient reference	SI Group of pollen Recipient	Pollen donor reference	SI Group of pollen donor	Pollination date	Photo reference number	photo random number	N° of flower	Repetition of flower read	Class of SI phenotype
Oit30	G2	LEDA_311	G2	01/06/14	1413	420	2	C	3
Oit30	G2	LEDA_311	G2	01/06/14	1413	420	2	D	2
Oit15	G2	LEDA_311	G2	29/05/14	1005	224	1	A	3
Oit15	G2	LEDA_311	G2	29/05/14	1005	224	1	B	4
Oit15	G2	LEDA_311	G2	29/05/14	1005	224	1	C	4
Oit15	G2	LEDA_311	G2	29/05/14	1005	224	1	D	4
Oit15	G2	LEDA_311	G2	29/05/14	1006	231	2	A	3
Oit15	G2	LEDA_311	G2	29/05/14	1006	231	2	B	3
Oit15	G2	LEDA_311	G2	29/05/14	1006	231	2	C	4
Oit15	G2	LEDA_311	G2	29/05/14	1006	231	2	D	4
Oit15	G2	LEDA_311	G2	29/05/14	1007	205	3	A	6
Oit15	G2	LEDA_311	G2	29/05/14	1007	205	3	B	8
Oit15	G2	LEDA_311	G2	29/05/14	1007	205	3	C	8
Oit15	G2	LEDA_311	G2	29/05/14	1007	205	3	D	8
Oit30	G2	Oct_002	G2	31/05/14	1293	302	1	A	1
Oit30	G2	Oct_002	G2	31/05/14	1293	302	1	B	1
Oit30	G2	Oct_002	G2	31/05/14	1293	302	1	C	2
Oit30	G2	Oct_002	G2	31/05/14	1293	302	1	D	2
Oit30	G2	Oct_002	G2	31/05/14	1294	399	2	A	2
Oit30	G2	Oct_002	G2	31/05/14	1294	399	2	B	2
Oit30	G2	Oct_002	G2	31/05/14	1294	399	2	C	2
Oit30	G2	Oct_002	G2	31/05/14	1294	399	2	D	2
Oit30	G2	Oct_002	G2	31/05/14	1295	324	3	A	1
Oit30	G2	Oct_002	G2	31/05/14	1295	324	3	B	1
Oit30	G2	Oct_002	G2	31/05/14	1295	324	3	C	2
Oit30	G2	Oct_002	G2	31/05/14	1295	324	3	D	2
Oit15	G2	Oct_002	G2	29/05/14	880	292	1	A	2
Oit15	G2	Oct_002	G2	29/05/14	880	292	1	B	3
Oit15	G2	Oct_002	G2	29/05/14	880	292	1	C	3
Oit15	G2	Oct_002	G2	29/05/14	880	292	1	D	3
Oit15	G2	Oct_002	G2	29/05/14	881	267	2	A	4
Oit15	G2	Oct_002	G2	29/05/14	881	267	2	B	5
Oit15	G2	Oct_002	G2	29/05/14	881	267	2	C	4
Oit15	G2	Oct_002	G2	29/05/14	881	267	2	D	5
Oit15	G2	Oct_002	G2	29/05/14	882	283	3	A	1
Oit15	G2	Oct_002	G2	29/05/14	882	283	3	B	2
Oit15	G2	Oct_002	G2	29/05/14	882	283	3	C	2
Oit15	G2	Oct_002	G2	29/05/14	882	283	3	D	2
Oit30	G2	Oct_005	G2	31/05/14	1140	313	1	A	0
Oit30	G2	Oct_005	G2	31/05/14	1140	313	1	B	0
Oit30	G2	Oct_005	G2	31/05/14	1140	313	1	C	0
Oit30	G2	Oct_005	G2	31/05/14	1140	313	1	D	0
Oit30	G2	Oct_005	G2	31/05/14	1141	363	2	A	1
Oit30	G2	Oct_005	G2	31/05/14	1141	363	2	B	0
Oit30	G2	Oct_005	G2	31/05/14	1141	363	2	C	0
Oit30	G2	Oct_005	G2	31/05/14	1141	363	2	D	0
Oit15	G2	Oct_005	G2	27/05/14	153	72	1	A	0
Oit15	G2	Oct_005	G2	27/05/14	153	72	1	B	0
Oit15	G2	Oct_005	G2	27/05/14	153	72	1	C	0
Oit15	G2	Oct_005	G2	27/05/14	153	72	1	D	0
Oit15	G2	Oct_005	G2	27/05/14	154	157	2	A	0
Oit15	G2	Oct_005	G2	27/05/14	154	157	2	B	0
Oit15	G2	Oct_005	G2	27/05/14	154	157	2	C	0
Oit15	G2	Oct_005	G2	27/05/14	154	157	2	D	0
Oit27	G1	Oct_008	G1	31/05/14	1244	333	1	A	1
Oit27	G1	Oct_008	G1	31/05/14	1244	333	1	B	1
Oit27	G1	Oct_008	G1	31/05/14	1244	333	1	C	1
Oit27	G1	Oct_008	G1	31/05/14	1244	333	1	D	0

Pollen Recepient reference	SI Group of pollen Recipient	Pollen donor reference	SI Group of pollen donor	Pollination date	Photo reference number	photo random number	N° of flower	Repetition of flower read	Class of SI phenotype
Oit30	G2	Oct_013	G2	31/05/14	1315	395	1	A	2
Oit30	G2	Oct_013	G2	31/05/14	1315	395	1	B	2
Oit30	G2	Oct_013	G2	31/05/14	1315	395	1	C	2
Oit30	G2	Oct_013	G2	31/05/14	1315	395	1	D	3
Oit30	G2	Oct_013	G2	31/05/14	1316	314	2	A	2
Oit30	G2	Oct_013	G2	31/05/14	1316	314	2	B	3
Oit30	G2	Oct_013	G2	31/05/14	1316	314	2	C	3
Oit30	G2	Oct_013	G2	31/05/14	1316	314	2	D	3
Oit30	G2	Oct_013	G2	31/05/14	1317	342	3	A	2
Oit30	G2	Oct_013	G2	31/05/14	1317	342	3	B	4
Oit30	G2	Oct_013	G2	31/05/14	1317	342	3	C	4
Oit30	G2	Oct_013	G2	31/05/14	1317	342	3	D	3
Oit30	G2	Oct_013	G2	31/05/14	1318	343	4	A	2
Oit30	G2	Oct_013	G2	31/05/14	1318	343	4	B	4
Oit30	G2	Oct_013	G2	31/05/14	1318	343	4	C	4
Oit30	G2	Oct_013	G2	31/05/14	1318	343	4	D	3
Oit15	G2	Oct_013	G2	29/05/14	902	285	1	A	3
Oit15	G2	Oct_013	G2	29/05/14	902	285	1	B	3
Oit15	G2	Oct_013	G2	29/05/14	902	285	1	C	3
Oit15	G2	Oct_013	G2	29/05/14	902	285	1	D	4
Oit15	G2	Oct_013	G2	29/05/14	903	269	2	A	2
Oit15	G2	Oct_013	G2	29/05/14	903	269	2	B	4
Oit15	G2	Oct_013	G2	29/05/14	903	269	2	C	3
Oit15	G2	Oct_013	G2	29/05/14	903	269	2	D	4
Oit15	G2	Oct_013	G2	29/05/14	904	233	3	A	2
Oit15	G2	Oct_013	G2	29/05/14	904	233	3	B	3
Oit15	G2	Oct_013	G2	29/05/14	904	233	3	C	3
Oit15	G2	Oct_013	G2	29/05/14	904	233	3	D	3
Oit26	G1	Oct_022	G1	27/05/14	788	110	1	A	0
Oit26	G1	Oct_022	G1	27/05/14	788	110	1	B	0
Oit26	G1	Oct_022	G1	27/05/14	788	110	1	C	0
Oit26	G1	Oct_022	G1	27/05/14	788	110	1	D	0
Oit26	G1	Oct_022	G1	27/05/14	789	139	2	A	0
Oit26	G1	Oct_022	G1	27/05/14	789	139	2	B	0
Oit26	G1	Oct_022	G1	27/05/14	789	139	2	C	0
Oit26	G1	Oct_022	G1	27/05/14	789	139	2	D	0
Oit27	G1	Oct_022	G1	31/05/14	1252	331	1	A	0
Oit27	G1	Oct_022	G1	31/05/14	1252	331	1	B	0
Oit27	G1	Oct_022	G1	31/05/14	1252	331	1	C	0
Oit27	G1	Oct_022	G1	31/05/14	1252	331	1	D	0
Oit27	G1	Oct_022	G1	31/05/14	1253	350	2	A	0
Oit27	G1	Oct_022	G1	31/05/14	1253	350	2	B	0
Oit27	G1	Oct_022	G1	31/05/14	1253	350	2	C	0
Oit27	G1	Oct_022	G1	31/05/14	1253	350	2	D	0
Oit27	G1	Oct_022	G1	31/05/14	1254	367	3	A	0
Oit27	G1	Oct_022	G1	31/05/14	1254	367	3	B	0
Oit27	G1	Oct_022	G1	31/05/14	1254	367	3	C	0
Oit27	G1	Oct_022	G1	31/05/14	1254	367	3	D	0
Oit26	G1	Oct_023	G1	27/05/14	721	90	1	A	2
Oit26	G1	Oct_023	G1	27/05/14	721	90	1	B	1
Oit26	G1	Oct_023	G1	27/05/14	721	90	1	C	1
Oit26	G1	Oct_023	G1	27/05/14	721	90	1	D	2
Oit26	G1	Oct_023	G1	27/05/14	722	1	2	A	3
Oit26	G1	Oct_023	G1	27/05/14	722	1	2	B	2
Oit26	G1	Oct_023	G1	27/05/14	722	1	2	C	2
Oit26	G1	Oct_023	G1	27/05/14	722	1	2	D	2
Oit26	G1	Oct_023	G1	27/05/14	723	152	3	A	No pollen
Oit26	G1	Oct_023	G1	27/05/14	723	152	3	B	No pollen

Pollen Recepient reference	SI Group of pollen Recipient	Pollen donor reference	SI Group of pollen donor	Pollination date	Photo reference number	photo random number	N° of flower	Repetition of flower read	Class of SI phenotype
Oit26	G1	Oct_023	G1	27/05/14	723	152	3	C	No pollen
Oit26	G1	Oct_023	G1	27/05/14	723	152	3	D	No pollen
Oit27	G1	Oct_023	G1	31/05/14	377	14	1	A	2
Oit27	G1	Oct_023	G1	31/05/14	377	14	1	B	2
Oit27	G1	Oct_023	G1	31/05/14	377	14	1	C	1
Oit27	G1	Oct_023	G1	31/05/14	377	14	1	D	2
Oit27	G1	Oct_023	G1	31/05/14	378	196	2	A	0
Oit27	G1	Oct_023	G1	31/05/14	378	196	2	B	0
Oit27	G1	Oct_023	G1	31/05/14	378	196	2	C	0
Oit27	G1	Oct_023	G1	31/05/14	378	196	2	D	0
Oit27	G1	Oct_023	G1	31/05/14	379	128	3	A	2
Oit27	G1	Oct_023	G1	31/05/14	379	128	3	B	2
Oit27	G1	Oct_023	G1	31/05/14	379	128	3	C	2
Oit27	G1	Oct_023	G1	31/05/14	379	128	3	D	2
Oit26	G1	Oct_024	G1	29/05/14	483	189	1	A	1
Oit26	G1	Oct_024	G1	29/05/14	483	189	1	B	1
Oit26	G1	Oct_024	G1	29/05/14	483	189	1	C	2
Oit26	G1	Oct_024	G1	29/05/14	483	189	1	D	1
Oit26	G1	Oct_024	G1	29/05/14	484	89	2	A	0
Oit26	G1	Oct_024	G1	29/05/14	484	89	2	B	0
Oit26	G1	Oct_024	G1	29/05/14	484	89	2	C	0
Oit26	G1	Oct_024	G1	29/05/14	484	89	2	D	0
Oit27	G1	Oct_024	G1	31/05/14	1257	338	1	A	1
Oit27	G1	Oct_024	G1	31/05/14	1257	338	1	B	1
Oit27	G1	Oct_024	G1	31/05/14	1257	338	1	C	0
Oit27	G1	Oct_024	G1	31/05/14	1257	338	1	D	0
Oit27	G1	Oct_024	G1	31/05/14	1258	330	2	A	2
Oit27	G1	Oct_024	G1	31/05/14	1258	330	2	B	2
Oit27	G1	Oct_024	G1	31/05/14	1258	330	2	C	2
Oit27	G1	Oct_024	G1	31/05/14	1258	330	2	D	2
Oit27	G1	Oct_024	G1	31/05/14	1259	311	3	A	1
Oit27	G1	Oct_024	G1	31/05/14	1259	311	3	B	1
Oit27	G1	Oct_024	G1	31/05/14	1259	311	3	C	0
Oit27	G1	Oct_024	G1	31/05/14	1259	311	3	D	1
Oit26	G1	Oct_025	G1	27/05/14	179	31	1	A	3
Oit26	G1	Oct_025	G1	27/05/14	179	31	1	B	3
Oit26	G1	Oct_025	G1	27/05/14	179	31	1	C	3
Oit26	G1	Oct_025	G1	27/05/14	179	31	1	D	4
Oit15	G1	Oct_025	G2	27/05/14	169	25	1	A	2
Oit15	G1	Oct_025	G2	27/05/14	169	25	1	B	2
Oit15	G1	Oct_025	G2	27/05/14	169	25	1	C	2
Oit15	G1	Oct_025	G2	27/05/14	169	25	1	D	2
Oit26	G1	Oct_027	G1	27/05/14	758	9	1	A	3
Oit26	G1	Oct_027	G1	27/05/14	758	9	1	B	2
Oit26	G1	Oct_027	G1	27/05/14	758	9	1	C	1
Oit26	G1	Oct_027	G1	27/05/14	758	9	1	D	1
Oit26	G1	Oct_027	G1	27/05/14	759	77	2	A	1
Oit26	G1	Oct_027	G1	27/05/14	759	77	2	B	1
Oit26	G1	Oct_027	G1	27/05/14	759	77	2	C	1
Oit26	G1	Oct_027	G1	27/05/14	759	77	2	D	1
Oit26	G1	Oct_027	G1	27/05/14	760	26	3	A	1
Oit26	G1	Oct_027	G1	27/05/14	760	26	3	B	1
Oit26	G1	Oct_027	G1	27/05/14	760	26	3	C	1
Oit26	G1	Oct_027	G1	27/05/14	760	26	3	D	0
Oit27	G1	Oct_027	G1	31/05/14	1219	307	1	A	1
Oit27	G1	Oct_027	G1	31/05/14	1219	307	1	B	2
Oit27	G1	Oct_027	G1	31/05/14	1219	307	1	C	1
Oit27	G1	Oct_027	G1	31/05/14	1219	307	1	D	2

Pollen Recepient reference	SI Group of pollen Recipient	Pollen donor reference	SI Group of pollen donor	Pollination date	Photo reference number	photo random number	N° of flower	Repetition of flower read	Class of SI phenotype
Oit27	G1	Oct_027	G1	31/05/14	1220	394	2	A	1
Oit27	G1	Oct_027	G1	31/05/14	1220	394	2	B	1
Oit27	G1	Oct_027	G1	31/05/14	1220	394	2	C	1
Oit27	G1	Oct_027	G1	31/05/14	1220	394	2	D	2
Oit27	G1	Oct_027	G1	31/05/14	1221	352	3	A	1
Oit27	G1	Oct_027	G1	31/05/14	1221	352	3	B	1
Oit27	G1	Oct_027	G1	31/05/14	1221	352	3	C	1
Oit27	G1	Oct_027	G1	31/05/14	1221	352	3	D	2
Oit26	G1	Oct_039	G1	27/05/14	748	134	1	A	0
Oit26	G1	Oct_039	G1	27/05/14	748	134	1	B	0
Oit26	G1	Oct_039	G1	27/05/14	748	134	1	C	0
Oit26	G1	Oct_039	G1	27/05/14	748	134	1	D	0
Oit26	G1	Oct_039	G1	27/05/14	749	184	2	A	0
Oit26	G1	Oct_039	G1	27/05/14	749	184	2	B	0
Oit26	G1	Oct_039	G1	27/05/14	749	184	2	C	0
Oit26	G1	Oct_039	G1	27/05/14	749	184	2	D	0
Oit27	G1	Oct_039	G1	31/05/14	1212	356	1	A	0
Oit27	G1	Oct_039	G1	31/05/14	1212	356	1	B	0
Oit27	G1	Oct_039	G1	31/05/14	1212	356	1	C	0
Oit27	G1	Oct_039	G1	31/05/14	1212	356	1	D	0
Oit27	G1	Oct_039	G1	31/05/14	1213	345	2	A	0
Oit27	G1	Oct_039	G1	31/05/14	1213	345	2	B	0
Oit27	G1	Oct_039	G1	31/05/14	1213	345	2	C	0
Oit27	G1	Oct_039	G1	31/05/14	1213	345	2	D	0
Oit27	G1	Oct_039	G1	31/05/14	1214	365	3	A	0
Oit27	G1	Oct_039	G1	31/05/14	1214	365	3	B	0
Oit27	G1	Oct_039	G1	31/05/14	1214	365	3	C	0
Oit27	G1	Oct_039	G1	31/05/14	1214	365	3	D	0
Oit27	G1	Oct_039	G1	31/05/14	1215	309	4	A	0
Oit27	G1	Oct_039	G1	31/05/14	1215	309	4	B	0
Oit27	G1	Oct_039	G1	31/05/14	1215	309	4	C	0
Oit27	G1	Oct_039	G1	31/05/14	1215	309	4	D	0
Oit26	G1	Oct_043	G1	27/05/14	157	47	1	A	0
Oit26	G1	Oct_043	G1	27/05/14	157	47	1	B	0
Oit26	G1	Oct_043	G1	27/05/14	157	47	1	C	0
Oit26	G1	Oct_043	G1	27/05/14	157	47	1	D	0
Oit27	G1	Oct_043	G1	31/05/14	1148	379	1	A	0
Oit27	G1	Oct_043	G1	31/05/14	1148	379	1	B	0
Oit27	G1	Oct_043	G1	31/05/14	1148	379	1	C	0
Oit27	G1	Oct_043	G1	31/05/14	1148	379	1	D	0
Oit27	G1	Oct_043	G1	31/05/14	1149	325	2	A	0
Oit27	G1	Oct_043	G1	31/05/14	1149	325	2	B	0
Oit27	G1	Oct_043	G1	31/05/14	1149	325	2	C	0
Oit27	G1	Oct_043	G1	31/05/14	1149	325	2	D	0
Oit30	G2	Oct_045	G2	31/05/14	1202	360	1	A	0
Oit30	G2	Oct_045	G2	31/05/14	1202	360	1	B	0
Oit30	G2	Oct_045	G2	31/05/14	1202	360	1	C	0
Oit30	G2	Oct_045	G2	31/05/14	1202	360	1	D	0
Oit30	G2	Oct_045	G2	31/05/14	1203	368	2	A	0
Oit30	G2	Oct_045	G2	31/05/14	1203	368	2	B	0
Oit30	G2	Oct_045	G2	31/05/14	1203	368	2	C	0
Oit30	G2	Oct_045	G2	31/05/14	1203	368	2	D	0
Oit30	G2	Oct_045	G2	31/05/14	1204	369	3	A	0
Oit30	G2	Oct_045	G2	31/05/14	1204	369	3	B	0
Oit30	G2	Oct_045	G2	31/05/14	1204	369	3	C	0
Oit30	G2	Oct_045	G2	31/05/14	1204	369	3	D	0
Oit15	G2	Oct_045	G2	27/05/14	742	86	1	A	0
Oit15	G2	Oct_045	G2	27/05/14	742	86	1	B	0

Pollen Recepient reference	SI Group of pollen Recipient	Pollen donor reference	SI Group of pollen donor	Pollination date	Photo reference number	photo random number	N° of flower	Repetition of flower read	Class of SI phenotype
Oit15	G2	Oct_045	G2	27/05/14	742	86	1	C	0
Oit15	G2	Oct_045	G2	27/05/14	742	86	1	D	0
Oit15	G2	Oct_045	G2	27/05/14	743	11	2	A	1
Oit15	G2	Oct_045	G2	27/05/14	743	11	2	B	0
Oit15	G2	Oct_045	G2	27/05/14	743	11	2	C	0
Oit15	G2	Oct_045	G2	27/05/14	743	11	2	D	0
Oit26	G1	Oct_052	G1	27/05/14	733	94	1	A	0
Oit26	G1	Oct_052	G1	27/05/14	733	94	1	B	0
Oit26	G1	Oct_052	G1	27/05/14	733	94	1	C	0
Oit26	G1	Oct_052	G1	27/05/14	733	94	1	D	0
Oit26	G1	Oct_052	G1	27/05/14	734	36	2	A	1
Oit26	G1	Oct_052	G1	27/05/14	734	36	2	B	1
Oit26	G1	Oct_052	G1	27/05/14	734	36	2	C	0
Oit26	G1	Oct_052	G1	27/05/14	734	36	2	D	0
Oit26	G1	Oct_052	G1	27/05/14	735	24	3	A	1
Oit26	G1	Oct_052	G1	27/05/14	735	24	3	B	1
Oit26	G1	Oct_052	G1	27/05/14	735	24	3	C	0
Oit26	G1	Oct_052	G1	27/05/14	735	24	3	D	1
Oit27	G1	Oct_052	G1	31/05/14	1194	385	2	A	0
Oit27	G1	Oct_052	G1	31/05/14	1194	385	2	B	0
Oit27	G1	Oct_052	G1	31/05/14	1194	385	2	C	0
Oit27	G1	Oct_052	G1	31/05/14	1194	385	2	D	0
Oit27	G1	Oct_052	G1	31/05/14	1195	374	3	A	0
Oit27	G1	Oct_052	G1	31/05/14	1195	374	3	B	0
Oit27	G1	Oct_052	G1	31/05/14	1195	374	3	C	0
Oit27	G1	Oct_052	G1	31/05/14	1195	374	3	D	0
Oit30	G2	Oct_063	G2	31/05/14	1164	354	2	A	1
Oit30	G2	Oct_063	G2	31/05/14	1164	354	2	B	1
Oit30	G2	Oct_063	G2	31/05/14	1164	354	2	C	1
Oit30	G2	Oct_063	G2	31/05/14	1164	354	2	D	1
Oit30	G2	Oct_063	G2	31/05/14	1166	321	3	A	1
Oit30	G2	Oct_063	G2	31/05/14	1166	321	3	B	1
Oit30	G2	Oct_063	G2	31/05/14	1166	321	3	C	1
Oit30	G2	Oct_063	G2	31/05/14	1166	321	3	D	2
Oit15	G2	Oct_063	G2	27/05/14	201	23	1	A	2
Oit15	G2	Oct_063	G2	27/05/14	201	23	1	B	1
Oit15	G2	Oct_063	G2	27/05/14	201	23	1	C	0
Oit15	G2	Oct_063	G2	27/05/14	201	23	1	D	1
Oit15	G2	Oct_063	G2	27/05/14	202	34	2	A	2
Oit15	G2	Oct_063	G2	27/05/14	202	34	2	B	2
Oit15	G2	Oct_063	G2	27/05/14	202	34	2	C	2
Oit15	G2	Oct_063	G2	27/05/14	202	34	2	D	2
Oit15	G2	Oct_063	G2	27/05/14	203	191	3	A	1
Oit15	G2	Oct_063	G2	27/05/14	203	191	3	B	1
Oit15	G2	Oct_063	G2	27/05/14	203	191	3	C	1
Oit15	G2	Oct_063	G2	27/05/14	203	191	3	D	2
Oit26	G1	Oct_065	G1	27/05/14	768	27	1	A	0
Oit26	G1	Oct_065	G1	27/05/14	768	27	1	B	0
Oit26	G1	Oct_065	G1	27/05/14	768	27	1	C	0
Oit26	G1	Oct_065	G1	27/05/14	768	27	1	D	0
Oit26	G1	Oct_065	G1	27/05/14	769	3	2	A	2
Oit26	G1	Oct_065	G1	27/05/14	769	3	2	B	0
Oit26	G1	Oct_065	G1	27/05/14	769	3	2	C	0
Oit26	G1	Oct_065	G1	27/05/14	769	3	2	D	0
Oit26	G1	Oct_065	G1	27/05/14	770	113	3	A	0
Oit26	G1	Oct_065	G1	27/05/14	770	113	3	B	0
Oit26	G1	Oct_065	G1	27/05/14	770	113	3	C	0
Oit26	G1	Oct_065	G1	27/05/14	770	113	3	D	0

Pollen Recepient reference	SI Group of pollen Recipient	Pollen donor reference	SI Group of pollen donor	Pollination date	Photo reference number	photo random number	N° of flower	Repetition of flower read	Class of SI phenotype
Oit27	G1	Oct_065	G1	31/05/14	1229	344	1	A	1
Oit27	G1	Oct_065	G1	31/05/14	1229	344	1	B	1
Oit27	G1	Oct_065	G1	31/05/14	1229	344	1	C	1
Oit27	G1	Oct_065	G1	31/05/14	1229	344	1	D	1
Oit27	G1	Oct_065	G1	31/05/14	1230	348	2	A	1
Oit27	G1	Oct_065	G1	31/05/14	1230	348	2	B	1
Oit27	G1	Oct_065	G1	31/05/14	1230	348	2	C	1
Oit27	G1	Oct_065	G1	31/05/14	1230	348	2	D	0
Oit27	G1	Oct_065	G1	31/05/14	1231	380	3	A	1
Oit27	G1	Oct_065	G1	31/05/14	1231	380	3	B	1
Oit27	G1	Oct_065	G1	31/05/14	1231	380	3	C	1
Oit27	G1	Oct_065	G1	31/05/14	1231	380	3	D	2
Oit30	G2	Oct_084	G2	31/05/14	1068	245	1	A	1
Oit30	G2	Oct_084	G2	31/05/14	1068	245	1	B	1
Oit30	G2	Oct_084	G2	31/05/14	1068	245	1	C	1
Oit30	G2	Oct_084	G2	31/05/14	1068	245	1	D	1
Oit30	G2	Oct_084	G2	31/05/14	1069	237	2	A	1
Oit30	G2	Oct_084	G2	31/05/14	1069	237	2	B	2
Oit30	G2	Oct_084	G2	31/05/14	1069	237	2	C	1
Oit30	G2	Oct_084	G2	31/05/14	1069	237	2	D	1
Oit30	G2	Oct_084	G2	31/05/14	1070	232	3	A	1
Oit30	G2	Oct_084	G2	31/05/14	1070	232	3	B	1
Oit30	G2	Oct_084	G2	31/05/14	1070	232	3	C	1
Oit30	G2	Oct_084	G2	31/05/14	1070	232	3	D	2
Oit15	G2	Oct_084	G2	27/05/14	694	73	1	A	4
Oit15	G2	Oct_084	G2	27/05/14	694	73	1	B	4
Oit15	G2	Oct_084	G2	27/05/14	694	73	1	C	4
Oit15	G2	Oct_084	G2	27/05/14	694	73	1	D	3
Oit15	G2	Oct_084	G2	27/05/14	695	71	2	A	3
Oit15	G2	Oct_084	G2	27/05/14	695	71	2	B	3
Oit15	G2	Oct_084	G2	27/05/14	695	71	2	C	4
Oit15	G2	Oct_084	G2	27/05/14	695	71	2	D	3
Oit15	G2	Oct_084	G2	27/05/14	696	105	3	A	2
Oit15	G2	Oct_084	G2	27/05/14	696	105	3	B	3
Oit15	G2	Oct_084	G2	27/05/14	696	105	3	C	3
Oit15	G2	Oct_084	G2	27/05/14	696	105	3	D	3
Oit26	G1	Oct_088	G1	29/05/14	979	289	1	A	0
Oit26	G1	Oct_088	G1	29/05/14	979	289	1	B	0
Oit26	G1	Oct_088	G1	29/05/14	979	289	1	C	0
Oit26	G1	Oct_088	G1	29/05/14	979	289	1	D	0
Oit26	G1	Oct_088	G1	29/05/14	980	266	2	A	0
Oit26	G1	Oct_088	G1	29/05/14	980	266	2	B	0
Oit26	G1	Oct_088	G1	29/05/14	980	266	2	C	0
Oit26	G1	Oct_088	G1	29/05/14	980	266	2	D	0
Oit26	G1	Oct_088	G1	29/05/14	981	241	3	A	0
Oit26	G1	Oct_088	G1	29/05/14	981	241	3	B	0
Oit26	G1	Oct_088	G1	29/05/14	981	241	3	C	0
Oit26	G1	Oct_088	G1	29/05/14	981	241	3	D	0
Oit27	G1	Oct_088	G1	31/05/14	365	19	1	A	0
Oit27	G1	Oct_088	G1	31/05/14	365	19	1	B	0
Oit27	G1	Oct_088	G1	31/05/14	365	19	1	C	0
Oit27	G1	Oct_088	G1	31/05/14	365	19	1	D	0
Oit27	G1	Oct_088	G1	31/05/14	366	190	2	A	0
Oit27	G1	Oct_088	G1	31/05/14	366	190	2	B	0
Oit27	G1	Oct_088	G1	31/05/14	366	190	2	C	0
Oit27	G1	Oct_088	G1	31/05/14	366	190	2	D	0
Oit27	G1	Oct_088	G1	31/05/14	367	88	3	A	0
Oit27	G1	Oct_088	G1	31/05/14	367	88	3	B	0

Pollen Recepient reference	SI Group of pollen Recipient	Pollen donor reference	SI Group of pollen donor	Pollination date	Photo reference number	photo random number	N° of flower	Repetition of flower read	Class of SI phenotype
Oit27	G1	Oct_088	G1	31/05/14	367	88	3	C	0
Oit27	G1	Oct_088	G1	31/05/14	367	88	3	D	0
Oit30	G2	Oct_091	G2	31/05/14	1266	332	1	A	1
Oit30	G2	Oct_091	G2	31/05/14	1266	332	1	B	1
Oit30	G2	Oct_091	G2	31/05/14	1266	332	1	C	1
Oit30	G2	Oct_091	G2	31/05/14	1266	332	1	D	1
Oit30	G2	Oct_091	G2	31/05/14	1267	391	2	A	0
Oit30	G2	Oct_091	G2	31/05/14	1267	391	2	B	0
Oit30	G2	Oct_091	G2	31/05/14	1267	391	2	C	0
Oit30	G2	Oct_091	G2	31/05/14	1267	391	2	D	0
Oit30	G2	Oct_091	G2	31/05/14	1268	347	3	A	0
Oit30	G2	Oct_091	G2	31/05/14	1268	347	3	B	0
Oit30	G2	Oct_091	G2	31/05/14	1268	347	3	C	0
Oit30	G2	Oct_091	G2	31/05/14	1268	347	3	D	0
Oit15	G2	Oct_091	G2	29/05/14	854	51	1	A	1
Oit15	G2	Oct_091	G2	29/05/14	854	51	1	B	1
Oit15	G2	Oct_091	G2	29/05/14	854	51	1	C	0
Oit15	G2	Oct_091	G2	29/05/14	854	51	1	D	0
Oit30	G2	Oct_101	G2	31/05/14	1116	271	1	A	1
Oit30	G2	Oct_101	G2	31/05/14	1116	271	1	B	0
Oit30	G2	Oct_101	G2	31/05/14	1116	271	1	C	1
Oit30	G2	Oct_101	G2	31/05/14	1116	271	1	D	0
Oit30	G2	Oct_101	G2	31/05/14	1117	204	2	A	1
Oit30	G2	Oct_101	G2	31/05/14	1117	204	2	B	1
Oit30	G2	Oct_101	G2	31/05/14	1117	204	2	C	1
Oit30	G2	Oct_101	G2	31/05/14	1117	204	2	D	1
Oit30	G2	Oct_101	G2	31/05/14	1118	299	3	A	0
Oit30	G2	Oct_101	G2	31/05/14	1118	299	3	B	0
Oit30	G2	Oct_101	G2	31/05/14	1118	299	3	C	0
Oit30	G2	Oct_101	G2	31/05/14	1118	299	3	D	0
Oit15	G2	Oct_101	G2	27/05/14	636	193	1	A	2
Oit15	G2	Oct_101	G2	27/05/14	636	193	1	B	3
Oit15	G2	Oct_101	G2	27/05/14	636	193	1	C	3
Oit15	G2	Oct_101	G2	27/05/14	636	193	1	D	3
Oit15	G2	Oct_101	G2	27/05/14	637	95	2	A	1
Oit15	G2	Oct_101	G2	27/05/14	637	95	2	B	1
Oit15	G2	Oct_101	G2	27/05/14	637	95	2	C	1
Oit15	G2	Oct_101	G2	27/05/14	637	95	2	D	1
Oit15	G2	Oct_101	G2	27/05/14	638	185	3	A	1
Oit15	G2	Oct_101	G2	27/05/14	638	185	3	B	2
Oit15	G2	Oct_101	G2	27/05/14	638	185	3	C	2
Oit15	G2	Oct_101	G2	27/05/14	638	185	3	D	2
Oit26	G1	Oct_103	G1	27/05/14	738	161	1	A	1
Oit26	G1	Oct_103	G1	27/05/14	738	161	1	B	1
Oit26	G1	Oct_103	G1	27/05/14	738	161	1	C	1
Oit26	G1	Oct_103	G1	27/05/14	738	161	1	D	1
Oit26	G1	Oct_103	G1	27/05/14	739	183	2	A	0
Oit26	G1	Oct_103	G1	27/05/14	739	183	2	B	0
Oit26	G1	Oct_103	G1	27/05/14	739	183	2	C	0
Oit26	G1	Oct_103	G1	27/05/14	739	183	2	D	0
Oit26	G1	Oct_103	G1	27/05/14	740	129	3	A	1
Oit26	G1	Oct_103	G1	27/05/14	740	129	3	B	1
Oit26	G1	Oct_103	G1	27/05/14	740	129	3	C	0
Oit26	G1	Oct_103	G1	27/05/14	740	129	3	D	1
Oit27	G1	Oct_103	G1	31/05/14	1199	310	1	A	1
Oit27	G1	Oct_103	G1	31/05/14	1199	310	1	B	1
Oit27	G1	Oct_103	G1	31/05/14	1199	310	1	C	0
Oit27	G1	Oct_103	G1	31/05/14	1199	310	1	D	1

Pollen Recepient reference	SI Group of pollen Recipient	Pollen donor reference	SI Group of pollen donor	Pollination date	Photo reference number	photo random number	N° of flower	Repetition of flower read	Class of SI phenotype
Oit27	G1	Oct_103	G1	31/05/14	1200	376	2	A	1
Oit27	G1	Oct_103	G1	31/05/14	1200	376	2	B	1
Oit27	G1	Oct_103	G1	31/05/14	1200	376	2	C	0
Oit27	G1	Oct_103	G1	31/05/14	1200	376	2	D	1
Oit27	G1	Oct_103	G1	31/05/14	1201	317	3	A	2
Oit27	G1	Oct_103	G1	31/05/14	1201	317	3	B	2
Oit27	G1	Oct_103	G1	31/05/14	1201	317	3	C	2
Oit27	G1	Oct_103	G1	31/05/14	1201	317	3	D	2
Oit26	G1	Oct_120	G1	27/05/14	779	188	1	A	0
Oit26	G1	Oct_120	G1	27/05/14	779	188	1	B	0
Oit26	G1	Oct_120	G1	27/05/14	779	188	1	C	0
Oit26	G1	Oct_120	G1	27/05/14	779	188	1	D	0
Oit26	G1	Oct_120	G1	27/05/14	780	18	2	A	0
Oit26	G1	Oct_120	G1	27/05/14	780	18	2	B	0
Oit26	G1	Oct_120	G1	27/05/14	780	18	2	C	0
Oit26	G1	Oct_120	G1	27/05/14	780	18	2	D	0
Oit26	G1	Oct_120	G1	27/05/14	781	178	3	A	0
Oit26	G1	Oct_120	G1	27/05/14	781	178	3	B	0
Oit26	G1	Oct_120	G1	27/05/14	781	178	3	C	0
Oit26	G1	Oct_120	G1	27/05/14	781	178	3	D	0
Oit27	G1	Oct_120	G1	31/05/14	1240	315	1	A	0
Oit27	G1	Oct_120	G1	31/05/14	1240	315	1	B	0
Oit27	G1	Oct_120	G1	31/05/14	1240	315	1	C	0
Oit27	G1	Oct_120	G1	31/05/14	1240	315	1	D	0
Oit27	G1	Oct_120	G1	31/05/14	1241	382	2	A	0
Oit27	G1	Oct_120	G1	31/05/14	1241	382	2	B	0
Oit27	G1	Oct_120	G1	31/05/14	1241	382	2	C	0
Oit27	G1	Oct_120	G1	31/05/14	1241	382	2	D	0
Oit26	G1	Oct_124	G1	29/05/14	900	225	1	A	1
Oit26	G1	Oct_124	G1	29/05/14	900	225	1	B	1
Oit26	G1	Oct_124	G1	29/05/14	900	225	1	C	1
Oit26	G1	Oct_124	G1	29/05/14	900	225	1	D	1
Oit26	G1	Oct_124	G1	29/05/14	901	287	2	A	0
Oit26	G1	Oct_124	G1	29/05/14	901	287	2	B	1
Oit26	G1	Oct_124	G1	29/05/14	901	287	2	C	0
Oit26	G1	Oct_124	G1	29/05/14	901	287	2	D	0
Oit27	G1	Oct_124	G1	31/05/14	1312	364	1	A	0
Oit27	G1	Oct_124	G1	31/05/14	1312	364	1	B	0
Oit27	G1	Oct_124	G1	31/05/14	1312	364	1	C	0
Oit27	G1	Oct_124	G1	31/05/14	1312	364	1	D	0
Oit27	G1	Oct_124	G1	31/05/14	1313	387	2	A	1
Oit27	G1	Oct_124	G1	31/05/14	1313	387	2	B	1
Oit27	G1	Oct_124	G1	31/05/14	1313	387	2	C	1
Oit27	G1	Oct_124	G1	31/05/14	1313	387	2	D	2
Oit27	G1	Oct_124	G1	31/05/14	1314	340	3	A	1
Oit27	G1	Oct_124	G1	31/05/14	1314	340	3	B	0
Oit27	G1	Oct_124	G1	31/05/14	1314	340	3	C	1
Oit27	G1	Oct_124	G1	31/05/14	1314	340	3	D	0
Oit30	G2	Oct_145	G2	31/05/14	1150	303	1	A	1
Oit30	G2	Oct_145	G2	31/05/14	1150	303	1	B	2
Oit30	G2	Oct_145	G2	31/05/14	1150	303	1	C	1
Oit30	G2	Oct_145	G2	31/05/14	1150	303	1	D	2
Oit30	G2	Oct_145	G2	31/05/14	1152	312	3	A	1
Oit30	G2	Oct_145	G2	31/05/14	1152	312	3	B	1
Oit30	G2	Oct_145	G2	31/05/14	1152	312	3	C	1
Oit30	G2	Oct_145	G2	31/05/14	1152	312	3	D	2
Oit15	G2	Oct_145	G2	27/05/14	158	58	1	A	0
Oit15	G2	Oct_145	G2	27/05/14	158	58	1	B	0

Pollen Recepient reference	SI Group of pollen Recipient	Pollen donor reference	SI Group of pollen donor	Pollination date	Photo reference number	photo random number	N° of flower	Repetition of flower read	Class of SI phenotype
Oit15	G2	Oct_145	G2	27/05/14	158	58	1	C	0
Oit15	G2	Oct_145	G2	27/05/14	158	58	1	D	0
Oit15	G2	Oct_145	G2	27/05/14	159	104	2	A	1
Oit15	G2	Oct_145	G2	27/05/14	159	104	2	B	1
Oit15	G2	Oct_145	G2	27/05/14	159	104	2	C	2
Oit15	G2	Oct_145	G2	27/05/14	159	104	2	D	1
Oit26	G1	Oct_147	G1	27/05/14	691	84	1	A	1
Oit26	G1	Oct_147	G1	27/05/14	691	84	1	B	1
Oit26	G1	Oct_147	G1	27/05/14	691	84	1	C	1
Oit26	G1	Oct_147	G1	27/05/14	691	84	1	D	0
Oit26	G1	Oct_147	G1	27/05/14	692	28	2	A	0
Oit26	G1	Oct_147	G1	27/05/14	692	28	2	B	0
Oit26	G1	Oct_147	G1	27/05/14	692	28	2	C	0
Oit26	G1	Oct_147	G1	27/05/14	692	28	2	D	0
Oit26	G1	Oct_147	G1	27/05/14	693	147	3	A	0
Oit26	G1	Oct_147	G1	27/05/14	693	147	3	B	0
Oit26	G1	Oct_147	G1	27/05/14	693	147	3	C	0
Oit26	G1	Oct_147	G1	27/05/14	693	147	3	D	0
Oit27	G1	Oct_147	G1	31/05/14	1065	279	1	A	0
Oit27	G1	Oct_147	G1	31/05/14	1065	279	1	B	0
Oit27	G1	Oct_147	G1	31/05/14	1065	279	1	C	0
Oit27	G1	Oct_147	G1	31/05/14	1065	279	1	D	0
Oit27	G1	Oct_147	G1	31/05/14	1066	222	2	A	0
Oit27	G1	Oct_147	G1	31/05/14	1066	222	2	B	0
Oit27	G1	Oct_147	G1	31/05/14	1066	222	2	C	0
Oit27	G1	Oct_147	G1	31/05/14	1066	222	2	D	0
Oit27	G1	Oct_147	G1	31/05/14	1067	215	3	A	0
Oit27	G1	Oct_147	G1	31/05/14	1067	215	3	B	0
Oit27	G1	Oct_147	G1	31/05/14	1067	215	3	C	0
Oit27	G1	Oct_147	G1	31/05/14	1067	215	3	D	0
Oit26	G1	Oct_149	G1	27/05/14	656	16	1	A	2
Oit26	G1	Oct_149	G1	27/05/14	656	16	1	B	1
Oit26	G1	Oct_149	G1	27/05/14	656	16	1	C	1
Oit26	G1	Oct_149	G1	27/05/14	656	16	1	D	2
Oit26	G1	Oct_149	G1	27/05/14	657	53	2	A	2
Oit26	G1	Oct_149	G1	27/05/14	657	53	2	B	2
Oit26	G1	Oct_149	G1	27/05/14	657	53	2	C	2
Oit26	G1	Oct_149	G1	27/05/14	657	53	2	D	2
Oit26	G1	Oct_149	G1	27/05/14	658	13	3	A	1
Oit26	G1	Oct_149	G1	27/05/14	658	13	3	B	1
Oit26	G1	Oct_149	G1	27/05/14	658	13	3	C	0
Oit26	G1	Oct_149	G1	27/05/14	658	13	3	D	0
Oit27	G1	Oct_149	G1	31/05/14	1100	228	1	A	1
Oit27	G1	Oct_149	G1	31/05/14	1100	228	1	B	1
Oit27	G1	Oct_149	G1	31/05/14	1100	228	1	C	1
Oit27	G1	Oct_149	G1	31/05/14	1100	228	1	D	1
Oit27	G1	Oct_149	G1	31/05/14	1101	262	2	A	1
Oit27	G1	Oct_149	G1	31/05/14	1101	262	2	B	2
Oit27	G1	Oct_149	G1	31/05/14	1101	262	2	C	1
Oit27	G1	Oct_149	G1	31/05/14	1101	262	2	D	2
Oit27	G1	Oct_149	G1	31/05/14	1102	230	3	A	2
Oit27	G1	Oct_149	G1	31/05/14	1102	230	3	B	1
Oit27	G1	Oct_149	G1	31/05/14	1102	230	3	C	2
Oit27	G1	Oct_149	G1	31/05/14	1102	230	3	D	2
Oit26	G1	Oct_150	G1	27/05/14	187	69	1	A	0
Oit26	G1	Oct_150	G1	27/05/14	187	69	1	B	0
Oit26	G1	Oct_150	G1	27/05/14	187	69	1	C	0
Oit26	G1	Oct_150	G1	27/05/14	187	69	1	D	0

Pollen Recepient reference	SI Group of pollen Recipient	Pollen donor reference	SI Group of pollen donor	Pollination date	Photo reference number	photo random number	N° of flower	Repetition of flower read	Class of SI phenotype
Oit26	G1	Oct_150	G1	27/05/14	188	112	2	A	1
Oit26	G1	Oct_150	G1	27/05/14	188	112	2	B	1
Oit26	G1	Oct_150	G1	27/05/14	188	112	2	C	1
Oit26	G1	Oct_150	G1	27/05/14	188	112	2	D	1
Oit26	G1	Oct_150	G1	27/05/14	189	57	3	A	1
Oit26	G1	Oct_150	G1	27/05/14	189	57	3	B	1
Oit26	G1	Oct_150	G1	27/05/14	189	57	3	C	0
Oit26	G1	Oct_150	G1	27/05/14	189	57	3	D	0
Oit27	G1	Oct_150	G1	31/05/14	1157	386	1	A	1
Oit27	G1	Oct_150	G1	31/05/14	1157	386	1	B	1
Oit27	G1	Oct_150	G1	31/05/14	1157	386	1	C	1
Oit27	G1	Oct_150	G1	31/05/14	1157	386	1	D	1
Oit27	G1	Oct_150	G1	31/05/14	1158	339	2	A	1
Oit27	G1	Oct_150	G1	31/05/14	1158	339	2	B	0
Oit27	G1	Oct_150	G1	31/05/14	1158	339	2	C	0
Oit27	G1	Oct_150	G1	31/05/14	1158	339	2	D	0
Oit27	G1	Oct_150	G1	31/05/14	1159	346	3	A	1
Oit27	G1	Oct_150	G1	31/05/14	1159	346	3	B	1
Oit27	G1	Oct_150	G1	31/05/14	1159	346	3	C	1
Oit27	G1	Oct_150	G1	31/05/14	1159	346	3	D	1
Oit30	G2	Oct_189	G2	31/05/14	413	85	1	A	2
Oit30	G2	Oct_189	G2	31/05/14	413	85	1	B	2
Oit30	G2	Oct_189	G2	31/05/14	413	85	1	C	2
Oit30	G2	Oct_189	G2	31/05/14	413	85	1	D	2
Oit30	G2	Oct_189	G2	31/05/14	414	118	2	A	1
Oit30	G2	Oct_189	G2	31/05/14	414	118	2	B	1
Oit30	G2	Oct_189	G2	31/05/14	414	118	2	C	2
Oit30	G2	Oct_189	G2	31/05/14	414	118	2	D	2
Oit30	G2	Oct_189	G2	31/05/14	415	55	3	A	1
Oit30	G2	Oct_189	G2	31/05/14	415	55	3	B	1
Oit30	G2	Oct_189	G2	31/05/14	415	55	3	C	2
Oit30	G2	Oct_189	G2	31/05/14	415	55	3	D	1
Oit15	G2	Oct_189	G2	27/05/14	713	153	2	A	3
Oit15	G2	Oct_189	G2	27/05/14	713	153	2	B	4
Oit15	G2	Oct_189	G2	27/05/14	713	153	2	C	4
Oit15	G2	Oct_189	G2	27/05/14	713	153	2	D	4
Oit15	G2	Oct_189	G2	27/05/14	714	197	3	A	2
Oit15	G2	Oct_189	G2	27/05/14	714	197	3	B	1
Oit15	G2	Oct_189	G2	27/05/14	714	197	3	C	2
Oit15	G2	Oct_189	G2	27/05/14	714	197	3	D	2
Oit30	G2	Oct_207	G2	31/05/14	344	4	1	A	1
Oit30	G2	Oct_207	G2	31/05/14	344	4	1	B	1
Oit30	G2	Oct_207	G2	31/05/14	344	4	1	C	1
Oit30	G2	Oct_207	G2	31/05/14	344	4	1	D	1
Oit30	G2	Oct_207	G2	31/05/14	345	49	2	A	2
Oit30	G2	Oct_207	G2	31/05/14	345	49	2	B	2
Oit30	G2	Oct_207	G2	31/05/14	345	49	2	C	2
Oit30	G2	Oct_207	G2	31/05/14	345	49	2	D	3
Oit30	G2	Oct_207	G2	31/05/14	346	138	3	A	1
Oit30	G2	Oct_207	G2	31/05/14	346	138	3	B	2
Oit30	G2	Oct_207	G2	31/05/14	346	138	3	C	2
Oit30	G2	Oct_207	G2	31/05/14	346	138	3	D	2
Oit15	G2	Oct_207	G2	29/05/14	959	253	1	A	2
Oit15	G2	Oct_207	G2	29/05/14	959	253	1	B	2
Oit15	G2	Oct_207	G2	29/05/14	959	253	1	C	2
Oit15	G2	Oct_207	G2	29/05/14	959	253	1	D	2
Oit15	G2	Oct_207	G2	29/05/14	960	238	2	A	2
Oit15	G2	Oct_207	G2	29/05/14	960	238	2	B	2

Pollen Recepient reference	SI Group of pollen Recipient	Pollen donor reference	SI Group of pollen donor	Pollination date	Photo reference number	photo random number	N° of flower	Repetition of flower read	Class of SI phenotype
Oit15	G2	Oct_207	G2	29/05/14	960	238	2	C	2
Oit15	G2	Oct_207	G2	29/05/14	960	238	2	D	2
Oit15	G2	Oct_207	G2	29/05/14	961	242	3	A	3
Oit15	G2	Oct_207	G2	29/05/14	961	242	3	B	3
Oit15	G2	Oct_207	G2	29/05/14	961	242	3	C	3
Oit15	G2	Oct_207	G2	29/05/14	961	242	3	D	3
Oit30	G2	Oct_215	G2	31/05/14	1299	383	1	A	1
Oit30	G2	Oct_215	G2	31/05/14	1299	383	1	B	1
Oit30	G2	Oct_215	G2	31/05/14	1299	383	1	C	1
Oit30	G2	Oct_215	G2	31/05/14	1299	383	1	D	2
Oit30	G2	Oct_215	G2	31/05/14	1300	349	2	A	1
Oit30	G2	Oct_215	G2	31/05/14	1300	349	2	B	1
Oit30	G2	Oct_215	G2	31/05/14	1300	349	2	C	0
Oit30	G2	Oct_215	G2	31/05/14	1300	349	2	D	0
Oit30	G2	Oct_215	G2	31/05/14	1301	334	3	A	2
Oit30	G2	Oct_215	G2	31/05/14	1301	334	3	B	2
Oit30	G2	Oct_215	G2	31/05/14	1301	334	3	C	3
Oit30	G2	Oct_215	G2	31/05/14	1301	334	3	D	2
Oit15	G2	Oct_215	G2	29/05/14	885	223	1	A	2
Oit15	G2	Oct_215	G2	29/05/14	885	223	1	B	3
Oit15	G2	Oct_215	G2	29/05/14	885	223	1	C	2
Oit15	G2	Oct_215	G2	29/05/14	885	223	1	D	2
Oit15	G2	Oct_215	G2	29/05/14	886	258	2	A	2
Oit15	G2	Oct_215	G2	29/05/14	886	258	2	B	2
Oit15	G2	Oct_215	G2	29/05/14	886	258	2	C	2
Oit15	G2	Oct_215	G2	29/05/14	886	258	2	D	2
Oit15	G2	Oct_215	G2	29/05/14	887	234	3	A	2
Oit15	G2	Oct_215	G2	29/05/14	887	234	3	B	3
Oit15	G2	Oct_215	G2	29/05/14	887	234	3	C	3
Oit15	G2	Oct_215	G2	29/05/14	887	234	3	D	3
Oit30	G2	Oct_216	G2	31/05/14	1260	301	1	A	2
Oit30	G2	Oct_216	G2	31/05/14	1260	301	1	B	2
Oit30	G2	Oct_216	G2	31/05/14	1260	301	1	C	3
Oit30	G2	Oct_216	G2	31/05/14	1260	301	1	D	3
Oit30	G2	Oct_216	G2	31/05/14	1261	393	2	A	1
Oit30	G2	Oct_216	G2	31/05/14	1261	393	2	B	1
Oit30	G2	Oct_216	G2	31/05/14	1261	393	2	C	1
Oit30	G2	Oct_216	G2	31/05/14	1261	393	2	D	2
Oit30	G2	Oct_216	G2	31/05/14	1262	359	3	A	1
Oit30	G2	Oct_216	G2	31/05/14	1262	359	3	B	0
Oit30	G2	Oct_216	G2	31/05/14	1262	359	3	C	3
Oit30	G2	Oct_216	G2	31/05/14	1262	359	3	D	3
Oit15	G2	Oct_216	G2	29/05/14	848	96	1	A	3
Oit15	G2	Oct_216	G2	29/05/14	848	96	1	B	4
Oit15	G2	Oct_216	G2	29/05/14	848	96	1	C	4
Oit15	G2	Oct_216	G2	29/05/14	848	96	1	D	4
Oit15	G2	Oct_216	G2	29/05/14	849	103	2	A	2
Oit15	G2	Oct_216	G2	29/05/14	849	103	2	B	2
Oit15	G2	Oct_216	G2	29/05/14	849	103	2	C	3
Oit15	G2	Oct_216	G2	29/05/14	849	103	2	D	2
Oit15	G2	Oct_216	G2	29/05/14	850	179	3	A	2
Oit15	G2	Oct_216	G2	29/05/14	850	179	3	B	3
Oit15	G2	Oct_216	G2	29/05/14	850	179	3	C	2
Oit15	G2	Oct_216	G2	29/05/14	850	179	3	D	3
Oit26	G1	Oct_220	G1	27/05/14	709	143	1	A	0
Oit26	G1	Oct_220	G1	27/05/14	709	143	1	B	0
Oit26	G1	Oct_220	G1	27/05/14	709	143	1	C	0
Oit26	G1	Oct_220	G1	27/05/14	709	143	1	D	0

Pollen Recepient reference	SI Group of pollen Recipient	Pollen donor reference	SI Group of pollen donor	Pollination date	Photo reference number	photo random number	N° of flower	Repetition of flower read	Class of SI phenotype
Oit26	G1	Oct_220	G1	27/05/14	710	144	2	A	0
Oit26	G1	Oct_220	G1	27/05/14	710	144	2	B	0
Oit26	G1	Oct_220	G1	27/05/14	710	144	2	C	0
Oit26	G1	Oct_220	G1	27/05/14	710	144	2	D	0
Oit26	G1	Oct_220	G1	27/05/14	711	115	3	A	0
Oit26	G1	Oct_220	G1	27/05/14	711	115	3	B	0
Oit26	G1	Oct_220	G1	27/05/14	711	115	3	C	0
Oit26	G1	Oct_220	G1	27/05/14	711	115	3	D	0
Oit27	G1	Oct_220	G1	31/05/14	383	149	1	A	1
Oit27	G1	Oct_220	G1	31/05/14	383	149	1	B	1
Oit27	G1	Oct_220	G1	31/05/14	383	149	1	C	2
Oit27	G1	Oct_220	G1	31/05/14	383	149	1	D	1
Oit27	G1	Oct_220	G1	31/05/14	384	182	2	A	2
Oit27	G1	Oct_220	G1	31/05/14	384	182	2	B	2
Oit27	G1	Oct_220	G1	31/05/14	384	182	2	C	2
Oit27	G1	Oct_220	G1	31/05/14	384	182	2	D	2
Oit27	G1	Oct_220	G1	31/05/14	385	123	3	A	1
Oit27	G1	Oct_220	G1	31/05/14	385	123	3	B	1
Oit27	G1	Oct_220	G1	31/05/14	385	123	3	C	1
Oit27	G1	Oct_220	G1	31/05/14	385	123	3	D	2
Oit26	G1	Oct_225	G1	29/05/14	922	208	1	A	1
Oit26	G1	Oct_225	G1	29/05/14	922	208	1	B	1
Oit26	G1	Oct_225	G1	29/05/14	922	208	1	C	0
Oit26	G1	Oct_225	G1	29/05/14	922	208	1	D	0
Oit26	G1	Oct_225	G1	29/05/14	923	218	2	A	1
Oit26	G1	Oct_225	G1	29/05/14	923	218	2	B	1
Oit26	G1	Oct_225	G1	29/05/14	923	218	2	C	1
Oit26	G1	Oct_225	G1	29/05/14	923	218	2	D	2
Oit27	G1	Oct_225	G1	31/05/14	1334	327	1	A	1
Oit27	G1	Oct_225	G1	31/05/14	1334	327	1	B	0
Oit27	G1	Oct_225	G1	31/05/14	1334	327	1	C	0
Oit27	G1	Oct_225	G1	31/05/14	1334	327	1	D	1
Oit27	G1	Oct_225	G1	31/05/14	1335	355	2	A	1
Oit27	G1	Oct_225	G1	31/05/14	1335	355	2	B	1
Oit27	G1	Oct_225	G1	31/05/14	1335	355	2	C	1
Oit27	G1	Oct_225	G1	31/05/14	1335	355	2	D	1
Oit26	G1	Oct_226a	G1	29/05/14	944	227	1	A	1
Oit26	G1	Oct_226a	G1	29/05/14	944	227	1	B	0
Oit26	G1	Oct_226a	G1	29/05/14	944	227	1	C	1
Oit26	G1	Oct_226a	G1	29/05/14	944	227	1	D	0
Oit26	G1	Oct_226a	G1	29/05/14	945	212	2	A	1
Oit26	G1	Oct_226a	G1	29/05/14	945	212	2	B	1
Oit26	G1	Oct_226a	G1	29/05/14	945	212	2	C	2
Oit26	G1	Oct_226a	G1	29/05/14	945	212	2	D	2
Oit26	G1	Oct_226a	G1	29/05/14	946	239	3	A	1
Oit26	G1	Oct_226a	G1	29/05/14	946	239	3	B	1
Oit26	G1	Oct_226a	G1	29/05/14	946	239	3	C	1
Oit26	G1	Oct_226a	G1	29/05/14	946	239	3	D	2
Oit27	G1	Oct_226a	G1	31/05/14	1355	423	1	A	1
Oit27	G1	Oct_226a	G1	31/05/14	1355	423	1	B	1
Oit27	G1	Oct_226a	G1	31/05/14	1355	423	1	C	1
Oit27	G1	Oct_226a	G1	31/05/14	1355	423	1	D	2
Oit27	G1	Oct_226a	G1	31/05/14	1356	421	2	A	1
Oit27	G1	Oct_226a	G1	31/05/14	1356	421	2	B	1
Oit27	G1	Oct_226a	G1	31/05/14	1356	421	2	C	1
Oit27	G1	Oct_226a	G1	31/05/14	1356	421	2	D	2
Oit26	G1	Oct_226b	G1	29/05/14	859	180	1	A	1
Oit26	G1	Oct_226b	G1	29/05/14	859	180	1	B	1

Pollen Recepient reference	SI Group of pollen Recipient	Pollen donor reference	SI Group of pollen donor	Pollination date	Photo reference number	photo random number	N° of flower	Repetition of flower read	Class of SI phenotype
Oit26	G1	Oct_226b	G1	29/05/14	859	180	1	C	1
Oit26	G1	Oct_226b	G1	29/05/14	859	180	1	D	1
Oit26	G1	Oct_226b	G1	29/05/14	860	169	2	A	1
Oit26	G1	Oct_226b	G1	29/05/14	860	169	2	B	1
Oit26	G1	Oct_226b	G1	29/05/14	860	169	2	C	1
Oit26	G1	Oct_226b	G1	29/05/14	860	169	2	D	1
Oit26	G1	Oct_226b	G1	29/05/14	861	79	3	A	1
Oit26	G1	Oct_226b	G1	29/05/14	861	79	3	B	2
Oit26	G1	Oct_226b	G1	29/05/14	861	79	3	C	1
Oit26	G1	Oct_226b	G1	29/05/14	861	79	3	D	1
Oit27	G1	Oct_226b	G1	31/05/14	1275	372	1	A	1
Oit27	G1	Oct_226b	G1	31/05/14	1275	372	1	B	1
Oit27	G1	Oct_226b	G1	31/05/14	1275	372	1	C	1
Oit27	G1	Oct_226b	G1	31/05/14	1275	372	1	D	1
Oit27	G1	Oct_226b	G1	31/05/14	1276	319	2	A	1
Oit27	G1	Oct_226b	G1	31/05/14	1276	319	2	B	1
Oit27	G1	Oct_226b	G1	31/05/14	1276	319	2	C	1
Oit27	G1	Oct_226b	G1	31/05/14	1276	319	2	D	1
Oit27	G1	Oct_226b	G1	31/05/14	1277	398	3	A	1
Oit27	G1	Oct_226b	G1	31/05/14	1277	398	3	B	1
Oit27	G1	Oct_226b	G1	31/05/14	1277	398	3	C	1
Oit27	G1	Oct_226b	G1	31/05/14	1277	398	3	D	1
Oit26	G1	Oct_231	G1	27/05/14	703	48	1	A	1
Oit26	G1	Oct_231	G1	27/05/14	703	48	1	B	1
Oit26	G1	Oct_231	G1	27/05/14	703	48	1	C	0
Oit26	G1	Oct_231	G1	27/05/14	703	48	1	D	0
Oit26	G1	Oct_231	G1	27/05/14	704	43	2	A	1
Oit26	G1	Oct_231	G1	27/05/14	704	43	2	B	0
Oit26	G1	Oct_231	G1	27/05/14	704	43	2	C	1
Oit26	G1	Oct_231	G1	27/05/14	704	43	2	D	0
Oit26	G1	Oct_231	G1	27/05/14	705	181	3	A	1
Oit26	G1	Oct_231	G1	27/05/14	705	181	3	B	0
Oit26	G1	Oct_231	G1	27/05/14	705	181	3	C	0
Oit26	G1	Oct_231	G1	27/05/14	705	181	3	D	0
Oit27	G1	Oct_231	G1	31/05/14	401	22	1	A	1
Oit27	G1	Oct_231	G1	31/05/14	401	22	1	B	1
Oit27	G1	Oct_231	G1	31/05/14	401	22	1	C	1
Oit27	G1	Oct_231	G1	31/05/14	401	22	1	D	0
Oit27	G1	Oct_231	G1	31/05/14	402	54	2	A	1
Oit27	G1	Oct_231	G1	31/05/14	402	54	2	B	1
Oit27	G1	Oct_231	G1	31/05/14	402	54	2	C	1
Oit27	G1	Oct_231	G1	31/05/14	402	54	2	D	2
Oit27	G1	Oct_231	G1	31/05/14	403	40	3	A	1
Oit27	G1	Oct_231	G1	31/05/14	403	40	3	B	0
Oit27	G1	Oct_231	G1	31/05/14	403	40	3	C	0
Oit27	G1	Oct_231	G1	31/05/14	403	40	3	D	1
Oit30	G2	Oct_233	G2	31/05/14	1136	320	1	A	1
Oit30	G2	Oct_233	G2	31/05/14	1136	320	1	B	0
Oit30	G2	Oct_233	G2	31/05/14	1136	320	1	C	0
Oit30	G2	Oct_233	G2	31/05/14	1136	320	1	D	0
Oit30	G2	Oct_233	G2	31/05/14	1137	351	2	A	1
Oit30	G2	Oct_233	G2	31/05/14	1137	351	2	B	1
Oit30	G2	Oct_233	G2	31/05/14	1137	351	2	C	1
Oit30	G2	Oct_233	G2	31/05/14	1137	351	2	D	1
Oit26	G1	Oct_236	G1	29/05/14	950	288	1	A	0
Oit26	G1	Oct_236	G1	29/05/14	950	288	1	B	0
Oit26	G1	Oct_236	G1	29/05/14	950	288	1	C	0
Oit26	G1	Oct_236	G1	29/05/14	950	288	1	D	0

Pollen Recepient reference	SI Group of pollen Recipient	Pollen donor reference	SI Group of pollen donor	Pollination date	Photo reference number	photo random number	N° of flower	Repetition of flower read	Class of SI phenotype
Oit26	G1	Oct_236	G1	29/05/14	951	259	2	A	1
Oit26	G1	Oct_236	G1	29/05/14	951	259	2	B	1
Oit26	G1	Oct_236	G1	29/05/14	951	259	2	C	1
Oit26	G1	Oct_236	G1	29/05/14	951	259	2	D	1
Oit26	G1	Oct_236	G1	29/05/14	952	256	3	A	0
Oit26	G1	Oct_236	G1	29/05/14	952	256	3	B	0
Oit26	G1	Oct_236	G1	29/05/14	952	256	3	C	0
Oit26	G1	Oct_236	G1	29/05/14	952	256	3	D	0
Oit27	G1	Oct_236	G1	31/05/14	1359	424	1	A	1
Oit27	G1	Oct_236	G1	31/05/14	1359	424	1	B	1
Oit27	G1	Oct_236	G1	31/05/14	1359	424	1	C	1
Oit27	G1	Oct_236	G1	31/05/14	1359	424	1	D	1
Oit27	G1	Oct_236	G1	31/05/14	1360	429	2	A	1
Oit27	G1	Oct_236	G1	31/05/14	1360	429	2	B	1
Oit27	G1	Oct_236	G1	31/05/14	1360	429	2	C	2
Oit27	G1	Oct_236	G1	31/05/14	1360	429	2	D	1
Oit27	G1	Oct_236	G1	31/05/14	1361	412	3	A	1
Oit27	G1	Oct_236	G1	31/05/14	1361	412	3	B	1
Oit27	G1	Oct_236	G1	31/05/14	1361	412	3	C	1
Oit27	G1	Oct_236	G1	31/05/14	1361	412	3	D	1
Oit26	G1	Oct_240	G1	29/05/14	956	257	1	A	0
Oit26	G1	Oct_240	G1	29/05/14	956	257	1	B	0
Oit26	G1	Oct_240	G1	29/05/14	956	257	1	C	0
Oit26	G1	Oct_240	G1	29/05/14	956	257	1	D	0
Oit26	G1	Oct_240	G1	29/05/14	957	229	2	A	0
Oit26	G1	Oct_240	G1	29/05/14	957	229	2	B	0
Oit26	G1	Oct_240	G1	29/05/14	957	229	2	C	0
Oit26	G1	Oct_240	G1	29/05/14	957	229	2	D	0
Oit26	G1	Oct_240	G1	29/05/14	958	246	3	A	0
Oit26	G1	Oct_240	G1	29/05/14	958	246	3	B	0
Oit26	G1	Oct_240	G1	29/05/14	958	246	3	C	0
Oit26	G1	Oct_240	G1	29/05/14	958	246	3	D	0
Oit27	G1	Oct_240	G1	31/05/14	341	93	1	A	0
Oit27	G1	Oct_240	G1	31/05/14	341	93	1	B	0
Oit27	G1	Oct_240	G1	31/05/14	341	93	1	C	0
Oit27	G1	Oct_240	G1	31/05/14	341	93	1	D	0
Oit27	G1	Oct_240	G1	31/05/14	342	102	2	A	0
Oit27	G1	Oct_240	G1	31/05/14	342	102	2	B	0
Oit27	G1	Oct_240	G1	31/05/14	342	102	2	C	0
Oit27	G1	Oct_240	G1	31/05/14	342	102	2	D	0
Oit27	G1	Oct_240	G1	31/05/14	343	119	3	A	1
Oit27	G1	Oct_240	G1	31/05/14	343	119	3	B	1
Oit27	G1	Oct_240	G1	31/05/14	343	119	3	C	0
Oit27	G1	Oct_240	G1	31/05/14	343	119	3	D	0
Oit26	G1	Oct_241	G1	27/05/14	662	159	1	A	1
Oit26	G1	Oct_241	G1	27/05/14	662	159	1	B	1
Oit26	G1	Oct_241	G1	27/05/14	662	159	1	C	1
Oit26	G1	Oct_241	G1	27/05/14	662	159	1	D	1
Oit26	G1	Oct_241	G1	27/05/14	663	75	2	A	1
Oit26	G1	Oct_241	G1	27/05/14	663	75	2	B	1
Oit26	G1	Oct_241	G1	27/05/14	663	75	2	C	1
Oit26	G1	Oct_241	G1	27/05/14	663	75	2	D	1
Oit26	G1	Oct_241	G1	27/05/14	665	176	4	A	1
Oit26	G1	Oct_241	G1	27/05/14	665	176	4	B	1
Oit26	G1	Oct_241	G1	27/05/14	665	176	4	C	1
Oit26	G1	Oct_241	G1	27/05/14	665	176	4	D	1
Oit27	G1	Oct_241	G1	31/05/14	1106	252	1	A	2
Oit27	G1	Oct_241	G1	31/05/14	1106	252	1	B	1

Pollen Recepient reference	SI Group of pollen Recipient	Pollen donor reference	SI Group of pollen donor	Pollination date	Photo reference number	photo random number	N° of flower	Repetition of flower read	Class of SI phenotype
Oit27	G1	Oct_241	G1	31/05/14	1106	252	1	C	1
Oit27	G1	Oct_241	G1	31/05/14	1106	252	1	D	1
Oit27	G1	Oct_241	G1	31/05/14	1107	248	2	A	0
Oit27	G1	Oct_241	G1	31/05/14	1107	248	2	B	0
Oit27	G1	Oct_241	G1	31/05/14	1107	248	2	C	0
Oit27	G1	Oct_241	G1	31/05/14	1107	248	2	D	0
Oit27	G1	Oct_241	G1	31/05/14	1108	211	3	A	1
Oit27	G1	Oct_241	G1	31/05/14	1108	211	3	B	1
Oit27	G1	Oct_241	G1	31/05/14	1108	211	3	C	1
Oit27	G1	Oct_241	G1	31/05/14	1108	211	3	D	1
Oit26	G1	Oct_243	G1	29/05/14	933	282	1	A	0
Oit26	G1	Oct_243	G1	29/05/14	933	282	1	B	0
Oit26	G1	Oct_243	G1	29/05/14	933	282	1	C	0
Oit26	G1	Oct_243	G1	29/05/14	933	282	1	D	0
Oit26	G1	Oct_243	G1	29/05/14	934	214	2	A	0
Oit26	G1	Oct_243	G1	29/05/14	934	214	2	B	0
Oit26	G1	Oct_243	G1	29/05/14	934	214	2	C	0
Oit26	G1	Oct_243	G1	29/05/14	934	214	2	D	0
Oit27	G1	Oct_243	G1	31/05/14	1344	431	1	A	1
Oit27	G1	Oct_243	G1	31/05/14	1344	431	1	B	0
Oit27	G1	Oct_243	G1	31/05/14	1344	431	1	C	0
Oit27	G1	Oct_243	G1	31/05/14	1344	431	1	D	0
Oit27	G1	Oct_243	G1	31/05/14	1345	440	2	A	1
Oit27	G1	Oct_243	G1	31/05/14	1345	440	2	B	1
Oit27	G1	Oct_243	G1	31/05/14	1345	440	2	C	0
Oit27	G1	Oct_243	G1	31/05/14	1345	440	2	D	1
Oit27	G1	Oct_243	G1	31/05/14	1346	413	3	A	0
Oit27	G1	Oct_243	G1	31/05/14	1346	413	3	B	0
Oit27	G1	Oct_243	G1	31/05/14	1346	413	3	C	0
Oit27	G1	Oct_243	G1	31/05/14	1346	413	3	D	1
Oit30	G2	Oct_247	G2	31/05/14	419	5	1	A	2
Oit30	G2	Oct_247	G2	31/05/14	419	5	1	B	2
Oit30	G2	Oct_247	G2	31/05/14	419	5	1	C	2
Oit30	G2	Oct_247	G2	31/05/14	419	5	1	D	2
Oit30	G2	Oct_247	G2	31/05/14	420	198	2	A	2
Oit30	G2	Oct_247	G2	31/05/14	420	198	2	B	2
Oit30	G2	Oct_247	G2	31/05/14	420	198	2	C	2
Oit30	G2	Oct_247	G2	31/05/14	420	198	2	D	2
Oit30	G2	Oct_247	G2	31/05/14	421	106	3	A	1
Oit30	G2	Oct_247	G2	31/05/14	421	106	3	B	3
Oit30	G2	Oct_247	G2	31/05/14	421	106	3	C	2
Oit30	G2	Oct_247	G2	31/05/14	421	106	3	D	2
Oit15	G2	Oct_247	G2	27/05/14	682	50	1	A	3
Oit15	G2	Oct_247	G2	27/05/14	682	50	1	B	3
Oit15	G2	Oct_247	G2	27/05/14	682	50	1	C	3
Oit15	G2	Oct_247	G2	27/05/14	682	50	1	D	3
Oit15	G2	Oct_247	G2	27/05/14	683	142	2	A	2
Oit15	G2	Oct_247	G2	27/05/14	683	142	2	B	3
Oit15	G2	Oct_247	G2	27/05/14	683	142	2	C	2
Oit15	G2	Oct_247	G2	27/05/14	683	142	2	D	3
Oit15	G2	Oct_247	G2	27/05/14	684	44	3	A	2
Oit15	G2	Oct_247	G2	27/05/14	684	44	3	B	2
Oit15	G2	Oct_247	G2	27/05/14	684	44	3	C	3
Oit15	G2	Oct_247	G2	27/05/14	684	44	3	D	3
Oit30	G2	Oct_251	G2	31/05/02	356	98	1	A	2
Oit30	G2	Oct_251	G2	31/05/02	356	98	1	B	2
Oit30	G2	Oct_251	G2	31/05/02	356	98	1	C	3
Oit30	G2	Oct_251	G2	31/05/02	356	98	1	D	2

Pollen Recepient reference	SI Group of pollen Recipient	Pollen donor reference	SI Group of pollen donor	Pollination date	Photo reference number	photo random number	N° of flower	Repetition of flower read	Class of SI phenotype
Oit30	G2	Oct_251	G2	31/05/02	357	78	2	A	2
Oit30	G2	Oct_251	G2	31/05/02	357	78	2	B	2
Oit30	G2	Oct_251	G2	31/05/02	357	78	2	C	2
Oit30	G2	Oct_251	G2	31/05/02	357	78	2	D	2
Oit30	G2	Oct_251	G2	31/05/02	358	177	3	A	0
Oit30	G2	Oct_251	G2	31/05/02	358	177	3	B	0
Oit30	G2	Oct_251	G2	31/05/02	358	177	3	C	0
Oit30	G2	Oct_251	G2	31/05/02	358	177	3	D	0
Oit30	G2	Oct_251	G2	31/05/14	1131	203	1	A	2
Oit30	G2	Oct_251	G2	31/05/14	1131	203	1	B	2
Oit30	G2	Oct_251	G2	31/05/14	1131	203	1	C	2
Oit30	G2	Oct_251	G2	31/05/14	1131	203	1	D	2
Oit30	G2	Oct_251	G2	31/05/14	1133	249	2	A	1
Oit30	G2	Oct_251	G2	31/05/14	1133	249	2	B	2
Oit30	G2	Oct_251	G2	31/05/14	1133	249	2	C	2
Oit30	G2	Oct_251	G2	31/05/14	1133	249	2	D	2
Oit15	G2	Oct_251	G2	29/05/14	971	280	1	A	2
Oit15	G2	Oct_251	G2	29/05/14	971	280	1	B	3
Oit15	G2	Oct_251	G2	29/05/14	971	280	1	C	3
Oit15	G2	Oct_251	G2	29/05/14	971	280	1	D	3
Oit15	G2	Oct_251	G2	29/05/14	972	209	2	A	4
Oit15	G2	Oct_251	G2	29/05/14	972	209	2	B	5
Oit15	G2	Oct_251	G2	29/05/14	972	209	2	C	5
Oit15	G2	Oct_251	G2	29/05/14	972	209	2	D	5
Oit15	G2	Oct_251	G2	29/05/14	973	300	3	A	2
Oit15	G2	Oct_251	G2	29/05/14	973	300	3	B	2
Oit15	G2	Oct_251	G2	29/05/14	973	300	3	C	2
Oit15	G2	Oct_251	G2	29/05/14	973	300	3	D	2
Oit30	G2	Oct_256	G2	31/05/14	392	67	1	A	2
Oit30	G2	Oct_256	G2	31/05/14	392	67	1	B	2
Oit30	G2	Oct_256	G2	31/05/14	392	67	1	C	3
Oit30	G2	Oct_256	G2	31/05/14	392	67	1	D	3
Oit30	G2	Oct_256	G2	31/05/14	393	81	2	A	2
Oit30	G2	Oct_256	G2	31/05/14	393	81	2	B	2
Oit30	G2	Oct_256	G2	31/05/14	393	81	2	C	2
Oit30	G2	Oct_256	G2	31/05/14	393	81	2	D	2
Oit30	G2	Oct_256	G2	31/05/14	394	131	3	A	0
Oit30	G2	Oct_256	G2	31/05/14	394	131	3	B	0
Oit30	G2	Oct_256	G2	31/05/14	394	131	3	C	0
Oit30	G2	Oct_256	G2	31/05/14	394	131	3	D	0
Oit15	G2	Oct_256	G2	27/05/02	180	101	3	A	3
Oit15	G2	Oct_256	G2	27/05/02	180	101	3	B	3
Oit15	G2	Oct_256	G2	27/05/02	180	101	3	C	4
Oit15	G2	Oct_256	G2	27/05/02	180	101	3	D	4
Oit15	G2	Oct_256	G2	27/05/02	181	162	4	A	3
Oit15	G2	Oct_256	G2	27/05/02	181	162	4	B	4
Oit15	G2	Oct_256	G2	27/05/02	181	162	4	C	4
Oit15	G2	Oct_256	G2	27/05/02	181	162	4	D	3
Oit15	G2	Oct_256	G2	27/05/14	641	39	1	A	3
Oit15	G2	Oct_256	G2	27/05/14	641	39	1	B	3
Oit15	G2	Oct_256	G2	27/05/14	641	39	1	C	3
Oit15	G2	Oct_256	G2	27/05/14	641	39	1	D	4
Oit15	G2	Oct_256	G2	27/05/14	642	116	2	A	2
Oit15	G2	Oct_256	G2	27/05/14	642	116	2	B	3
Oit15	G2	Oct_256	G2	27/05/14	642	116	2	C	5
Oit15	G2	Oct_256	G2	27/05/14	642	116	2	D	3
Oit15	G2	Oct_256	G2	27/05/14	643	83	3	A	3
Oit15	G2	Oct_256	G2	27/05/14	643	83	3	B	4

Pollen Recepient reference	SI Group of pollen Recipient	Pollen donor reference	SI Group of pollen donor	Pollination date	Photo reference number	photo random number	N° of flower	Repetition of flower read	Class of SI phenotype
Oit15	G2	Oct_256	G2	27/05/14	643	83	3	C	4
Oit15	G2	Oct_256	G2	27/05/14	643	83	3	D	4
Oit30	G2	Oct_267	G2	31/05/14	1245	305	1	A	1
Oit30	G2	Oct_267	G2	31/05/14	1245	305	1	B	2
Oit30	G2	Oct_267	G2	31/05/14	1245	305	1	C	2
Oit30	G2	Oct_267	G2	31/05/14	1245	305	1	D	2
Oit30	G2	Oct_267	G2	31/05/14	1246	389	2	A	0
Oit30	G2	Oct_267	G2	31/05/14	1246	389	2	B	0
Oit30	G2	Oct_267	G2	31/05/14	1246	389	2	C	0
Oit30	G2	Oct_267	G2	31/05/14	1246	389	2	D	0
Oit30	G2	Oct_267	G2	31/05/14	1247	316	3	A	1
Oit30	G2	Oct_267	G2	31/05/14	1247	316	3	B	1
Oit30	G2	Oct_267	G2	31/05/14	1247	316	3	C	0
Oit30	G2	Oct_267	G2	31/05/14	1247	316	3	D	2
Oit15	G2	Oct_267	G2	27/05/14	782	56	1	A	2
Oit15	G2	Oct_267	G2	27/05/14	782	56	1	B	2
Oit15	G2	Oct_267	G2	27/05/14	782	56	1	C	2
Oit15	G2	Oct_267	G2	27/05/14	782	56	1	D	2
Oit15	G2	Oct_267	G2	27/05/14	783	170	2	A	0
Oit15	G2	Oct_267	G2	27/05/14	783	170	2	B	0
Oit15	G2	Oct_267	G2	27/05/14	783	170	2	C	0
Oit15	G2	Oct_267	G2	27/05/14	783	170	2	D	0
Oit15	G2	Oct_267	G2	27/05/14	784	187	3	A	1
Oit15	G2	Oct_267	G2	27/05/14	784	187	3	B	1
Oit15	G2	Oct_267	G2	27/05/14	784	187	3	C	3
Oit15	G2	Oct_267	G2	27/05/14	784	187	3	D	3
Oit30	G2	Oct_268	G2	31/05/14	1233	370	1	A	0
Oit30	G2	Oct_268	G2	31/05/14	1233	370	1	B	0
Oit30	G2	Oct_268	G2	31/05/14	1233	370	1	C	0
Oit30	G2	Oct_268	G2	31/05/14	1233	370	1	D	0
Oit30	G2	Oct_268	G2	31/05/14	1234	373	2	A	1
Oit30	G2	Oct_268	G2	31/05/14	1234	373	2	B	1
Oit30	G2	Oct_268	G2	31/05/14	1234	373	2	C	1
Oit30	G2	Oct_268	G2	31/05/14	1234	373	2	D	2
Oit15	G2	Oct_268	G2	27/05/14	771	76	1	A	2
Oit15	G2	Oct_268	G2	27/05/14	771	76	1	B	2
Oit15	G2	Oct_268	G2	27/05/14	771	76	1	C	3
Oit15	G2	Oct_268	G2	27/05/14	771	76	1	D	3
Oit15	G2	Oct_268	G2	27/05/14	772	33	2	A	3
Oit15	G2	Oct_268	G2	27/05/14	772	33	2	B	2
Oit15	G2	Oct_268	G2	27/05/14	772	33	2	C	1
Oit15	G2	Oct_268	G2	27/05/14	772	33	2	D	0
Oit15	G2	Oct_268	G2	27/05/14	773	121	3	A	1
Oit15	G2	Oct_268	G2	27/05/14	773	121	3	B	0
Oit15	G2	Oct_268	G2	27/05/14	773	121	3	C	0
Oit15	G2	Oct_268	G2	27/05/14	773	121	3	D	0
Oit15	G2	Oct_271	G2	27/05/14	196	140	1	A	1
Oit15	G2	Oct_271	G2	27/05/14	196	140	1	B	1
Oit15	G2	Oct_271	G2	27/05/14	196	140	1	C	2
Oit15	G2	Oct_271	G2	27/05/14	196	140	1	D	2
Oit15	G2	Oct_271	G2	27/05/14	197	100	2	A	2
Oit15	G2	Oct_271	G2	27/05/14	197	100	2	B	2
Oit15	G2	Oct_271	G2	27/05/14	197	100	2	C	2
Oit15	G2	Oct_271	G2	27/05/14	197	100	2	D	2
Oit15	G2	Oct_271	G2	27/05/14	198	114	3	A	1
Oit15	G2	Oct_271	G2	27/05/14	198	114	3	B	2
Oit15	G2	Oct_271	G2	27/05/14	198	114	3	C	2
Oit15	G2	Oct_271	G2	27/05/14	198	114	3	D	2

Pollen Recepient reference	SI Group of pollen Recipient	Pollen donor reference	SI Group of pollen donor	Pollination date	Photo reference number	photo random number	N° of flower	Repetition of flower read	Class of SI phenotype
Oit15	G2	Oct_271	G2	27/05/02	750	8	1	A	3
Oit15	G2	Oct_271	G2	27/05/02	750	8	1	B	1
Oit15	G2	Oct_271	G2	27/05/02	750	8	1	C	0
Oit15	G2	Oct_271	G2	27/05/02	750	8	1	D	0
Oit15	G2	Oct_271	G2	27/05/02	751	82	2	A	2
Oit15	G2	Oct_271	G2	27/05/02	751	82	2	B	2
Oit15	G2	Oct_271	G2	27/05/02	751	82	2	C	1
Oit15	G2	Oct_271	G2	27/05/02	751	82	2	D	3
Oit15	G2	Oct_271	G2	27/05/02	752	164	3	A	2
Oit15	G2	Oct_271	G2	27/05/02	752	164	3	B	3
Oit15	G2	Oct_271	G2	27/05/02	752	164	3	C	4
Oit15	G2	Oct_271	G2	27/05/02	752	164	3	D	3
Oit26	G1	Oct_273	G1	29/05/14	865	117	1	A	1
Oit26	G1	Oct_273	G1	29/05/14	865	117	1	B	1
Oit26	G1	Oct_273	G1	29/05/14	865	117	1	C	0
Oit26	G1	Oct_273	G1	29/05/14	865	117	1	D	2
Oit26	G1	Oct_273	G1	29/05/14	866	124	2	A	1
Oit26	G1	Oct_273	G1	29/05/14	866	124	2	B	1
Oit26	G1	Oct_273	G1	29/05/14	866	124	2	C	1
Oit26	G1	Oct_273	G1	29/05/14	866	124	2	D	1
Oit27	G1	Oct_273	G1	31/05/14	1281	322	1	A	1
Oit27	G1	Oct_273	G1	31/05/14	1281	322	1	B	1
Oit27	G1	Oct_273	G1	31/05/14	1281	322	1	C	1
Oit27	G1	Oct_273	G1	31/05/14	1281	322	1	D	1
Oit27	G1	Oct_273	G1	31/05/14	1282	366	2	A	2
Oit27	G1	Oct_273	G1	31/05/14	1282	366	2	B	2
Oit27	G1	Oct_273	G1	31/05/14	1282	366	2	C	2
Oit27	G1	Oct_273	G1	31/05/14	1282	366	2	D	3
Oit30	G2	Oct_275	G2	31/05/14	1074	264	1	A	1
Oit30	G2	Oct_275	G2	31/05/14	1074	264	1	B	2
Oit30	G2	Oct_275	G2	31/05/14	1074	264	1	C	2
Oit30	G2	Oct_275	G2	31/05/14	1074	264	1	D	2
Oit30	G2	Oct_275	G2	31/05/14	1075	270	2	A	2
Oit30	G2	Oct_275	G2	31/05/14	1075	270	2	B	2
Oit30	G2	Oct_275	G2	31/05/14	1075	270	2	C	2
Oit30	G2	Oct_275	G2	31/05/14	1075	270	2	D	2
Oit30	G2	Oct_275	G2	31/05/14	1076	251	3	A	2
Oit30	G2	Oct_275	G2	31/05/14	1076	251	3	B	2
Oit30	G2	Oct_275	G2	31/05/14	1076	251	3	C	2
Oit30	G2	Oct_275	G2	31/05/14	1076	251	3	D	2
Oit15	G2	Oct_275	G2	27/05/14	666	192	1	A	4
Oit15	G2	Oct_275	G2	27/05/14	666	192	1	B	8
Oit15	G2	Oct_275	G2	27/05/14	666	192	1	C	8
Oit15	G2	Oct_275	G2	27/05/14	666	192	1	D	6
Oit15	G2	Oct_275	G2	27/05/14	667	70	2	A	3
Oit15	G2	Oct_275	G2	27/05/14	667	70	2	B	3
Oit15	G2	Oct_275	G2	27/05/14	667	70	2	C	3
Oit15	G2	Oct_275	G2	27/05/14	667	70	2	D	3
Oit15	G2	Oct_275	G2	27/05/14	668	108	3	A	3
Oit15	G2	Oct_275	G2	27/05/14	668	108	3	B	3
Oit15	G2	Oct_275	G2	27/05/14	668	108	3	C	3
Oit15	G2	Oct_275	G2	27/05/14	668	108	3	D	3
Oit15	G2	Oct_315	G2	29/05/14	982	220	1	A	3
Oit15	G2	Oct_315	G2	29/05/14	982	220	1	B	3
Oit15	G2	Oct_315	G2	29/05/14	982	220	1	C	4
Oit15	G2	Oct_315	G2	29/05/14	982	220	1	D	3
Oit15	G2	Oct_315	G2	29/05/14	983	268	2	A	3
Oit15	G2	Oct_315	G2	29/05/14	983	268	2	B	3

Pollen Recepient reference	SI Group of pollen Recipient	Pollen donor reference	SI Group of pollen donor	Pollination date	Photo reference number	photo random number	N° of flower	Repetition of flower read	Class of SI phenotype
Oit15	G2	Oct_315	G2	29/05/14	983	268	2	C	3
Oit15	G2	Oct_315	G2	29/05/14	983	268	2	D	4
Oit15	G2	Oct_315	G2	29/05/14	984	201	3	A	3
Oit15	G2	Oct_315	G2	29/05/14	984	201	3	B	3
Oit15	G2	Oct_315	G2	29/05/14	984	201	3	C	3
Oit15	G2	Oct_315	G2	29/05/14	984	201	3	D	4
Oit30	G2	Oct_345	G2	31/05/14	1347	411	1	A	2
Oit30	G2	Oct_345	G2	31/05/14	1347	411	1	B	2
Oit30	G2	Oct_345	G2	31/05/14	1347	411	1	C	3
Oit30	G2	Oct_345	G2	31/05/14	1347	411	1	D	3
Oit30	G2	Oct_345	G2	31/05/14	1348	407	2	A	2
Oit30	G2	Oct_345	G2	31/05/14	1348	407	2	B	2
Oit30	G2	Oct_345	G2	31/05/14	1348	407	2	C	3
Oit30	G2	Oct_345	G2	31/05/14	1348	407	2	D	2
Oit30	G2	Oct_345	G2	31/05/14	1349	408	3	A	1
Oit30	G2	Oct_345	G2	31/05/14	1349	408	3	B	1
Oit30	G2	Oct_345	G2	31/05/14	1349	408	3	C	0
Oit30	G2	Oct_345	G2	31/05/14	1349	408	3	D	0
Oit15	G2	Oct_345	G2	29/05/14	938	217	1	A	2
Oit15	G2	Oct_345	G2	29/05/14	938	217	1	B	4
Oit15	G2	Oct_345	G2	29/05/14	938	217	1	C	4
Oit15	G2	Oct_345	G2	29/05/14	938	217	1	D	4
Oit15	G2	Oct_345	G2	29/05/14	939	284	2	A	2
Oit15	G2	Oct_345	G2	29/05/14	939	284	2	B	3
Oit15	G2	Oct_345	G2	29/05/14	939	284	2	C	3
Oit15	G2	Oct_345	G2	29/05/14	939	284	2	D	3
Oit15	G2	Oct_345	G2	29/05/14	940	263	3	A	2
Oit15	G2	Oct_345	G2	29/05/14	940	263	3	B	2
Oit15	G2	Oct_345	G2	29/05/14	940	263	3	C	2
Oit15	G2	Oct_345	G2	29/05/14	940	263	3	D	2
Oit30	G2	Oct_347	G2	31/05/14	1109	202	1	A	1
Oit30	G2	Oct_347	G2	31/05/14	1109	202	1	B	1
Oit30	G2	Oct_347	G2	31/05/14	1109	202	1	C	2
Oit30	G2	Oct_347	G2	31/05/14	1109	202	1	D	2
Oit30	G2	Oct_347	G2	31/05/14	1110	226	2	A	2
Oit30	G2	Oct_347	G2	31/05/14	1110	226	2	B	2
Oit30	G2	Oct_347	G2	31/05/14	1110	226	2	C	2
Oit30	G2	Oct_347	G2	31/05/14	1110	226	2	D	2
Oit30	G2	Oct_347	G2	31/05/14	1111	291	3	A	1
Oit30	G2	Oct_347	G2	31/05/14	1111	291	3	B	2
Oit30	G2	Oct_347	G2	31/05/14	1111	291	3	C	2
Oit30	G2	Oct_347	G2	31/05/14	1111	291	3	D	2
Oit15	G2	Oct_347	G2	27/05/14	630	199	1	A	2
Oit15	G2	Oct_347	G2	27/05/14	630	199	1	B	2
Oit15	G2	Oct_347	G2	27/05/14	630	199	1	C	3
Oit15	G2	Oct_347	G2	27/05/14	630	199	1	D	3
Oit15	G2	Oct_347	G2	27/05/14	631	150	2	A	1
Oit15	G2	Oct_347	G2	27/05/14	631	150	2	B	2
Oit15	G2	Oct_347	G2	27/05/14	631	150	2	C	2
Oit15	G2	Oct_347	G2	27/05/14	631	150	2	D	2
Oit15	G2	Oct_347	G2	27/05/14	632	21	3	A	3
Oit15	G2	Oct_347	G2	27/05/14	632	21	3	B	2
Oit15	G2	Oct_347	G2	27/05/14	632	21	3	C	2
Oit15	G2	Oct_347	G2	27/05/14	632	21	3	D	2
Oit30	G2	Oct_348	G2	31/05/14	350	163	1	A	2
Oit30	G2	Oct_348	G2	31/05/14	350	163	1	B	1
Oit30	G2	Oct_348	G2	31/05/14	350	163	1	C	2
Oit30	G2	Oct_348	G2	31/05/14	350	163	1	D	2

Pollen Recepient reference	SI Group of pollen Recipient	Pollen donor reference	SI Group of pollen donor	Pollination date	Photo reference number	photo random number	N° of flower	Repetition of flower read	Class of SI phenotype
Oit30	G2	Oct_348	G2	31/05/14	351	65	2	A	1
Oit30	G2	Oct_348	G2	31/05/14	351	65	2	B	1
Oit30	G2	Oct_348	G2	31/05/14	351	65	2	C	2
Oit30	G2	Oct_348	G2	31/05/14	351	65	2	D	2
Oit30	G2	Oct_348	G2	31/05/14	352	41	3	A	2
Oit30	G2	Oct_348	G2	31/05/14	352	41	3	B	2
Oit30	G2	Oct_348	G2	31/05/14	352	41	3	C	2
Oit30	G2	Oct_348	G2	31/05/14	352	41	3	D	2
Oit15	G2	Oct_348	G2	29/05/14	965	250	1	A	3
Oit15	G2	Oct_348	G2	29/05/14	965	250	1	B	3
Oit15	G2	Oct_348	G2	29/05/14	965	250	1	C	3
Oit15	G2	Oct_348	G2	29/05/14	965	250	1	D	3
Oit15	G2	Oct_348	G2	29/05/14	966	294	2	A	2
Oit15	G2	Oct_348	G2	29/05/14	966	294	2	B	2
Oit15	G2	Oct_348	G2	29/05/14	966	294	2	C	3
Oit15	G2	Oct_348	G2	29/05/14	966	294	2	D	3
Oit15	G2	Oct_348	G2	29/05/14	967	236	3	A	2
Oit15	G2	Oct_348	G2	29/05/14	967	236	3	B	3
Oit15	G2	Oct_348	G2	29/05/14	967	236	3	C	2
Oit15	G2	Oct_348	G2	29/05/14	967	236	3	D	3
Oit26	G1	Oct_350	G1	27/05/14	679	97	1	A	0
Oit26	G1	Oct_350	G1	27/05/14	679	97	1	B	0
Oit26	G1	Oct_350	G1	27/05/14	679	97	1	C	0
Oit26	G1	Oct_350	G1	27/05/14	679	97	1	D	0
Oit26	G1	Oct_350	G1	27/05/14	680	68	2	A	0
Oit26	G1	Oct_350	G1	27/05/14	680	68	2	B	0
Oit26	G1	Oct_350	G1	27/05/14	680	68	2	C	0
Oit26	G1	Oct_350	G1	27/05/14	680	68	2	D	0
Oit26	G1	Oct_350	G1	27/05/14	681	186	3	A	0
Oit26	G1	Oct_350	G1	27/05/14	681	186	3	B	0
Oit26	G1	Oct_350	G1	27/05/14	681	186	3	C	0
Oit26	G1	Oct_350	G1	27/05/14	681	186	3	D	0
Oit27	G1	Oct_350	G1	31/05/14	1089	261	1	A	0
Oit27	G1	Oct_350	G1	31/05/14	1089	261	1	B	0
Oit27	G1	Oct_350	G1	31/05/14	1089	261	1	C	0
Oit27	G1	Oct_350	G1	31/05/14	1089	261	1	D	0
Oit27	G1	Oct_350	G1	31/05/14	1090	235	2	A	0
Oit27	G1	Oct_350	G1	31/05/14	1090	235	2	B	0
Oit27	G1	Oct_350	G1	31/05/14	1090	235	2	C	0
Oit27	G1	Oct_350	G1	31/05/14	1090	235	2	D	0
Oit26	G1	Oct_358	G1	27/05/14	649	10	1	A	1
Oit26	G1	Oct_358	G1	27/05/14	649	10	1	B	1
Oit26	G1	Oct_358	G1	27/05/14	649	10	1	C	0
Oit26	G1	Oct_358	G1	27/05/14	649	10	1	D	0
Oit26	G1	Oct_358	G1	27/05/14	650	141	2	A	0
Oit26	G1	Oct_358	G1	27/05/14	650	141	2	B	0
Oit26	G1	Oct_358	G1	27/05/14	650	141	2	C	0
Oit26	G1	Oct_358	G1	27/05/14	650	141	2	D	0
Oit27	G1	Oct_358	G1	31/05/14	1094	274	1	A	1
Oit27	G1	Oct_358	G1	31/05/14	1094	274	1	B	0
Oit27	G1	Oct_358	G1	31/05/14	1094	274	1	C	0
Oit27	G1	Oct_358	G1	31/05/14	1094	274	1	D	1
Oit27	G1	Oct_358	G1	31/05/14	1095	260	2	A	1
Oit27	G1	Oct_358	G1	31/05/14	1095	260	2	B	0
Oit27	G1	Oct_358	G1	31/05/14	1095	260	2	C	0
Oit27	G1	Oct_358	G1	31/05/14	1095	260	2	D	1
Oit27	G1	Oct_358	G1	31/05/14	1096	275	3	A	1
Oit27	G1	Oct_358	G1	31/05/14	1096	275	3	B	1

Pollen Recepient reference	SI Group of pollen Recipient	Pollen donor reference	SI Group of pollen donor	Pollination date	Photo reference number	photo random number	N° of flower	Repetition of flower read	Class of SI phenotype
Oit27	G1	Oct_358	G1	31/05/14	1096	275	3	C	0
Oit27	G1	Oct_358	G1	31/05/14	1096	275	3	D	1
Oit26	G1	Oct_413	G1	29/05/14	895	277	1	A	1
Oit26	G1	Oct_413	G1	29/05/14	895	277	1	B	1
Oit26	G1	Oct_413	G1	29/05/14	895	277	1	C	1
Oit26	G1	Oct_413	G1	29/05/14	895	277	1	D	1
Oit26	G1	Oct_413	G1	29/05/14	896	278	2	A	1
Oit26	G1	Oct_413	G1	29/05/14	896	278	2	B	2
Oit26	G1	Oct_413	G1	29/05/14	896	278	2	C	2
Oit26	G1	Oct_413	G1	29/05/14	896	278	2	D	2
Oit26	G1	Oct_413	G1	29/05/14	897	397	3	A	1
Oit26	G1	Oct_413	G1	29/05/14	897	397	3	B	1
Oit26	G1	Oct_413	G1	29/05/14	897	397	3	C	1
Oit26	G1	Oct_413	G1	29/05/14	897	397	3	D	1
Oit27	G1	Oct_413	G1	31/05/14	1307	400	1	A	2
Oit27	G1	Oct_413	G1	31/05/14	1307	400	1	B	2
Oit27	G1	Oct_413	G1	31/05/14	1307	400	1	C	2
Oit27	G1	Oct_413	G1	31/05/14	1307	400	1	D	2
Oit27	G1	Oct_413	G1	31/05/14	1308	357	2	A	2
Oit27	G1	Oct_413	G1	31/05/14	1308	357	2	B	3
Oit27	G1	Oct_413	G1	31/05/14	1308	357	2	C	3
Oit27	G1	Oct_413	G1	31/05/14	1308	357	2	D	3
Oit27	G1	Oct_413	G1	31/05/14	1309	377	3	A	1
Oit27	G1	Oct_413	G1	31/05/14	1309	377	3	B	1
Oit27	G1	Oct_413	G1	31/05/14	1309	377	3	C	2
Oit27	G1	Oct_413	G1	31/05/14	1309	377	3	D	2
Oit26	G1	Oct_416	G1	27/05/14	214	74	1	A	0
Oit26	G1	Oct_416	G1	27/05/14	214	74	1	B	0
Oit26	G1	Oct_416	G1	27/05/14	214	74	1	C	0
Oit26	G1	Oct_416	G1	27/05/14	214	74	1	D	0
Oit26	G1	Oct_416	G1	27/05/14	215	35	2	A	2
Oit26	G1	Oct_416	G1	27/05/14	215	35	2	B	3
Oit26	G1	Oct_416	G1	27/05/14	215	35	2	C	3
Oit26	G1	Oct_416	G1	27/05/14	215	35	2	D	3
Oit26	G1	Oct_416	G1	27/05/14	216	160	3	A	1
Oit26	G1	Oct_416	G1	27/05/14	216	160	3	B	0
Oit26	G1	Oct_416	G1	27/05/14	216	160	3	C	0
Oit26	G1	Oct_416	G1	27/05/14	216	160	3	D	1
Oit27	G1	Oct_416	G1	31/05/14	1177	362	1	A	1
Oit27	G1	Oct_416	G1	31/05/14	1177	362	1	B	1
Oit27	G1	Oct_416	G1	31/05/14	1177	362	1	C	1
Oit27	G1	Oct_416	G1	31/05/14	1177	362	1	D	1
Oit27	G1	Oct_416	G1	31/05/14	1178	381	2	A	0
Oit27	G1	Oct_416	G1	31/05/14	1178	381	2	B	0
Oit27	G1	Oct_416	G1	31/05/14	1178	381	2	C	0
Oit27	G1	Oct_416	G1	31/05/14	1178	381	2	D	0
Oit27	G1	Oct_416	G1	31/05/14	1179	326	3	A	1
Oit27	G1	Oct_416	G1	31/05/14	1179	326	3	B	1
Oit27	G1	Oct_416	G1	31/05/14	1179	326	3	C	2
Oit27	G1	Oct_416	G1	31/05/14	1179	326	3	D	1
Oit26	G1	Oct_421	G1	29/05/14	911	243	1	A	1
Oit26	G1	Oct_421	G1	29/05/14	911	243	1	B	1
Oit26	G1	Oct_421	G1	29/05/14	911	243	1	C	1
Oit26	G1	Oct_421	G1	29/05/14	911	243	1	D	0
Oit26	G1	Oct_421	G1	29/05/14	912	293	2	A	1
Oit26	G1	Oct_421	G1	29/05/14	912	293	2	B	2
Oit26	G1	Oct_421	G1	29/05/14	912	293	2	C	0
Oit26	G1	Oct_421	G1	29/05/14	912	293	2	D	2

Pollen Recepient reference	SI Group of pollen Recipient	Pollen donor reference	SI Group of pollen donor	Pollination date	Photo reference number	photo random number	N° of flower	Repetition of flower read	Class of SI phenotype
Oit26	G1	Oct_421	G1	29/05/14	913	206	3	A	2
Oit26	G1	Oct_421	G1	29/05/14	913	206	3	B	0
Oit26	G1	Oct_421	G1	29/05/14	913	206	3	C	0
Oit26	G1	Oct_421	G1	29/05/14	913	206	3	D	0
Oit27	G1	Oct_421	G1	31/05/14	1323	329	1	A	1
Oit27	G1	Oct_421	G1	31/05/14	1323	329	1	B	1
Oit27	G1	Oct_421	G1	31/05/14	1323	329	1	C	1
Oit27	G1	Oct_421	G1	31/05/14	1323	329	1	D	1
Oit27	G1	Oct_421	G1	31/05/14	1324	388	2	A	2
Oit27	G1	Oct_421	G1	31/05/14	1324	388	2	B	2
Oit27	G1	Oct_421	G1	31/05/14	1324	388	2	C	2
Oit27	G1	Oct_421	G1	31/05/14	1324	388	2	D	2
Oit27	G1	Oct_421	G1	31/05/14	1325	384	3	A	1
Oit27	G1	Oct_421	G1	31/05/14	1325	384	3	B	1
Oit27	G1	Oct_421	G1	31/05/14	1325	384	3	C	0
Oit27	G1	Oct_421	G1	31/05/14	1325	384	3	D	1
Oit26	G1	Oct_480	G1	27/05/14	727	62	1	A	1
Oit26	G1	Oct_480	G1	27/05/14	727	62	1	B	1
Oit26	G1	Oct_480	G1	27/05/14	727	62	1	C	1
Oit26	G1	Oct_480	G1	27/05/14	727	62	1	D	2
Oit26	G1	Oct_480	G1	27/05/14	728	168	2	A	1
Oit26	G1	Oct_480	G1	27/05/14	728	168	2	B	1
Oit26	G1	Oct_480	G1	27/05/14	728	168	2	C	1
Oit26	G1	Oct_480	G1	27/05/14	728	168	2	D	0
Oit26	G1	Oct_480	G1	27/05/14	729	52	3	A	0
Oit26	G1	Oct_480	G1	27/05/14	729	52	3	B	0
Oit26	G1	Oct_480	G1	27/05/14	729	52	3	C	0
Oit26	G1	Oct_480	G1	27/05/14	729	52	3	D	0
Oit27	G1	Oct_480	G1	31/05/14	1189	375	1	A	0
Oit27	G1	Oct_480	G1	31/05/14	1189	375	1	B	0
Oit27	G1	Oct_480	G1	31/05/14	1189	375	1	C	0
Oit27	G1	Oct_480	G1	31/05/14	1189	375	1	D	0
Oit27	G1	Oct_480	G1	31/05/14	1190	378	2	A	0
Oit27	G1	Oct_480	G1	31/05/14	1190	378	2	B	0
Oit27	G1	Oct_480	G1	31/05/14	1190	378	2	C	0
Oit27	G1	Oct_480	G1	31/05/14	1190	378	2	D	0
Oit26	G1	Oct_506	G1	29/05/14	877	154	1	A	1
Oit26	G1	Oct_506	G1	29/05/14	877	154	1	B	1
Oit26	G1	Oct_506	G1	29/05/14	877	154	1	C	1
Oit26	G1	Oct_506	G1	29/05/14	877	154	1	D	1
Oit26	G1	Oct_506	G1	29/05/14	878	286	2	A	1
Oit26	G1	Oct_506	G1	29/05/14	878	286	2	B	1
Oit26	G1	Oct_506	G1	29/05/14	878	286	2	C	1
Oit26	G1	Oct_506	G1	29/05/14	878	286	2	D	0
Oit26	G1	Oct_506	G1	29/05/14	879	296	3	A	0
Oit26	G1	Oct_506	G1	29/05/14	879	296	3	B	0
Oit26	G1	Oct_506	G1	29/05/14	879	296	3	C	0
Oit26	G1	Oct_506	G1	29/05/14	879	296	3	D	0
Oit26	G1	Oct_508	G1	29/05/14	871	92	1	A	1
Oit26	G1	Oct_508	G1	29/05/14	871	92	1	B	0
Oit26	G1	Oct_508	G1	29/05/14	871	92	1	C	0
Oit26	G1	Oct_508	G1	29/05/14	871	92	1	D	0
Oit26	G1	Oct_508	G1	29/05/14	872	127	2	A	1
Oit26	G1	Oct_508	G1	29/05/14	872	127	2	B	1
Oit26	G1	Oct_508	G1	29/05/14	872	127	2	C	1
Oit26	G1	Oct_508	G1	29/05/14	872	127	2	D	1
Oit26	G1	Oct_508	G1	29/05/14	873	148	3	A	0
Oit26	G1	Oct_508	G1	29/05/14	873	148	3	B	0

Pollen Recepient reference	SI Group of pollen Recipient	Pollen donor reference	SI Group of pollen donor	Pollination date	Photo reference number	photo random number	N° of flower	Repetition of flower read	Class of SI phenotype
Oit26	G1	Oct_508	G1	29/05/14	873	148	3	C	0
Oit26	G1	Oct_508	G1	29/05/14	873	148	3	D	1
Oit27	G1	Oct_508	G1	31/05/14	1285	318	1	A	1
Oit27	G1	Oct_508	G1	31/05/14	1285	318	1	B	1
Oit27	G1	Oct_508	G1	31/05/14	1285	318	1	C	2
Oit27	G1	Oct_508	G1	31/05/14	1285	318	1	D	2
Oit27	G1	Oct_508	G1	31/05/14	1286	328	2	A	1
Oit27	G1	Oct_508	G1	31/05/14	1286	328	2	B	1
Oit27	G1	Oct_508	G1	31/05/14	1286	328	2	C	1
Oit27	G1	Oct_508	G1	31/05/14	1286	328	2	D	2
Oit30	G2	Oct_538	G2	31/05/14	1336	409	1	A	1
Oit30	G2	Oct_538	G2	31/05/14	1336	409	1	B	1
Oit30	G2	Oct_538	G2	31/05/14	1336	409	1	C	0
Oit30	G2	Oct_538	G2	31/05/14	1336	409	1	D	1
Oit30	G2	Oct_538	G2	31/05/14	1337	405	2	A	1
Oit30	G2	Oct_538	G2	31/05/14	1337	405	2	B	1
Oit30	G2	Oct_538	G2	31/05/14	1337	405	2	C	2
Oit30	G2	Oct_538	G2	31/05/14	1337	405	2	D	1
Oit30	G2	Oct_538	G2	31/05/14	1338	416	3	A	1
Oit30	G2	Oct_538	G2	31/05/14	1338	416	3	B	1
Oit30	G2	Oct_538	G2	31/05/14	1338	416	3	C	4
Oit30	G2	Oct_538	G2	31/05/14	1338	416	3	D	3
Oit15	G2	Oct_538	G2	29/05/14	924	273	1	A	1
Oit15	G2	Oct_538	G2	29/05/14	924	273	1	B	1
Oit15	G2	Oct_538	G2	29/05/14	924	273	1	C	1
Oit15	G2	Oct_538	G2	29/05/14	924	273	1	D	0
Oit15	G2	Oct_538	G2	29/05/14	925	290	2	A	0
Oit15	G2	Oct_538	G2	29/05/14	925	290	2	B	1
Oit15	G2	Oct_538	G2	29/05/14	925	290	2	C	0
Oit15	G2	Oct_538	G2	29/05/14	925	290	2	D	1
Oit15	G2	Oct_538	G2	29/05/14	926	244	3	A	1
Oit15	G2	Oct_538	G2	29/05/14	926	244	3	B	1
Oit15	G2	Oct_538	G2	29/05/14	926	244	3	C	0
Oit15	G2	Oct_538	G2	29/05/14	926	244	3	D	1
Oit30	G2	Oct_541	G2	31/05/14	1222	304	1	A	1
Oit30	G2	Oct_541	G2	31/05/14	1222	304	1	B	2
Oit30	G2	Oct_541	G2	31/05/14	1222	304	1	C	0
Oit30	G2	Oct_541	G2	31/05/14	1222	304	1	D	0
Oit30	G2	Oct_541	G2	31/05/14	1223	396	2	A	0
Oit30	G2	Oct_541	G2	31/05/14	1223	396	2	B	0
Oit30	G2	Oct_541	G2	31/05/14	1223	396	2	C	0
Oit30	G2	Oct_541	G2	31/05/14	1223	396	2	D	2
Oit15	G2	Oct_541	G2	27/05/14	761	30	1	A	8
Oit15	G2	Oct_541	G2	27/05/14	761	30	1	B	7
Oit15	G2	Oct_541	G2	27/05/14	761	30	1	C	6
Oit15	G2	Oct_541	G2	27/05/14	761	30	1	D	5
Oit15	G2	Oct_541	G2	27/05/14	762	173	2	A	4
Oit15	G2	Oct_541	G2	27/05/14	762	173	2	B	4
Oit15	G2	Oct_541	G2	27/05/14	762	173	2	C	5
Oit15	G2	Oct_541	G2	27/05/14	762	173	2	D	4
Oit15	G2	Oct_541	G2	27/05/14	763	20	3	A	8
Oit15	G2	Oct_541	G2	27/05/14	763	20	3	B	6
Oit15	G2	Oct_541	G2	27/05/14	763	20	3	C	7
Oit15	G2	Oct_541	G2	27/05/14	763	20	3	D	6
Oit27	G1	Oct_546	G1	31/05/14	1290	306	1	A	1
Oit27	G1	Oct_546	G1	31/05/14	1290	306	1	B	1
Oit27	G1	Oct_546	G1	31/05/14	1290	306	1	C	1
Oit27	G1	Oct_546	G1	31/05/14	1290	306	1	D	1

Pollen Recepient reference	SI Group of pollen Recipient	Pollen donor reference	SI Group of pollen donor	Pollination date	Photo reference number	photo random number	N° of flower	Repetition of flower read	Class of SI phenotype
Oit27	G1	Oct_546	G1	31/05/14	1291	390	2	A	1
Oit27	G1	Oct_546	G1	31/05/14	1291	390	2	B	1
Oit27	G1	Oct_546	G1	31/05/14	1291	390	2	C	1
Oit27	G1	Oct_546	G1	31/05/14	1291	390	2	D	1
Oit27	G1	Oct_546	G1	31/05/14	1292	353	3	A	1
Oit27	G1	Oct_546	G1	31/05/14	1292	353	3	B	1
Oit27	G1	Oct_546	G1	31/05/14	1292	353	3	C	1
Oit27	G1	Oct_546	G1	31/05/14	1292	353	3	D	1
Oit26	G1	Oct_547	G1	29/05/14	917	298	1	A	0
Oit26	G1	Oct_547	G1	29/05/14	917	298	1	B	0
Oit26	G1	Oct_547	G1	29/05/14	917	298	1	C	0
Oit26	G1	Oct_547	G1	29/05/14	917	298	1	D	0
Oit26	G1	Oct_547	G1	29/05/14	918	213	2	A	0
Oit26	G1	Oct_547	G1	29/05/14	918	213	2	B	0
Oit26	G1	Oct_547	G1	29/05/14	918	213	2	C	0
Oit26	G1	Oct_547	G1	29/05/14	918	213	2	D	0
Oit26	G1	Oct_547	G1	29/05/14	919	210	3	A	0
Oit26	G1	Oct_547	G1	29/05/14	919	210	3	B	0
Oit26	G1	Oct_547	G1	29/05/14	919	210	3	C	0
Oit26	G1	Oct_547	G1	29/05/14	919	210	3	D	0
Oit27	G1	Oct_547	G1	31/05/14	1329	323	1	A	1
Oit27	G1	Oct_547	G1	31/05/14	1329	323	1	B	1
Oit27	G1	Oct_547	G1	31/05/14	1329	323	1	C	1
Oit27	G1	Oct_547	G1	31/05/14	1329	323	1	D	1
Oit27	G1	Oct_547	G1	31/05/14	1330	341	2	A	1
Oit27	G1	Oct_547	G1	31/05/14	1330	341	2	B	1
Oit27	G1	Oct_547	G1	31/05/14	1330	341	2	C	0
Oit27	G1	Oct_547	G1	31/05/14	1330	341	2	D	0
Oit27	G1	Oct_547	G1	31/05/14	1331	392	3	A	1
Oit27	G1	Oct_547	G1	31/05/14	1331	392	3	B	1
Oit27	G1	Oct_547	G1	31/05/14	1331	392	3	C	1
Oit27	G1	Oct_547	G1	31/05/14	1331	392	3	D	1
Oit30	G2	Oct_603	G2	31/05/14	1160	335	1	A	1
Oit30	G2	Oct_603	G2	31/05/14	1160	335	1	B	1
Oit30	G2	Oct_603	G2	31/05/14	1160	335	1	C	1
Oit30	G2	Oct_603	G2	31/05/14	1160	335	1	D	2
Oit30	G2	Oct_603	G2	31/05/14	1161	336	2	A	1
Oit30	G2	Oct_603	G2	31/05/14	1161	336	2	B	2
Oit30	G2	Oct_603	G2	31/05/14	1161	336	2	C	2
Oit30	G2	Oct_603	G2	31/05/14	1161	336	2	D	1
Oit15	G2	Oct_603	G2	27/05/14	190	61	1	A	2
Oit15	G2	Oct_603	G2	27/05/14	190	61	1	B	2
Oit15	G2	Oct_603	G2	27/05/14	190	61	1	C	3
Oit15	G2	Oct_603	G2	27/05/14	190	61	1	D	3
Oit15	G2	Oct_603	G2	27/05/14	191	125	2	A	1
Oit15	G2	Oct_603	G2	27/05/14	191	125	2	B	1
Oit15	G2	Oct_603	G2	27/05/14	191	125	2	C	1
Oit15	G2	Oct_603	G2	27/05/14	191	125	2	D	1
Oit15	G2	Oct_603	G2	27/05/14	192	42	3	A	3
Oit15	G2	Oct_603	G2	27/05/14	192	42	3	B	3
Oit15	G2	Oct_603	G2	27/05/14	192	42	3	C	3
Oit15	G2	Oct_603	G2	27/05/14	192	42	3	D	3
Oit26	G1	Oct_630	G1	27/05/14	220	136	1	A	0
Oit26	G1	Oct_630	G1	27/05/14	220	136	1	B	0
Oit26	G1	Oct_630	G1	27/05/14	220	136	1	C	0
Oit26	G1	Oct_630	G1	27/05/14	220	136	1	D	0
Oit27	G1	Oct_630	G1	31/05/14	1183	358	1	A	1
Oit27	G1	Oct_630	G1	31/05/14	1183	358	1	B	1

Pollen Recepient reference	SI Group of pollen Recipient	Pollen donor reference	SI Group of pollen donor	Pollination date	Photo reference number	photo random number	N° of flower	Repetition of flower read	Class of SI phenotype
Oit27	G1	Oct_630	G1	31/05/14	1183	358	1	C	0
Oit27	G1	Oct_630	G1	31/05/14	1183	358	1	D	0
Oit27	G1	Oct_630	G1	31/05/14	1184	361	2	A	1
Oit27	G1	Oct_630	G1	31/05/14	1184	361	2	B	0
Oit27	G1	Oct_630	G1	31/05/14	1184	361	2	C	1
Oit27	G1	Oct_630	G1	31/05/14	1184	361	2	D	0
Oit27	G1	Oct_630	G1	31/05/14	1185	337	3	A	0
Oit27	G1	Oct_630	G1	31/05/14	1185	337	3	B	0
Oit27	G1	Oct_630	G1	31/05/14	1185	337	3	C	0
Oit27	G1	Oct_630	G1	31/05/14	1185	337	3	D	0
Oit26	G1	Oct_641	G1	27/05/14	209	135	1	A	1
Oit26	G1	Oct_641	G1	27/05/14	209	135	1	B	1
Oit26	G1	Oct_641	G1	27/05/14	209	135	1	C	2
Oit26	G1	Oct_641	G1	27/05/14	209	135	1	D	1
Oit26	G1	Oct_641	G1	27/05/14	210	29	2	A	2
Oit26	G1	Oct_641	G1	27/05/14	210	29	2	B	1
Oit26	G1	Oct_641	G1	27/05/14	210	29	2	C	1
Oit26	G1	Oct_641	G1	27/05/14	210	29	2	D	1
Oit27	G1	Oct_641	G1	31/05/14	1173	371	1	A	1
Oit27	G1	Oct_641	G1	31/05/14	1173	371	1	B	1
Oit27	G1	Oct_641	G1	31/05/14	1173	371	1	C	1
Oit27	G1	Oct_641	G1	31/05/14	1173	371	1	D	2
Oit27	G1	Oct_641	G1	31/05/14	1174	308	2	A	0
Oit27	G1	Oct_641	G1	31/05/14	1174	308	2	B	0
Oit27	G1	Oct_641	G1	31/05/14	1174	308	2	C	0
Oit27	G1	Oct_641	G1	31/05/14	1174	308	2	D	0
Oit30	G2	Oit28	G2	02/06/14	1495	410	1	A	2
Oit30	G2	Oit28	G2	02/06/14	1495	410	1	B	2
Oit30	G2	Oit28	G2	02/06/14	1495	410	1	C	3
Oit30	G2	Oit28	G2	02/06/14	1495	410	1	D	3
Oit30	G2	Oit28	G2	02/06/14	1496	443	2	A	1
Oit30	G2	Oit28	G2	02/06/14	1496	443	2	B	2
Oit30	G2	Oit28	G2	02/06/14	1496	443	2	C	2
Oit30	G2	Oit28	G2	02/06/14	1496	443	2	D	2
Oit30	G2	Oit01	G2	02/06/14	1458	406	1	A	1
Oit30	G2	Oit01	G2	02/06/14	1458	406	1	B	1
Oit30	G2	Oit01	G2	02/06/14	1458	406	1	C	2
Oit30	G2	Oit01	G2	02/06/14	1458	406	1	D	2
Oit30	G2	Oit01	G2	02/06/14	1459	402	2	A	2
Oit30	G2	Oit01	G2	02/06/14	1459	402	2	B	3
Oit30	G2	Oit01	G2	02/06/14	1459	402	2	C	3
Oit30	G2	Oit01	G2	02/06/14	1459	402	2	D	3
Oit15	G2	Oit01	G2	30/05/14	494	12	1	A	3
Oit15	G2	Oit01	G2	30/05/14	494	12	1	B	3
Oit15	G2	Oit01	G2	30/05/14	494	12	1	C	3
Oit15	G2	Oit01	G2	30/05/14	494	12	1	D	3
Oit15	G2	Oit01	G2	30/05/14	495	109	2	A	2
Oit15	G2	Oit01	G2	30/05/14	495	109	2	B	3
Oit15	G2	Oit01	G2	30/05/14	495	109	2	C	3
Oit15	G2	Oit01	G2	30/05/14	495	109	2	D	3
Oit15	G2	Oit01	G2	30/05/14	496	137	3	A	2
Oit15	G2	Oit01	G2	30/05/14	496	137	3	B	2
Oit15	G2	Oit01	G2	30/05/14	496	137	3	C	2
Oit15	G2	Oit01	G2	30/05/14	496	137	3	D	2
Oit15	G1	Oit02	G2	29/05/14	472	172	3	A	4
Oit15	G1	Oit02	G2	29/05/14	472	172	3	B	5
Oit15	G1	Oit02	G2	29/05/14	472	172	3	C	5
Oit15	G1	Oit02	G2	29/05/14	472	172	3	D	5

Pollen Recepient reference	SI Group of pollen Recipient	Pollen donor reference	SI Group of pollen donor	Pollination date	Photo reference number	photo random number	N° of flower	Repetition of flower read	Class of SI phenotype
Oit15	G1	Oit02	G2	29/05/14	474	146	4	A	4
Oit15	G1	Oit02	G2	29/05/14	474	146	4	B	4
Oit15	G1	Oit02	G2	29/05/14	474	146	4	C	5
Oit15	G1	Oit02	G2	29/05/14	474	146	4	D	5
Oit30	G2	Oit02	G2	02/06/14	1475	419	1	A	3
Oit30	G2	Oit02	G2	02/06/14	1475	419	1	B	3
Oit30	G2	Oit02	G2	02/06/14	1475	419	1	C	3
Oit30	G2	Oit02	G2	02/06/14	1475	419	1	D	3
Oit30	G2	Oit02	G2	02/06/14	1476	425	2	A	2
Oit30	G2	Oit02	G2	02/06/14	1476	425	2	B	4
Oit30	G2	Oit02	G2	02/06/14	1476	425	2	C	4
Oit30	G2	Oit02	G2	02/06/14	1476	425	2	D	4
Oit30	G2	Oit03	G2	02/06/14	1579	436	1	A	2
Oit30	G2	Oit03	G2	02/06/14	1579	436	1	B	4
Oit30	G2	Oit03	G2	02/06/14	1579	436	1	C	3
Oit30	G2	Oit03	G2	02/06/14	1579	436	1	D	3
Oit30	G2	Oit03	G2	02/06/14	1580	441	2	A	4
Oit30	G2	Oit03	G2	02/06/14	1580	441	2	B	6
Oit30	G2	Oit03	G2	02/06/14	1580	441	2	C	7
Oit30	G2	Oit03	G2	02/06/14	1580	441	2	D	7
Oit30	G2	Oit22	G2	02/06/14	1479	417	1	A	2
Oit30	G2	Oit22	G2	02/06/14	1479	417	1	B	3
Oit30	G2	Oit22	G2	02/06/14	1479	417	1	C	3
Oit30	G2	Oit22	G2	02/06/14	1479	417	1	D	3
Oit30	G2	Oit22	G2	02/06/14	1480	438	2	A	3
Oit30	G2	Oit22	G2	02/06/14	1480	438	2	B	3
Oit30	G2	Oit22	G2	02/06/14	1480	438	2	C	3
Oit30	G2	Oit22	G2	02/06/14	1480	438	2	D	3
Oit15	G2	Oit22	G2	30/05/14	540	99	1	A	1
Oit15	G2	Oit22	G2	30/05/14	540	99	1	B	1
Oit15	G2	Oit22	G2	30/05/14	540	99	1	C	0
Oit15	G2	Oit22	G2	30/05/14	540	99	1	D	2
Oit15	G2	Oit22	G2	30/05/14	541	17	2	A	3
Oit15	G2	Oit22	G2	30/05/14	541	17	2	B	3
Oit15	G2	Oit22	G2	30/05/14	541	17	2	C	3
Oit15	G2	Oit22	G2	30/05/14	541	17	2	D	4
Oit15	G2	Oit22	G2	30/05/14	542	174	3	A	3
Oit15	G2	Oit22	G2	30/05/14	542	174	3	B	5
Oit15	G2	Oit22	G2	30/05/14	542	174	3	C	4
Oit15	G2	Oit22	G2	30/05/14	542	174	3	D	5
Oit30	G2	Oit29	G2	02/06/14	1454	445	1	A	4
Oit30	G2	Oit29	G2	02/06/14	1454	445	1	B	4
Oit30	G2	Oit29	G2	02/06/14	1454	445	1	C	7
Oit30	G2	Oit29	G2	02/06/14	1454	445	1	D	5
Oit30	G2	Oit29	G2	02/06/14	1455	403	2	A	3
Oit30	G2	Oit29	G2	02/06/14	1455	403	2	B	4
Oit30	G2	Oit29	G2	02/06/14	1455	403	2	C	4
Oit30	G2	Oit29	G2	02/06/14	1455	403	2	D	4
Oit15	G2	Oit29	G2	30/05/14	556	171	1	A	2
Oit15	G2	Oit29	G2	30/05/14	556	171	1	B	3
Oit15	G2	Oit29	G2	30/05/14	556	171	1	C	3
Oit15	G2	Oit29	G2	30/05/14	556	171	1	D	3
Oit15	G2	Oit29	G2	30/05/14	557	158	2	A	3
Oit15	G2	Oit29	G2	30/05/14	557	158	2	B	4
Oit15	G2	Oit29	G2	30/05/14	557	158	2	C	4
Oit15	G2	Oit29	G2	30/05/14	557	158	2	D	3
Oit15	G2	Oit29	G2	30/05/14	558	6	3	A	5
Oit15	G2	Oit29	G2	30/05/14	558	6	3	B	3

Pollen Recepient reference	SI Group of pollen Recipient	Pollen donor reference	SI Group of pollen donor	Pollination date	Photo reference number	photo random number	N° of flower	Repetition of flower read	Class of SI phenotype
Oit15	G2	Oit29	G2	30/05/14	558	6	3	C	4
Oit15	G2	Oit29	G2	30/05/14	558	6	3	D	4
Oit30	G2	Oit36	G2	02/06/14	1467	439	1	A	2
Oit30	G2	Oit36	G2	02/06/14	1467	439	1	B	2
Oit30	G2	Oit36	G2	02/06/14	1467	439	1	C	2
Oit30	G2	Oit36	G2	02/06/14	1467	439	1	D	2
Oit30	G2	Oit36	G2	02/06/14	1468	401	2	A	2
Oit30	G2	Oit36	G2	02/06/14	1468	401	2	B	2
Oit30	G2	Oit36	G2	02/06/14	1468	401	2	C	2
Oit30	G2	Oit36	G2	02/06/14	1468	401	2	D	2
Oit15	G2	Oit36	G2	30/05/14	508	66	1	A	2
Oit15	G2	Oit36	G2	30/05/14	508	66	1	B	3
Oit15	G2	Oit36	G2	30/05/14	508	66	1	C	2
Oit15	G2	Oit36	G2	30/05/14	508	66	1	D	2
Oit15	G2	Oit36	G2	30/05/14	509	132	2	A	4
Oit15	G2	Oit36	G2	30/05/14	509	132	2	B	4
Oit15	G2	Oit36	G2	30/05/14	509	132	2	C	4
Oit15	G2	Oit36	G2	30/05/14	509	132	2	D	4
Oit15	G2	Oit36	G2	30/05/14	510	107	3	A	2
Oit15	G2	Oit36	G2	30/05/14	510	107	3	B	3
Oit15	G2	Oit36	G2	30/05/14	510	107	3	C	3
Oit15	G2	Oit36	G2	30/05/14	510	107	3	D	3
Oit30	G2	Oit38	G2	02/06/14	1570	437	1	A	3
Oit30	G2	Oit38	G2	02/06/14	1570	437	1	B	4
Oit30	G2	Oit38	G2	02/06/14	1570	437	1	C	4
Oit30	G2	Oit38	G2	02/06/14	1570	437	1	D	3
Oit30	G2	Oit38	G2	02/06/14	1571	444	2	A	2
Oit30	G2	Oit38	G2	02/06/14	1571	444	2	B	2
Oit30	G2	Oit38	G2	02/06/14	1571	444	2	C	2
Oit30	G2	Oit38	G2	02/06/14	1571	444	2	D	2
Oit30	G2	Oit38	G2	02/06/14	1572	427	3	A	2
Oit30	G2	Oit38	G2	02/06/14	1572	427	3	B	3
Oit30	G2	Oit38	G2	02/06/14	1572	427	3	C	3
Oit30	G2	Oit38	G2	02/06/14	1572	427	3	D	3
Oit15	G2	Oit38	G2	01/01/13	6766	2	1	A	3
Oit15	G2	Oit38	G2	01/01/13	6766	2	1	B	3
Oit15	G2	Oit38	G2	01/01/13	6766	2	1	C	3
Oit15	G2	Oit38	G2	01/01/13	6766	2	1	D	3
Oit15	G2	Oit38	G2	01/01/13	6767	15	1	A	3
Oit15	G2	Oit38	G2	01/01/13	6767	15	1	B	3
Oit15	G2	Oit38	G2	01/01/13	6767	15	1	C	3
Oit15	G2	Oit38	G2	01/01/13	6767	15	1	D	3
Oit30	G2	SP_008	G2	01/06/14	1386	432	1	A	2
Oit30	G2	SP_008	G2	01/06/14	1386	432	1	B	3
Oit30	G2	SP_008	G2	01/06/14	1386	432	1	C	3
Oit30	G2	SP_008	G2	01/06/14	1386	432	1	D	3
Oit30	G2	SP_008	G2	01/06/14	1387	404	2	A	2
Oit30	G2	SP_008	G2	01/06/14	1387	404	2	B	2
Oit30	G2	SP_008	G2	01/06/14	1387	404	2	C	2
Oit30	G2	SP_008	G2	01/06/14	1387	404	2	D	2
Oit15	G2	SP_008	G2	29/05/14	1042	272	1	A	3
Oit15	G2	SP_008	G2	29/05/14	1042	272	1	B	4
Oit15	G2	SP_008	G2	29/05/14	1042	272	1	C	4
Oit15	G2	SP_008	G2	29/05/14	1042	272	1	D	4
Oit15	G2	SP_008	G2	29/05/14	1043	255	2	A	3
Oit15	G2	SP_008	G2	29/05/14	1043	255	2	B	3
Oit15	G2	SP_008	G2	29/05/14	1043	255	2	C	3
Oit15	G2	SP_008	G2	29/05/14	1043	255	2	D	4

Pollen Recepient reference	SI Group of pollen Recipient	Pollen donor reference	SI Group of pollen donor	Pollination date	Photo reference number	photo random number	N° of flower	Repetition of flower read	Class of SI phenotype
Oit15	G2	SP_008	G2	29/05/14	1044	295	3	A	3
Oit15	G2	SP_008	G2	29/05/14	1044	295	3	B	4
Oit15	G2	SP_008	G2	29/05/14	1044	295	3	C	4
Oit15	G2	SP_008	G2	29/05/14	1044	295	3	D	4
Oit26	G1	SP_017	G1	29/05/14	470	151	1	A	1
Oit26	G1	SP_017	G1	29/05/14	470	151	1	B	0
Oit26	G1	SP_017	G1	29/05/14	470	151	1	C	0
Oit26	G1	SP_017	G1	29/05/14	470	151	1	D	2
Oit26	G1	SP_017	G1	29/05/14	471	60	2	A	1
Oit26	G1	SP_017	G1	29/05/14	471	60	2	B	1
Oit26	G1	SP_017	G1	29/05/14	471	60	2	C	2
Oit26	G1	SP_017	G1	29/05/14	471	60	2	D	0
Oit27	G1	SP_017	G1	01/06/14	1435	433	1	A	No pollen
Oit27	G1	SP_017	G1	01/06/14	1435	433	1	B	No pollen
Oit27	G1	SP_017	G1	01/06/14	1435	433	1	C	No pollen
Oit27	G1	SP_017	G1	01/06/14	1435	433	1	D	No pollen
Oit30	G2	SP_039	G2	01/06/14	1394	422	1	A	2
Oit30	G2	SP_039	G2	01/06/14	1394	422	1	B	2
Oit30	G2	SP_039	G2	01/06/14	1394	422	1	C	3
Oit30	G2	SP_039	G2	01/06/14	1394	422	1	D	3
Oit30	G2	SP_039	G2	01/06/14	1395	442	2	A	2
Oit30	G2	SP_039	G2	01/06/14	1395	442	2	B	3
Oit30	G2	SP_039	G2	01/06/14	1395	442	2	C	3
Oit30	G2	SP_039	G2	01/06/14	1395	442	2	D	2
Oit15	G2	SP_039	G2	29/05/14	1047	216	1	A	3
Oit15	G2	SP_039	G2	29/05/14	1047	216	1	B	4
Oit15	G2	SP_039	G2	29/05/14	1047	216	1	C	3
Oit15	G2	SP_039	G2	29/05/14	1047	216	1	D	3
Oit15	G2	SP_039	G2	29/05/14	1048	281	2	A	3
Oit15	G2	SP_039	G2	29/05/14	1048	281	2	B	5
Oit15	G2	SP_039	G2	29/05/14	1048	281	2	C	5
Oit15	G2	SP_039	G2	29/05/14	1048	281	2	D	5
Oit15	G2	SP_039	G2	29/05/14	1049	276	3	A	3
Oit15	G2	SP_039	G2	29/05/14	1049	276	3	B	4
Oit15	G2	SP_039	G2	29/05/14	1049	276	3	C	4
Oit15	G2	SP_039	G2	29/05/14	1049	276	3	D	4
Oit30	G2	SP_045	G2	01/06/14	1441	418	1	A	7
Oit30	G2	SP_045	G2	01/06/14	1441	418	1	B	9
Oit30	G2	SP_045	G2	01/06/14	1441	418	1	C	9
Oit30	G2	SP_045	G2	01/06/14	1441	418	1	D	9
Oit30	G2	SP_045	G2	01/06/14	1442	435	2	A	3
Oit30	G2	SP_045	G2	01/06/14	1442	435	2	B	4
Oit30	G2	SP_045	G2	01/06/14	1442	435	2	C	4
Oit30	G2	SP_045	G2	01/06/14	1442	435	2	D	4
Oit15	G2	SP_045	G2	29/05/14	1012	207	2	A	3
Oit15	G2	SP_045	G2	29/05/14	1012	207	2	B	4
Oit15	G2	SP_045	G2	29/05/14	1012	207	2	C	4
Oit15	G2	SP_045	G2	29/05/14	1012	207	2	D	4
Oit15	G2	SP_045	G2	29/05/14	1013	240	3	A	3
Oit15	G2	SP_045	G2	29/05/14	1013	240	3	B	5
Oit15	G2	SP_045	G2	29/05/14	1013	240	3	C	5
Oit15	G2	SP_045	G2	29/05/14	1013	240	3	D	4
Oit30	G2	SP_052	G2	01/06/14	1398	434	1	A	2
Oit30	G2	SP_052	G2	01/06/14	1398	434	1	B	3
Oit30	G2	SP_052	G2	01/06/14	1398	434	1	C	2
Oit30	G2	SP_052	G2	01/06/14	1398	434	1	D	2
Oit30	G2	SP_052	G2	01/06/14	1399	415	2	A	2
Oit30	G2	SP_052	G2	01/06/14	1399	415	2	B	3

Pollen Recepient reference	SI Group of pollen Recipient	Pollen donor reference	SI Group of pollen donor	Pollination date	Photo reference number	photo random number	N° of flower	Repetition of flower read	Class of SI phenotype
Oit30	G2	SP_052	G2	01/06/14	1399	415	2	C	3
Oit30	G2	SP_052	G2	01/06/14	1399	415	2	D	3
Oit15	G2	SP_052	G2	29/05/14	1060	200	1	A	4
Oit15	G2	SP_052	G2	29/05/14	1060	200	1	B	7
Oit15	G2	SP_052	G2	29/05/14	1060	200	1	C	6
Oit15	G2	SP_052	G2	29/05/14	1060	200	1	D	6
Oit15	G2	SP_052	G2	29/05/14	1061	297	2	A	4
Oit15	G2	SP_052	G2	29/05/14	1061	297	2	B	5
Oit15	G2	SP_052	G2	29/05/14	1061	297	2	C	6
Oit15	G2	SP_052	G2	29/05/14	1061	297	2	D	5
Oit15	G2	SP_052	G2	29/05/14	1062	221	3	A	7
Oit15	G2	SP_052	G2	29/05/14	1062	221	3	B	8
Oit15	G2	SP_052	G2	29/05/14	1062	221	3	C	8
Oit15	G2	SP_052	G2	29/05/14	1062	221	3	D	7
